# Interventions for the prevention of spontaneous preterm birth: a scoping review of systematic reviews

**DOI:** 10.1136/bmjopen-2021-052576

**Published:** 2022-05-13

**Authors:** Fiona Campbell, Shumona Salam, Anthea Sutton, Shamanthi Maya Jayasooriya, Caroline Mitchell, Emmanuel Amabebe, Julie Balen, Bronwen M Gillespie, Kerry Parris, Priya Soma-Pillay, Lawrence Chauke, Brenda Narice, Dilichukwu O Anumba

**Affiliations:** 1 ScHARR, The University of Sheffield, Sheffield, UK; 2 Department of Oncology and Metabolism, The University of Sheffield, Sheffield, UK; 3 Academic Unit of Primary Medical Care, The University of Sheffield, Sheffield, UK; 4 Steve Biko Academic Hospital, University of Pretoria, Pretoria, South Africa; 5 Department of Obstetrics and Gynaecology, University of Witwatersrand, Johannesburg, South Africa

**Keywords:** Maternal medicine, International health services, Public health, Health policy

## Abstract

**Background:**

Globally, 11% of babies are born preterm each year. Preterm birth (PTB) is a leading cause of neonatal death and under-five mortality and morbidity, with lifelong sequelae in those who survive. PTB disproportionately impacts low/middle-income countries (LMICs) where the burden is highest.

**Objectives:**

This scoping review sought to the evidence for interventions that reduce the risk of PTB, focusing on the evidence from LMICs and describing how context is considered in evidence synthesis.

**Design:**

We conducted a scoping review, to describe this wide topic area. We searched five electronic databases (2009–2020) and contacted experts to identify relevant systematic reviews of interventions to reduce the risk of PTB. We included published systematic reviews that examined the effectiveness of interventions and their effect on reducing the risk of PTB. Data were extracted and is described narratively.

**Results:**

139 published systematic reviews were included in the review. Interventions were categorised as primary or secondary. The interventions where the results showed a greater effect size and consistency across review findings included treatment of syphilis and vaginal candidiasis, vitamin D supplementation and cervical cerclage. Included in the 139 reviews were 1372 unique primary source studies. 28% primary studies were undertaken in LMIC contexts and only 4.5% undertaken in a low-income country (LIC) Only 10.8% of the reviews sought to explore the impact of context on findings, and 19.4% reviews did not report the settings or the primary studies.

**Conclusion:**

This scoping review highlights the lack of research evidence derived from contexts where the burden of PTB globally is greatest. The lack of rigour in addressing contextual applicability within systematic review methods is also highlighted. This presents a risk of inappropriate and unsafe recommendations for practice within these contexts. It also highlights a need for primary research, developing and testing interventions in LIC settings.

**Strengths and limitations of this study**

- Scoping review methodology enabled us to look at a broad topic area and analyse how context is taken into account in the included systematic reviews. Primary studies not reported in systematic reviews will therefore have not been included in our analysis.
- We were not able to identify the setting of all primary studies where this was not reported and there is a risk that some studies, which have multiple publications may have been double counted.
- We only included systematic reviews published in English.

## Background

Preterm birth (PTB) is a global and public health priority. It is defined by the WHO as delivery before 37 completed weeks of gestation, with extremely preterm delivery defined as occurring at less than 28 weeks, very preterm delivery occurring between 28 and 32 weeks, and moderate to late preterm delivery occurring from 32 through 36 weeks.^1^ It is one of the leading causes of neonatal death and under-five mortality and morbidity, with lifelong sequelae.^2^ Children born prematurely have increased risks of cognitive problems, such as academic underachievement, behavioural problems and cerebral palsy than those born at full term.^3^ They are more likely to experience hospital admission due to infection, particularly during infancy.^4^ For parents, the financial, social and emotional effects are devastating.^3^

The global burden of preterm birth (PTB) is falling more heavily on countries with fewer resources to manage the medical, social and economic complexities of caring for premature infants. Globally, there are approximately 15 million live PTBs each year, which is estimated to be about 11% of all deliveries each year, ranging from about 8.7% in northern Europe to 13.4% in North Africa.^5 6^ The majority of PTBs occur in low/middle-income countries (LMICs).^6^ The highest PTB rates in 2014 occurred in Southeast Asia, South Asia and sub-Saharan Africa. Nine of the 11 countries with the highest rates were in Africa. Furthermore, 60% of all PTBs were estimated to have occurred in sub-Saharan Africa and South Asia accounting for just over 9 million of the almost 15 million PTBs that occurred worldwide in 2010 resulting in a PTB rate of 12.8% in those settings.

Patterns of PTB differ between high-income countries (HICs) and LMICs. However, the differences in these patterns, causes and distribution of PTB is unclear and have not been fully explored. PTB is multifactorial in its aetiology and has distinct biological pathways. The aetiologies differ according to gestational age, ethnicity and characteristics unique to each population. In order to redress the burden of PTB in LMICs, additional insight into the causative and associated factors in these settings is required.

While a number of reviews and overviews of reviews of interventions to reduce the risk of PTB have been undertaken,^7–10^ none have explored how many of the primary studies included in these reviews were undertaken in LMIC contexts. It is clear that some interventions that are effective in HIC contexts but may be harmful in LMIC settings, such as the use of antenatal corticosteroids^11^ and cerclage.^12^ It is also possible that treatments effective in HIC contexts may be even more beneficial or appropriate in LMIC contexts, such as nutritional supplements, interventions to increase birth spacing or interventions to improve the accuracy of measuring gestational age.

We have undertaken a broad scoping review of systematic reviews on interventions to reduce the risk of PTB identifying primary studies undertaken in LMICs. This will allow us to identify potential areas for further synthesis of the evidence and also to identify gaps in the research in order to direct future primary research.

### Review objectives

1. To identify systematic reviews that have sought to explore the effectiveness, safety and acceptability of interventions to prevent PTB.
2. To map research evidence to global settings to identify the geographical and economic contexts in which evidence is derived.
3. To identify where gaps in the research base exist (for real world, effectiveness, pragmatic studies) in LMIC contexts to inform future research and to generate research priorities.
4. To describe the methods used in meta-analysis to take into account geographical and regional differences in PTB.

## Methods

We used a scoping review methodology^13^ to describe the existing evidence (systematic reviews) available across primary and secondary interventions to prevent PTB, published between 2009 and 2020. Systematic scoping draws on methods described by Arksey and O’Malley^14^ for scoping reviews: ‘[…] a form of knowledge synthesis that addresses an exploratory research question aimed at scoping key concepts, types of evidence, and gaps in research related to a defined area or field by systematically searching, selecting, and synthesizing existing knowledge’.^14^ The approach enabled us to highlight the evidence gap and to assist with simultaneously undertaking a research prioritisation exercise and guideline development, as well as to inform a broader programme of research that aimed to develop effective postnatal interventions to mitigate PTB in LMIC settings. It also enabled us to generate a mega-map, an interactive table supported on our project website and designed as a visual tool to identify research gaps and facilitate ready access to relevant evidence (https://www.primeglobalhealth.co.uk/evidence-map-2-7-2020.html).

### Identifying relevant studies

Relevant systematic reviews were identified by systematic searches in the following electronic databases: Ovid MEDLINE, Cochrane Database of Systematic Reviews, PsycINFO via Ovid, EMBASE via Ovid and CINAHL via EBSCO. Each database was searched using the database thesaurus and the key word/free text method with terms relating to PTB combined with a systematic reviews filter. The search strategy incorporated the following limitations: articles written in English, and Human studies only from April 2009 to July 2020. Relevant systematic reviews were identified by systematic searches in the following electronic databases: MEDLINE, The Cochrane Library, PsycINFO, EMBASE and CINAHL. Each database was searched using the database thesaurus and the key word/free text method. The search strategy incorporated the following limitations: articles written in English, and Human studies only from April 2009 to July 2020. The date limit was selected due to the existence of a previous review for which the studies were conducted in April 2009.^15^ Full search strategies have been described and published.^16^

We began with a framework of interventions identified by two existing reviews^7 8^ as these were broad in their focus and encompassed a range of interventions. Any new intervention types identified during the screening process were then added to the map.

The process of study selection was based on inclusion and exclusion criteria as described in box 1. After removal of duplicates and irrelevant studies, based on the titles and abstracts, all potentially relevant reviews were read in full. Citations were screened by two reviewers (FC and one of the following team members SS, SMJ, EA, JB, BMG, BN, KP) independently and differences were resolved by discussion.

Box 1 **Inclusion/exclusion criteria based on PICOS**

**Population**

- Pregnant women at less than 37 completed weeks gestation without signs of threatened preterm labour or premature rupture of membranes.
- Excluded reviews where the study population was defined by comorbidities.

**Intervention**

- All interventions deliverable during pregnancy to prevent spontaneous preterm birth (PTB) (these included clinical, behavioural and nutritional interventions and health systems and policy interventions).
- All interventions assessed the risk of PTB.
- Excluded interventions given to pregnant women to improve neonatal outcomes.

**Comparators**

- We included any comparator, including placebo or alternative treatments.

**Outcomes**

- We included reviews which focused on PTB as an outcome.
- Where it is reported, we state how many of the primary studies measured PTB as an outcome and the resulting data used in the synthesis.

**Study design**

- Systematic reviews published between April 2009 and July 2020, of studies that have evaluated interventions to prevent PTB, or that measured PTB as a relevant outcome.

**Outcomes**

- PTB (<28, <34, <37 weeks gestation).
- We recorded neonatal outcomes and adverse outcomes if reported within the review.

### Data extraction and coding

Data were extracted using an agreed and piloted template and coded in Excel by two reviewers working independently (FC and one of the following team members SS, SMJ, EA, JB, BMG, BN, KP) differences were resolved by discussion. The following data categories were extracted: number of included studies, review PICO, setting of primary studies and any analysis that took into account study setting or population characteristics, PTB outcomes, assessment of adverse effects and recommendations for practice and research. PTB rates in low-income countries (LICs), lower middle-income countries (LMCs), upper middle-income countries (UMCs) and HICs settings were drawn from data published in a rigorous review of national civil registration and vital statistics to determine global, regional and national estimates of levels of PTB.^6^

Where reported information allowed, we used the World Bank categories to identify the categories of all country settings identified in the reviews.^17^

The population, interventions, comparators, outcomes and reviewer conclusions for future research were tabulated and described narratively. The country or countries of the included primary studies were noted, and the methods used in the review for analyses of data from different settings was also recorded and described. We did not contact review authors for missing data.

### Patient and public involvement

This review was undertaken as part of a larger programme of research in PTB (NIHR Global Health under grant (17/63/26)). The programme iPatient and public involvements informed by key stakeholders and a patient and public involvement (PPI) advisory group comprising representatives from Sheffield, Bangladesh, and South Africa. The design and questions for the review were informed by consultation with these groups.

## Results

**Figure 1:**
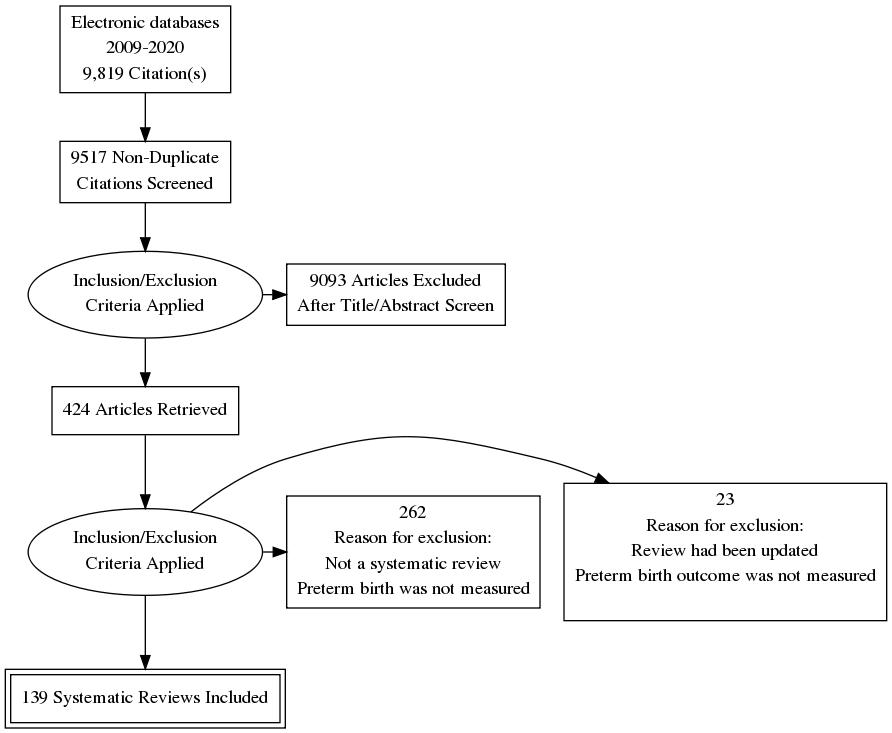
Flow of studies through review process.

Our search identified 3133 citations which were screened by two reviewers. A third reviewer was also involved where there was a lack of consensus or uncertainty regarding inclusion. Following screening, 424 full text papers were retrieved for data extraction. At data extraction a further 285 were excluded. The process of identifying the included reviews is summarised in figure 1.

We included 139 reviews which addressed a range of primary and secondary interventions and measured the effectiveness of the intervention in reducing the risk of PTB. These are summarised in table 1. There was a considerable variation in the number of included studies in the reviews for each intervention, reflecting differing research questions objectives (therefore different PICOs) and search strategies.

**Table 1:** Summary of included systematic reviews and settings of primary studies included in the review

Interventions	Number of reviews	Number of primary studies	Country NR	Country of primary study					Studies where setting NK
				LI	LM	UM	HI	Mixed	
**Primary prevention interventions**									
**Health systems**									
Models of antenatal care delivery (group/specialised)^61–71^	11	68	2	0	2	2	64	0	0
Midwifery led care^72^	1	15	0	0	0	0	15	0	0
Improving ANC coverage^28^	1	34	0	10	15	5	0	0	0
**Health behaviours**									
Smoking cessation^35 73^	2	111	0	0	0	1	110	0	0
Weight management^21 74–78^	6	70	1	0	2	8	60	0	0
**Nutritional interventions**									
Macronutrient supplements^29 30^	2	34	0	3	9	10	8	4	0
Micronutrient supplements^21 27–31 35–40 67–86^	33	481	2	29	82	122	214	6	9
Vitamin D^27 31 36 79–81^	6	75							
Vitamin A^37 82^	2	24							
Vitamin E, C, E and C^38 39 83^	3	67							
Iron, folic acid, iron and folic acid^40 84–90^	8	182							
Fish oil^91–95^	5	38							
Zinc^32 96^	2	25							
Calcium^41 42^	2	27							
Iodine^97^	2	14							
Multiple micronutrients^43 44 98^	3	29							
**Screening and treatment of periodontal disease** 99–110	12	46	0	0	3	7	36	0	0
**Screening and prevention/treatment of infection**	14	91	2	2	2	6	79	0	2
Asymptomatic bacteriuria^23 111–113^	4								
Screening and antibiotics for syphilis^24^	1								
Influenza vaccine^114 115^	2								
Lower genital tract infection^26^	1								
UTI^116 117^	2								
Vaginal candidiasis^25^	1								
Non-specific infection^118 119^	2								
Malaria^33 120 121^	3	17	0	8	7	2	2	0	0
**Secondary prevention interventions**									
Cerclage^18 22 45 122–136^	18	123	10	0	7	11	42		51
Bed rest^137–139^	3	40	1	4	0	0	36	0	0
Cervical pessary^140–145^	6	16	0	0	0	1	14	1	0
Progesterone^19 20 146–159^	16	59	5	1	7	8	28	4	11
Tocolytics^160–172^	11	167	3	1	0	13	68	0	84

ANC, antenatal care; HI, high income; LI, low income; LM, low middle; NK, not known; NR, not reported; UM, upper middle; UTI, urinary tract infection.

### Context of primary studies

A total of 1372 primary studies were included across all of the 139 reviews Not all of these studies will have been measuring PTB as an outcome but were included within the review which may have been measuring a range of maternal outcomes including PTB. The largest number of primary studies were those evaluating micronutrient supplements (n=481) and tocolytics (n=167). A total of 113 of the reviews described the country in which the primary studies were undertaken and so these data were known for 1288 (93.9%) of 1372 included primary studies. Of these, 390 (30.3%) were undertaken in LMICs, 15 primary studies were multicentre and included data gathered from LMIC and HIC settings, though only 3 of these studies included LICs. Of the studies undertaken in LMICs, a majority (n=255) examined the effects of nutritional supplements. Excluding nutritional intervention studies, the proportion of LMIC-based primary studies of interventions to reduce PTB accounts for only (n=135) 10.5% of the included studies where settings are known.

Of the total number of primary studies undertaken in LMIC contexts, those studies undertaken in LIC settings represented a very small proportion of included studies. Participants from LICs were represented in only 4.5% (n=58) of the total number of studies, and if the nutritional intervention studies are excluded, they account for only 2.5% (n=32) of the studies evaluating interventions. Of those primary studies that were undertaken in LMIC settings the numbers within each country category differed significantly. The proportion of the studies that are undertaken in LIC, LMC and UMC were 14.9% (n=58), 34.8% (n=136) and 50.2% (n=196), respectively. There are only single trials that have evaluated the impact of progesterone, tocolytics and interventions to increase calorie intake in LIC settings. There are no trials that have evaluated smoking cessation, preventing excessive weight gain, prevention and treatment of periodontal disease, influenza vaccine and cervical pessaries. The number of trials in each of the country categories within each intervention type are shown in table 1.

**Figure 2:**
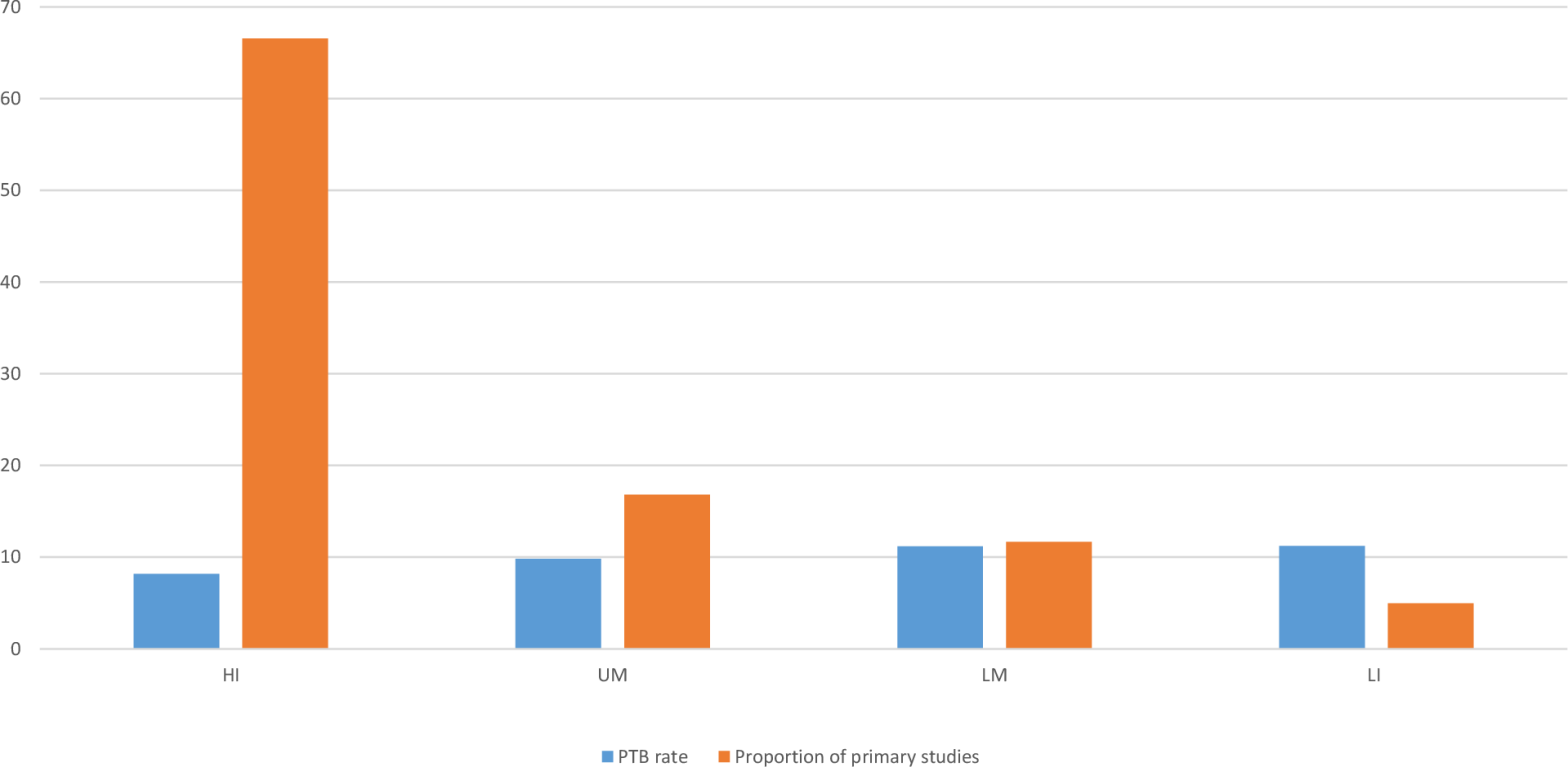
Rates of PTB and proportion of primary studies undertaken in each setting. HI, high income; LI, low income; LM, low middle; PTB, preterm birth; UM, upper middle.

When these data are compared alongside data that shows the prevalence of PTB globally it is clear that there is an inverse pattern in the distribution of the data (figure 2).

### Effectiveness of interventions

**Figure 3:**
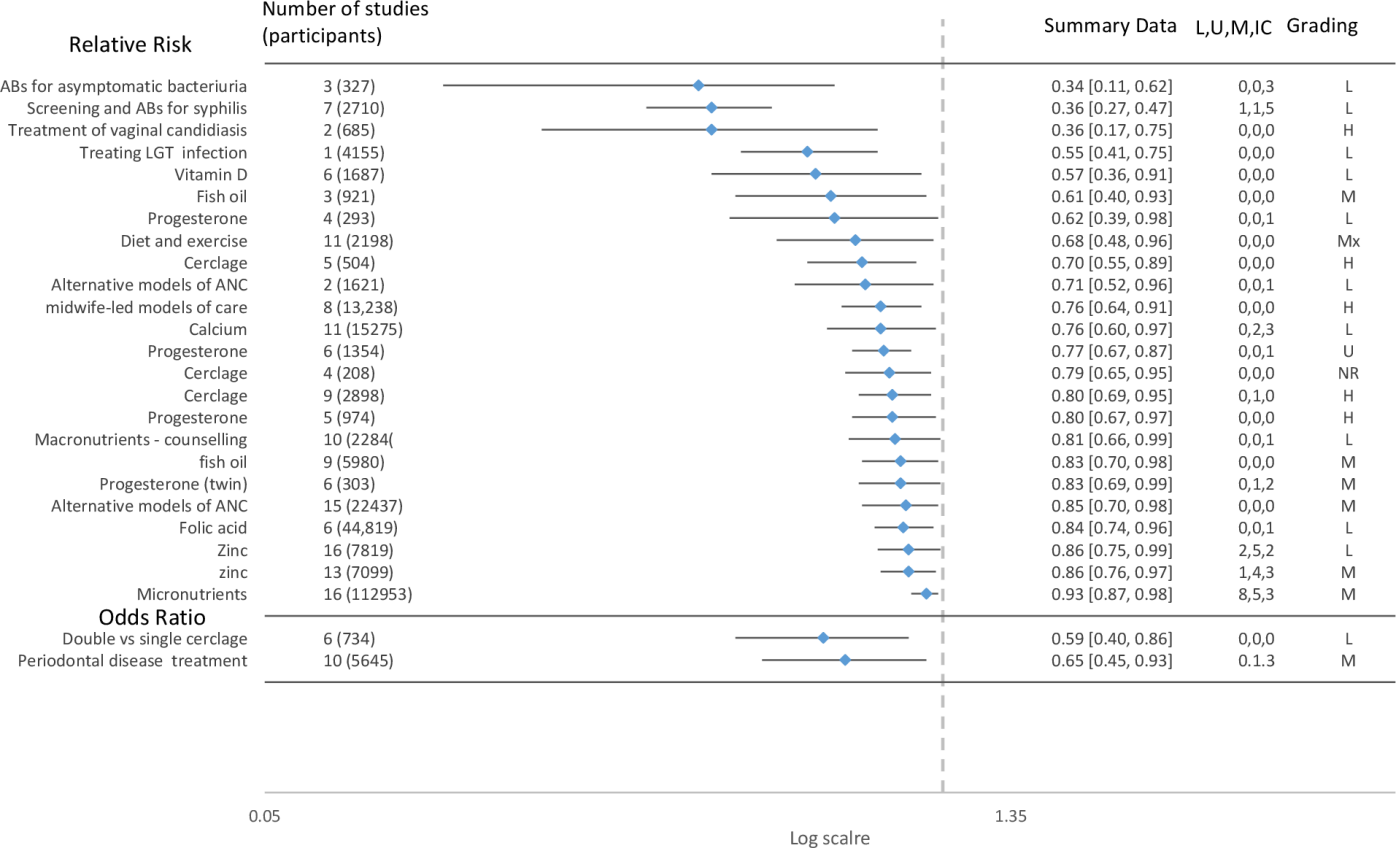
Summary results of systematic reviews of interventions showing reduction in risk of preterm birth. ANC, antenatal care; L, U, M, IC, low, low middle, upper middle-income countries; LGT, lower genital tract; RR, relative risk.

The effectiveness of interventions in reducing the risk of PTB was variable with no intervention showing consistent effectiveness across the included reviews. Although interpretation of these data is limited by the lack of quality appraisal of the included reviews, and therefore should be viewed with caution. Overall, the scoping review demonstrates considerable inconsistency of results of interventions. Of the 139 reviews, 28 reported a reduction in PTB in intervention versus control, 80% (n=111) of the reviews found that the intervention had no impact in reducing the risk of PTB. The summary result (relative risk (RR) and OR are shown in figure 3). The results show the reduction in PTB less than 37 weeks gestation. In three reviews the intervention was not statistically significant at 37 weeks but was reported as statistically significant at 34 weeks,^18^ 35 weeks^19^ and 36 weeks^20^. Two reviews reported a positive effect of the intervention in reducing risk of PTB but reported the outcome on a continuous measure. These included the effectiveness of macronutrient supplements^21^ (SMD −0.19 (95% CI −0.34 to −0.04)) and cerclage (mean difference 95% CI 33.98 days (17.88 to 50.08)).^22^ The interventions reporting binary outcomes which appear to have the greatest effect (RR=0.2–0.4) in reducing PTB are: antibiotics for asymptomatic bacteriuria^23^ (RR=0.34 (95% CI 0.11 to 0.62), the screening and treatment of syphilis^24^ (RR=0.36 (95% CI 0.27 to 0.47), and treatment of vaginal candidiasis^25^ (RR=0.36, (95% CI 0.17 to 0.75). Interventions with moderate effects (RR=0.4–0.6) included treating lower genital tract infection^26^ and vitamin D supplements.^27^ Four of the reviews (figure 2) with a positive effect of the intervention considered that the strength of evidence supporting the finding could be considered high and the finding reliable. None of these reviews included studies conducted in LIC settings, and only one included one study in an LMIC.

### Dealing with context and generalisability within evidence synthesis

The authors of the included reviews used different approaches to dealing with the contextual variation when pooling data from primary studies, which was either to ignore, document, explore or control for differences. Twenty-seven reviews (23.8%) did not describe the setting of the primary study, ignoring variation in outcomes that may arise as a result of these differences. This occurred most frequently in reviews of cervical cerclage (see table 1). The majority of the included reviews 86 (76.1%) documented the country in which the primary study was carried out either within the text, tables of study characteristics or in accompanying appendices, but this was not considered further in terms of its implications for the findings, or application for future practice or research.

Eight reviews^27–34^ sought to explore the impact of geographical and economic context by undertaking a subgroup analysis comparing trials conducted in low income settings with those in high income settings or regression analysis with geographical regions as covariates (Africa, Americas, Southeast Asia, Europe, Eastern Mediterranean, Western Pacific). In addition, one study^34^ listed the country instead of the author name on the forest plot allowing ready visualisation of differences across settings. Nine reviews^35–43^ undertook subgroup analysis based on features of the population that might vary across settings and influence the effectiveness of the intervention, such as baseline nutritional status of the mother. One review^44^ exploring multiple micronutrient supplementation controlled for settings by limiting the review to include only those studies undertaken in LMIC contexts. Four reviews^19 20 44 45^ undertook an IPD (individual patient data) analysis, allowing subgroup analyses about differences in effect more easily than with aggregate data. This approach allowed comparison between effects for women recruited and receiving the intervention in different settings, effect sizes in each country could also be shown in the analyses.

## Discussion

This scoping review has revealed an inverse pattern of research, with only 30.3% of published research included in systematic reviews of interventions reporting PTB outcomes carried out in LMIC settings, and only 4.5% was conducted in the poorest countries in the world where the burden of PTB is greatest. The distribution of types of intervention tested and evaluated in these settings is not even across interventions, but is largely focused on very context specific interventions (prevention of malarial infection) and nutritional supplementation. Similar patterns of a mismatch between research effort and health needs in non-high income regions have been identified across a broad range of diseases.^46 47^ It has also been previously reported that primary research often fails to capture those with the greatest healthcare needs such as vulnerable populations.^48 49^

This review has also revealed a limited approach in evidence synthesis to explore the applicability of findings across geographical settings and to draw attention to these gaps with a resultant risk that interventions shown to be effective in HI settings may not translate to LIC settings and may indeed have adverse effects when applied to LIC settings. Likewise, the focus of research in HIC settings means that interventions that may have greater benefit in LIC settings—where the problem is greatest—remain untested or replicated with larger numbers of participants. Adolescent pregnancy and short inter pregnancy intervals, both of which are more common in LMICs, have been highlighted as important risk factors for PTB^50^ yet there is a lack of data on interventions to address these and their effectiveness in reducing the risk of PTB.

The lack of robust evidence to inform both the primary and secondary prevention of PTB in LIC settings, where the prevalence of PTB is highest presents challenges for developing appropriate and contextually relevant clinical guidance. The factors that mean findings cannot be generalised from high resource settings to low and middle resource settings are multiple and will differ across interventions. Ethnicity, poverty, gender dynamics, pollution, temperature, climate, diet, access to healthcare, educational status, employment conditions are all examples of factors that might play a role in these differences. Improved understanding of the aetiopathogenesis of PTB is also necessary for defining an accurate model of risk prediction and would help in understanding what factors in local settings increase risk and facilitate the development of an accurate model of risk prediction.^51^

Two recent overviews of reviews^9 10^ also found that few interventions are effective in PTB prevention. The following interventions were identified in these reviews as showing positive or possible benefit: lifestyle and behavioural changes (including diet and exercise); nutritional supplements (including calcium, zinc and vitamin D supplementation); nutritional education; and screening for lower genital tract infections. Positive effects of secondary interventions were found for low dose aspirin among women at risk of pre-eclampsia; clindamycin for treatment of bacterial vaginosis; treatment of vaginal candidiasis; progesterone in women with prior spontaneous PTB and in those with short mid-trimester cervical length; L-arginine in women at risk for pre-eclampsia; levothyroxine among women with thyroid disease; calcium supplementation in women at risk of hypertensive disorders; smoking cessation; cervical length screening in women with history of PTB with placement of cerclage in those with short cervix; cervical pessary in singleton gestations with short cervix; and treatment of periodontal disease. Our review findings were in concordance, although, in addition, we identified screening and antibiotic treatment for syphilis, and positive effects of fish oil supplements. In most instances the trials were small and authors recommended larger well-designed randomised controlled trials (RCTs). The lack of consistency across review findings for interventions also merits more exploration. Compromised methodological rigour can inflate trial findings by 30%–50%.^52 53^ Some of the differences in our review findings reflect some differences in the included reviews.

The interventions identified in this review, and those of Matei *et al*^9^ and Medley *et al*^10^ informing guideline development, clinical practice and policy decision making have been little tested in LMIC settings. In those interventions where there is more consistency in review findings such as cervical cerclage, there are no studies that have been conducted in low-income settings and over half of the reviews did not report or consider settings in their analyses.

This scoping review has shown that many authors of systematic reviews fail to use design and statistical approaches that adequately address contextual variations between the included source studies and imperfectly represent ‘real world’ conditions within the target context. While those reviews that sought to take into account LMIC contexts were unable to conduct the analyses due to a lack of data, they nonetheless were able to highlight the gaps in research, for example the lack of studies in vitamin D undertaken in Africa.^31^

The Preferred Reporting Items for Systematic reviews and Meta-Analyses (PRISMA) reporting standards reference ‘context’ in terms of the circumstances requiring the review itself, rather than referencing the contexts of studies included in the review.^54^ The PRISMA extension for Complex Interventions includes the elements of ‘time’ and ‘setting’.^55^ However, grouping LMIC data, or even LI data may still be too broad. Even within the categories of LIC there is considerable diversity that may impact on how an intervention works and within countries there may also be considerable diversity between the wealthiest and poorest groups. For example, the time taken to reach comprehensive emergency obstetric care facilities in low resource settings is often underestimated and for most women is likely to be 120 min of travel time.^56^ Context cannot be standardised, it will vary from review to review, as different interventions and different populations are considered. ‘Context’ and the factors that might influence the efficacy, uptake, acceptability, appropriateness, accessibility and availability of an intervention requires a good understanding of the aetiology and mechanisms by which risk factors interact with environmental, microbial, socio-political and health system variations across settings.^57^

It must be acknowledged that there are significant barriers to undertaking research in many settings across the globe. These include very practical challenges such as a lack of access to high-quality data and the challenges of estimating gestational age.^58^ Recent changes to global health funding arena include a very large proportion being spent on the pandemic as well as government reductions, for example, in the UK.^59^ These reductions in funding will undermine what has been a growth in research in LMIC settings and will impede efforts to address the imbalances highlighted in this scoping review.

A number of limitations exist in this scoping review. We have not sought to identify the setting of primary studies where this is not reported in the systematic review. We have also not limited our analysis to studies within the reviews that only contributed findings to the risk of PTB. Most reviews explored several maternal and infant outcomes. Therefore, in this scoping review, included primary studies may not have contained PTB outcome data. We limited our scoping review to exploring evidence within systematic reviews as these are key sources of evidence to inform guideline development and policy decision making. It is possible that further primary studies have been published but are not included in this analysis. Nevertheless, it gives an indication of the distribution of research being undertaken in the poorest regions of the world that address PTB.

## Conclusion

Only 4.5% of primary research to examine the effectiveness of interventions to reduce the risk of PTB is carried out in settings where the burden is greatest. No interventions which reduce the risk of PTB, judged to be supported by strong evidence, include studies undertaken in low resource settings. In the synthesis of studies, current methods often fail to address the contextual variation and consider the applicability of findings in low resource, high burden settings. This has implications for supporting policy making, and development of contextually relevant clinical guidelines. While methods can be undertaken to improve approaches to evidence synthesis, they cannot compensate for the lack of primary research in low resource settings. This is critical if global health inequalities are to be addressed and millennium development goals^60^ to reduce under-five mortality are to be achieved. Funding and supporting research in LMICs would have a threefold benefit; first, if the prevalence of the disease is higher it is easier to reach statistical significance for efficacy or inefficacy of each tested intervention. Second, it would address the knowledge gap highlighted in this review and finally—and most importantly—the implementation of effective interventions would have the potential for greater public health impact where the risks are greater, more prevalent and outcomes more severe.

## Supplementary Material

Reviewer comments

Author's manuscript

## Data Availability

Data are available upon reasonable request. All data extracted from the included reviews is available on request from the corresponding author.

## References

[1] WHO. Who: recommended definitions, terminology and format for statistical tables related to the perinatal period and use of a new certificate for cause of perinatal deaths. modifications recommended by FIGO as amended October 14, 1976. Acta Obstet Gynecol Scand 1977;56:247–53. 560099

[2] Liu L, Oza S, Hogan D, et al. Global, regional, and national causes of child mortality in 2000-13, with projections to inform post-2015 priorities: an updated systematic analysis. Lancet 2015;385:430–40. 10.1016/S0140-6736(14)61698-6 25280870

[3] Brydges CR, Landes JK, Reid CL, et al. Cognitive outcomes in children and adolescents born very preterm: a meta-analysis. Dev Med Child Neurol 2018;60:452–68. 10.1111/dmcn.13685 29453812

[4] Coathup V, Boyle E, Carson C, et al. Gestational age and hospital admissions during childhood: population based, record linkage study in England (TIGAR study). BMJ 2020;371:m4075. 10.1136/bmj.m4075 33239272 PMC7687266

[5] Howson CP, Kinney MV, McDougall L, et al. Born too soon: preterm birth matters. Reprod Health 2013;10:1–9. 10.1186/1742-4755-10-S1-S1 24625113 PMC3828581

[6] Chawanpaiboon S, Vogel JP, Moller A-B, et al. Global, regional, and national estimates of levels of preterm birth in 2014: a systematic review and modelling analysis. Lancet Glob Health 2019;7:e37–46. 10.1016/S2214-109X(18)30451-0 30389451 PMC6293055

[7] Barros FC, Bhutta ZA, Batra M, et al. Global report on preterm birth and stillbirth (3 of 7): evidence for effectiveness of interventions. BMC Pregnancy Childbirth 2010;10:1–36. 10.1186/1471-2393-10-S1-S3 20233384 PMC2841444

[8] Bhutta ZA, Das JK, Bahl R, et al. Can available interventions end preventable deaths in mothers, newborn babies, and stillbirths, and at what cost? Lancet 2014;384:347–70. 10.1016/S0140-6736(14)60792-3 24853604

[9] Matei A, Saccone G, Vogel JP, et al. Primary and secondary prevention of preterm birth: a review of systematic reviews and ongoing randomized controlled trials. Eur J Obstet Gynecol Reprod Biol 2019;236:224–39. 10.1016/j.ejogrb.2018.12.022 30772047

[10] Medley N, Vogel JP, Care A, et al. Interventions during pregnancy to prevent preterm birth: an overview of Cochrane systematic reviews. Cochrane Database Syst Rev 2018;11:CD012505. 10.1002/14651858.CD012505.pub2 30480756 PMC6516886

[11] Opiyo N, Stones W. Corticosteroids for preterm deliveries: missing evidence. Cochrane Database Syst Rev 2017;5:ED000121. 10.1002/14651858.ED000121 28497861 PMC10845872

[12] Egwuatu VE. Complications of cervical cerclage in Igbo women. J Natl Med Assoc 1986;78:245. 3712463 PMC2571254

[13] White H, Albers B, Gaarder M, et al. Guidance for producing a Campbell evidence and gap MAP. Campbell Syst Rev 2020;16:e1125. 10.1002/cl2.1125 PMC8356343 37016607

[14] Arksey H, O'Malley L. Scoping studies: towards a methodological framework. Int J Soc Res Methodol 2005;8:19–32. 10.1080/1364557032000119616

[15] Allen F, Gray R, Oakley L. Technical guide to the infant mortality evidence map: systematic reviews of interventions targeting major potentially modifiable risk factors for infant mortality, 2009.

[16] Sutton A, Campbell F. The ScHARR LMIC filter: adapting a low- and middle-income countries geographic search filter to identify studies on preterm birth prevention and management. Res Synth Methods 2022. 10.1002/jrsm.1552. [Epub ahead of print: 10 Feb 2022]. PMC9543249 35142432

[17] Bank TW. World bank country and lending groups, 2021. Available: https://datahelpdesk.worldbank.org/knowledgebase/articles/906519 [Accessed 18 Mar 2021].

[18] Pergialiotis V, Vlachos DG, Prodromidou A, et al. Double versus single cervical cerclage for the prevention of preterm births. J Matern Fetal Neonatal Med 2015;28:379–85. 10.3109/14767058.2014.921676 24803126

[19] Romero R, Conde-Agudelo A, El-Refaie W, et al. Vaginal progesterone decreases preterm birth and neonatal morbidity and mortality in women with a twin gestation and a short cervix: an updated meta-analysis of individual patient data. Ultrasound Obstet Gynecol 2017;49:303–14. 10.1002/uog.17397 28067007 PMC5396280

[20] Romero R, Conde-Agudelo A, Da Fonseca E, et al. Vaginal progesterone for preventing preterm birth and adverse perinatal outcomes in singleton gestations with a short cervix: a meta-analysis of individual patient data. Am J Obstet Gynecol 2018;218:161–80. 10.1016/j.ajog.2017.11.576 29157866 PMC5987201

[21] Gresham E, Bisquera A, Byles JE, et al. Effects of dietary interventions on pregnancy outcomes: a systematic review and meta-analysis. Matern Child Nutr 2016;12:5–23. 10.1111/mcn.12142 25048387 PMC6860081

[22] Ehsanipoor RM, Seligman NS, Saccone G, et al. Physical Examination-Indicated Cerclage: a systematic review and meta-analysis. Obstet Gynecol 2015;126:125–35. 10.1097/AOG.0000000000000850 26241265

[23] Smaill FM, Vazquez JC, Cochrane Pregnancy and Childbirth Group. Antibiotics for asymptomatic bacteriuria in pregnancy. Cochrane Database Syst Rev 2015;8:CD000490. 10.1002/14651858.CD000490.pub3 26252501

[24] Blencowe H, Cousens S, Kamb M, et al. Lives saved tool supplement detection and treatment of syphilis in pregnancy to reduce syphilis related stillbirths and neonatal mortality. BMC Public Health 2011;11 Suppl 3:S9. 10.1186/1471-2458-11-S3-S9 PMC3231915 21501460

[25] Roberts CL, Algert CS, Rickard KL, et al. Treatment of vaginal candidiasis for the prevention of preterm birth: a systematic review and meta-analysis. Syst Rev 2015;4:31. 10.1186/s13643-015-0018-2 25874659 PMC4373465

[26] Sangkomkamhang US, Lumbiganon P, Prasertcharoensuk W, et al. Antenatal lower genital tract infection screening and treatment programs for preventing preterm delivery. Cochrane Database Syst Rev 2015:CD006178. 10.1002/14651858.CD006178.pub3 25922860 PMC8498019

[27] Zhou S-S, Tao Y-H, Huang K, et al. Vitamin D and risk of preterm birth: up-to-date meta-analysis of randomized controlled trials and observational studies. J Obstet Gynaecol Res 2017;43:247–56. 10.1111/jog.13239 28150405

[28] Mbuagbaw L, Medley N, Darzi AJ, et al. Health system and community level interventions for improving antenatal care coverage and health outcomes. Cochrane Database Syst Rev 2015:CD010994. 10.1002/14651858.CD010994.pub2 26621223 PMC4676908

[29] Girard AW, Olude O. Nutrition education and counselling provided during pregnancy: effects on maternal, neonatal and child health outcomes. Paediatr Perinat Epidemiol 2012;26 Suppl 1:191–204. 10.1111/j.1365-3016.2012.01278.x 22742611

[30] Ota E, Hori H, Mori R, et al. Antenatal dietary education and supplementation to increase energy and protein intake. Cochrane Database Syst Rev 2015;6:CD000032. 10.1002/14651858.CD000032.pub3 PMC12634316 26031211

[31] Roth DE, Leung M, Mesfin E, et al. Vitamin D supplementation during pregnancy: state of the evidence from a systematic review of randomised trials. BMJ 2017;359:j5237. 10.1136/bmj.j5237 29187358 PMC5706533

[32] Chaffee BW, King JC. Effect of zinc supplementation on pregnancy and infant outcomes: a systematic review. Paediatr Perinat Epidemiol 2012;26 Suppl 1:118–37. 10.1111/j.1365-3016.2012.01289.x 22742606 PMC3787719

[33] Radeva-Petrova D, Kayentao K, ter Kuile FO, et al. Drugs for preventing malaria in pregnant women in endemic areas: any drug regimen versus placebo or no treatment. Cochrane Database Syst Rev 2014;9:CD000169. 10.1002/14651858.CD000169.pub3 PMC4498495 25300703

[34] Ota E, Mori R, Middleton P, et al. Zinc supplementation for improving pregnancy and infant outcome. Cochrane Database Syst Rev 2015;31. 10.1002/14651858.CD000230.pub5 PMC7043363 25927101

[35] Chamberlain C, O'Mara-Eves A, Oliver S, et al. Psychosocial interventions for supporting women to stop smoking in pregnancy. Cochrane Database Syst Rev 2013;10:CD001055. 10.1002/14651858.CD001055.pub4 PMC4022453 24154953

[36] Palacios C, De-Regil LM, Lombardo LK, et al. Vitamin D supplementation during pregnancy: updated meta-analysis on maternal outcomes. J Steroid Biochem Mol Biol 2016;164:148–55. 10.1016/j.jsbmb.2016.02.008 26877200 PMC5357731

[37] McCauley ME, van den Broek N, Dou L, et al. Vitamin A supplementation during pregnancy for maternal and newborn outcomes. Cochrane Database Syst Rev 2015;10:CD008666. 10.1002/14651858.CD008666.pub3 PMC7173731 26503498

[38] Rumbold A, Ota E, Hori H, et al. Vitamin E supplementation in pregnancy. Cochrane Database Syst Rev 2015:CD004069. 10.1002/14651858.CD004069.pub3 26343254 PMC8406700

[39] Rumbold A, Ota E, Nagata C, et al. Vitamin C supplementation in pregnancy. Cochrane Database Syst Rev 2015:CD004072. 10.1002/14651858.CD004072.pub3 26415762 PMC9039972

[40] Peña-Rosas JP, De-Regil LM, Garcia-Casal MN, et al. Daily oral iron supplementation during pregnancy. Cochrane Database Syst Rev 2015;2015. 10.1002/14651858.CD004736.pub5 PMC8918165 26198451

[41] Hofmeyr GJ, Lawrie TA, Atallah Álvaro N, et al. Calcium supplementation during pregnancy for preventing hypertensive disorders and related problems. Cochrane Database Syst Rev 2018;10:CD001059. 10.1002/14651858.CD001059.pub5 30277579 PMC6517256

[42] Hofmeyr GJ, Manyame S, Medley N, et al. Calcium supplementation commencing before or early in pregnancy, for preventing hypertensive disorders of pregnancy. Cochrane Database Syst Rev 2019;393. 10.1002/14651858.CD011192.pub3 PMC6745517 31523806

[43] Keats EC, Haider BA, Tam E, et al. Multiple-micronutrient supplementation for women during pregnancy. Cochrane Database Syst Rev 2019;145. 10.1002/14651858.CD004905.pub6 PMC6418471 30873598

[44] Smith ER, Shankar AH, Wu LS-F, et al. Modifiers of the effect of maternal multiple micronutrient supplementation on stillbirth, birth outcomes, and infant mortality: a meta-analysis of individual patient data from 17 randomised trials in low-income and middle-income countries. Lancet Glob Health 2017;5:e1090–100. 10.1016/S2214-109X(17)30371-6 29025632

[45] Berghella V, Ciardulli A, Rust OA, et al. Cerclage for sonographic short cervix in singleton gestations without prior spontaneous preterm birth: systematic review and meta-analysis of randomized controlled trials using individual patient-level data. Ultrasound Obstet Gynecol 2017;50:569–77. 10.1002/uog.17457 28295722

[46] Bellows BW, Conlon CM, Higgs ES, et al. A taxonomy and results from a comprehensive review of 28 maternal health voucher programmes. J Health Popul Nutr 2013;31:S106.24992806

[47] Atal I, Trinquart L, Ravaud P, et al. A mapping of 115,000 randomized trials revealed a mismatch between research effort and health needs in non-high-income regions. J Clin Epidemiol 2018;98:123–32. 10.1016/j.jclinepi.2018.01.006 29360559

[48] Dab W. Commentary on sphere (strengthening public health research in Europe) literature reviews. Eur J Public Health 2007;17 Suppl 1:8–9. 10.1093/eurpub/ckm060 17666414

[49] Shepherd V. Research involving adults lacking capacity to consent: the impact of research regulation on ‘evidence biased’ medicine. BMC Med Ethics 2016;17:1–8. 10.1186/s12910-016-0138-9 27609355 PMC5016956

[50] World Health Organization. Born too soon: the global action report on preterm birth, 2012.

[51] Della Rosa PA, Miglioli C, Caglioni M, et al. A hierarchical procedure to select intrauterine and extrauterine factors for methodological validation of preterm birth risk estimation. BMC Pregnancy Childbirth 2021;21:1–17. 10.1186/s12884-021-03654-3 33863296 PMC8052693

[52] Linde K, Scholz M, Ramirez G, et al. Impact of study quality on outcome in placebo-controlled trials of homeopathy. J Clin Epidemiol 1999;52:631–6. 10.1016/s0895-4356(99)00048-7 10391656

[53] Schulz KF, Chalmers I, Hayes RJ, et al. Empirical evidence of bias. dimensions of methodological quality associated with estimates of treatment effects in controlled trials. JAMA 1995;273:408–12. 10.1001/jama.273.5.408 7823387

[54] Moher D, Liberati A, Tetzlaff J, et al. Preferred reporting items for systematic reviews and meta-analyses: the PRISMA statement. PLoS Med 2009;6:e1000097. 10.1371/journal.pmed.1000097 19621072 PMC2707599

[55] Guise J-M, Butler ME, Chang C, et al. AHRQ series on complex intervention systematic reviews-paper 6: PRISMA-CI extension statement and checklist. J Clin Epidemiol 2017;90:43–50. 10.1016/j.jclinepi.2017.06.016 28720516

[56] Banke-Thomas A, Wong KLM, Ayomoh FI, et al. "In cities, it's not far, but it takes long": comparing estimated and replicated travel times to reach life-saving obstetric care in Lagos, Nigeria. BMJ Glob Health 2021;6:e004318. 10.1136/bmjgh-2020-004318 PMC7839900 33495286

[57] Rogers L, De Brún A, McAuliffe E. Defining and assessing context in healthcare implementation studies: a systematic review. BMC Health Serv Res 2020;20:591. 10.1186/s12913-020-05212-7 32600396 PMC7322847

[58] Vogel JP, Chawanpaiboon S, Moller A-B, et al. The global epidemiology of preterm birth. Best Pract Res Clin Obstet Gynaecol 2018;52:3–12. 10.1016/j.bpobgyn.2018.04.003 29779863

[59] C S. UKRI official development assistance letter 11 March 2021, 2021. Available: https://www.ukri.org/our-work/ukri-oda-letter-11-march-2021/ [Accessed 23 Mar 2021].

[60] World Health Organization. Millennium development goals, 2008.

[61] Allen J, Gamble J, Stapleton H, et al. Does the way maternity care is provided affect maternal and neonatal outcomes for young women? A review of the research literature. Women Birth 2012;25:54–63. 10.1016/j.wombi.2011.03.002 21493173

[62] Catling CJ, Medley N, Foureur M. Group versus conventional antenatal care for women. Cochrane Database Syst Rev 2015;2:CD007622. 10.1002/14651858.CD007622.pub3 PMC6465187 25922865

[63] Dodd JM, Dowswell T, Crowther CA. Specialised antenatal clinics for women with a multiple pregnancy for improving maternal and infant outcomes. Cochrane Database Syst Rev 2015:CD005300. 10.1002/14651858.CD005300.pub4 26545291 PMC8536469

[64] Dowswell T, Carroli G, Duley L, et al. Alternative versus standard packages of antenatal care for low-risk pregnancy. Cochrane Database Syst Rev 2015;7:CD000934. 10.1002/14651858.CD000934.pub3 PMC7061257 26184394

[65] Dowswell T, Middleton P, Weeks A. Antenatal day care units versus hospital admission for women with complicated pregnancy. Cochrane Database Syst Rev 2009:CD001803. 10.1002/14651858.CD001803.pub2 19821282 PMC4171387

[66] Fernandez Turienzo C, Sandall J, Peacock JL. Models of antenatal care to reduce and prevent preterm birth: a systematic review and meta-analysis. BMJ Open 2016;6:e009044. 10.1136/bmjopen-2015-009044 PMC4716175 26758257

[67] Lathrop B. A systematic review comparing group prenatal care to traditional prenatal care. Nurs Womens Health 2013;17:118–30. 10.1111/1751-486X.12020 23594324

[68] Malouf R, Redshaw M. Specialist antenatal clinics for women at high risk of preterm birth: a systematic review of qualitative and quantitative research. BMC Pregnancy Childbirth 2017;17:51. 10.1186/s12884-017-1232-9 28148230 PMC5288877

[69] Ruiz-Mirazo E, Lopez-Yarto M, McDonald SD. Group prenatal care versus individual prenatal care: a systematic review and meta-analyses. J Obstet Gynaecol Can 2012;34:223–9. 10.1016/S1701-2163(16)35182-9 22385664

[70] Sheeder J, Weber Yorga K, Kabir-Greher K. A review of prenatal group care literature: the need for a structured theoretical framework and systematic evaluation. Matern Child Health J 2012;16:177–87. 10.1007/s10995-010-0709-1 21088988

[71] Whitworth M, Quenby S, Cockerill RO, et al. Specialised antenatal clinics for women with a pregnancy at high risk of preterm birth (excluding multiple pregnancy) to improve maternal and infant outcomes. Cochrane Database Syst Rev 2011;9:CD006760. 10.1002/14651858.CD006760.pub2 PMC4084921 21901705

[72] Sandall J, Soltani H, Gates S, et al. Midwife-led continuity models versus other models of care for childbearing women. Cochrane Database Syst Rev 2013:CD004667. 10.1002/14651858.CD004667.pub3 23963739

[73] Coleman T, Chamberlain C, Davey M-A, et al. Pharmacological interventions for promoting smoking cessation during pregnancy. Cochrane Database Syst Rev 2012;9:CD010078. 10.1002/14651858.CD010078 22972148

[74] Dodd JM, Grivell RM, Crowther CA, et al. Antenatal interventions for overweight or obese pregnant women: a systematic review of randomised trials. BJOG 2010;117:1316–26. 10.1111/j.1471-0528.2010.02540.x 20353459

[75] Muktabhant B, Lawrie TA, Lumbiganon P, et al. Diet or exercise, or both, for preventing excessive weight gain in pregnancy. Cochrane Database Syst Rev 2015;6:CD007145. 10.1002/14651858.CD007145.pub3 PMC9428894 26068707

[76] Shepherd E, Gomersall JC, Tieu J, et al. Combined diet and exercise interventions for preventing gestational diabetes mellitus. Cochrane Database Syst Rev 2017;2017. 10.1002/14651858.CD010443.pub3 PMC6485974 29129039

[77] Tanentsapf I, Heitmann BL, Adegboye ARA. Systematic review of clinical trials on dietary interventions to prevent excessive weight gain during pregnancy among normal weight, overweight and obese women. BMC Pregnancy Childbirth 2011;11:81. 10.1186/1471-2393-11-81 22029725 PMC3215955

[78] Thangaratinam S, Rogozińska E, Jolly K, et al. Interventions to reduce or prevent obesity in pregnant women: a systematic review. Health Technol Assess 2012;16. 10.3310/hta16310 PMC4781281 22814301

[79] Bi WG, Nuyt AM, Weiler H, et al. Association between vitamin D supplementation during pregnancy and offspring growth, morbidity, and mortality: a systematic review and meta-analysis. JAMA Pediatr 2018;172:635–45. 10.1001/jamapediatrics.2018.0302 29813153 PMC6137512

[80] Pérez-López FR, Pasupuleti V, Mezones-Holguin E, et al. Effect of vitamin D supplementation during pregnancy on maternal and neonatal outcomes: a systematic review and meta-analysis of randomized controlled trials. Fertil Steril 2015;103:1278-88.e4. 10.1016/j.fertnstert.2015.02.019 25813278

[81] Thorne-Lyman A, Fawzi WW. Vitamin D during pregnancy and maternal, neonatal and infant health outcomes: a systematic review and meta-analysis. Paediatr Perinat Epidemiol 2012;26 Suppl 1:75–90. 10.1111/j.1365-3016.2012.01283.x 22742603 PMC3843348

[82] Thorne-Lyman AL, Fawzi WW. Vitamin A and carotenoids during pregnancy and maternal, neonatal and infant health outcomes: a systematic review and meta-analysis. Paediatr Perinat Epidemiol 2012;26 Suppl 1:36–54. 10.1111/j.1365-3016.2012.01284.x 22742601 PMC3843354

[83] Rahimi R, Nikfar S, Rezaie A, et al. A meta-analysis on the efficacy and safety of combined vitamin C and E supplementation in preeclamptic women. Hypertens Pregnancy 2009;28:417–34. 10.3109/10641950802629667 19843004

[84] Lassi ZS, Salam RA, Haider BA, et al. Folic acid supplementation during pregnancy for maternal health and pregnancy outcomes. Cochrane Database Syst Rev 2013:CD006896. 10.1002/14651858.CD006896.pub2 23543547 PMC10069458

[85] Mantovani E, Filippini F, Bortolus R, et al. Folic acid supplementation and preterm birth: results from observational studies. Biomed Res Int 2014;2014:481914. 10.1155/2014/481914 24724083 PMC3958780

[86] Peña-Rosas JP, De-Regil LM, Dowswell T, et al. Intermittent oral iron supplementation during pregnancy. Cochrane Database Syst Rev 2012;7:CD009997. 10.1002/14651858.CD009997 PMC4053594 22786531

[87] Peña-Rosas JP, Viteri FE. Effects and safety of preventive oral iron or iron+folic acid supplementation for women during pregnancy. Cochrane Database Syst Rev 2009:CD004736. 10.1002/14651858.CD004736.pub3 19821332

[88] Saccone G, Berghella V. Folic acid supplementation in pregnancy to prevent preterm birth: a systematic review and meta-analysis of randomized controlled trials. Eur J Obstet Gynecol Reprod Biol 2016;199:76–81. 10.1016/j.ejogrb.2016.01.042 26901401

[89] Zhang Q, Wang Y, Xin X, et al. Effect of folic acid supplementation on preterm delivery and small for gestational age births: a systematic review and meta-analysis. Reprod Toxicol 2017;67:35–41. 10.1016/j.reprotox.2016.11.012 27856370

[90] Imdad A, Bhutta ZA. Routine iron/folate supplementation during pregnancy: effect on maternal anaemia and birth outcomes. Paediatr Perinat Epidemiol 2012;26 Suppl 1:168–77. 10.1111/j.1365-3016.2012.01312.x 22742609

[91] Chen B, Ji X, Zhang L, et al. Fish oil supplementation improves pregnancy outcomes and size of the newborn: a meta-analysis of 21 randomized controlled trials. J Matern Fetal Neonatal Med 2016;29:2017–27. 10.3109/14767058.2015.1072163 27012494

[92] Kar S, Wong M, Rogozinska E, et al. Effects of omega-3 fatty acids in prevention of early preterm delivery: a systematic review and meta-analysis of randomized studies. Eur J Obstet Gynecol Reprod Biol 2016;198:40–6. 10.1016/j.ejogrb.2015.11.033 26773247

[93] Saccone G, Berghella V. Omega-3 supplementation to prevent recurrent preterm birth: a systematic review and metaanalysis of randomized controlled trials. Am J Obstet Gynecol 2015;213:135–40. 10.1016/j.ajog.2015.03.013 25757636

[94] Salvig JD, Lamont RF. Evidence regarding an effect of marine n-3 fatty acids on preterm birth: a systematic review and meta-analysis. Acta Obstet Gynecol Scand 2011;90:825–38. 10.1111/j.1600-0412.2011.01171.x 21535434

[95] Saccone G, Saccone I, Berghella V. Omega-3 long-chain polyunsaturated fatty acids and fish oil supplementation during pregnancy: which evidence? J Matern Fetal Neonatal Med 2016;29:2389–97. 10.3109/14767058.2015.1086742 26382010

[96] Mori R, Ota E, Middleton P. Zinc supplementation for improving pregnancy and infant outcome. Cochrane Database Syst Rev 2012;3:CD000230. 10.1002/14651858.CD000230.pub4 22786472

[97] Harding KB, Peña-Rosas JP, Webster AC, et al. Iodine supplementation for women during the preconception, pregnancy and postpartum period. Cochrane Database Syst Rev 2017;3:CD011761. 10.1002/14651858.CD011761.pub2 28260263 PMC6464647

[98] Fall CHD, Fisher DJ, Osmond C, et al. Multiple micronutrient supplementation during pregnancy in low-income countries: a meta-analysis of effects on birth size and length of gestation. Food Nutr Bull 2009;30:S533–46. 10.1177/15648265090304S408 20120795 PMC3541502

[99] Schwendicke F, Karimbux N, Allareddy V, et al. Periodontal treatment for preventing adverse pregnancy outcomes: a meta- and trial sequential analysis. PLoS One 2015;10:e0129060. 10.1371/journal.pone.0129060 26035835 PMC4452791

[100] Corbella S, Del Fabbro M, Taschieri S, et al. Periodontal disease and adverse pregnancy outcomes: a systematic review. Italian Oral Surgery 2012;11:132–46. 10.1016/j.ios.2011.04.002

[101] Fogacci MF, Vettore MV, Thomé Leão AT, Leao AT. The effect of periodontal therapy on preterm low birth weight: a meta-analysis. Obstet Gynecol 2011;117:153–65. 10.1097/AOG.0b013e3181fdebc0 21173658

[102] George A, Shamim S, Johnson M, et al. Periodontal treatment during pregnancy and birth outcomes: a meta-analysis of randomised trials. Int J Evid Based Healthc 2011;9:122–47. 10.1111/j.1744-1609.2011.00210.x 21599842

[103] Pimentel Lopes De Oliveira GJ, Amaral Fontanari L, Chaves De Souza JA, et al. Effect of periodontal treatment on the incidence of preterm delivery: a systematic review. Minerva Stomatol 2010;59:543–50. 21048546

[104] Polyzos NP, Polyzos IP, Zavos A, et al. Obstetric outcomes after treatment of periodontal disease during pregnancy: systematic review and meta-analysis. BMJ 2010;341:c7017. 10.1136/bmj.c7017 21190966 PMC3011371

[105] Rosa MIda, Pires PDS, Medeiros LR, et al. Periodontal disease treatment and risk of preterm birth: a systematic review and meta-analysis. Cad Saude Publica 2012;28:1823–33. 10.1590/s0102-311x2012001000002 23090163

[106] Shah M, Muley A, Muley P. Effect of nonsurgical periodontal therapy during gestation period on adverse pregnancy outcome: a systematic review. J Matern Fetal Neonatal Med 2013;26:1691–5. 10.3109/14767058.2013.799662 23617740

[107] Uppal A, Uppal S, Pinto A, et al. The effectiveness of periodontal disease treatment during pregnancy in reducing the risk of experiencing preterm birth and low birth weight: a meta-analysis. J Am Dent Assoc 2010;141:1423–34. 10.14219/jada.archive.2010.0104 21119126

[108] Kim AJ, Lo AJ, Pullin DA, et al. Scaling and root planing treatment for periodontitis to reduce preterm birth and low birth weight: a systematic review and meta-analysis of randomized controlled trials. J Periodontol 2012;83:1508–19. 10.1902/jop.2012.110636 22376207

[109] da Silva HEC, Stefani CM, de Santos Melo N, et al. Effect of intra-pregnancy nonsurgical periodontal therapy on inflammatory biomarkers and adverse pregnancy outcomes: a systematic review with meta-analysis. Syst Rev 2017;6:197. 10.1186/s13643-017-0587-3 29017560 PMC5635531

[110] Iheozor-Ejiofor Z, Middleton P, Esposito M, et al. Treating periodontal disease for preventing adverse birth outcomes in pregnant women. Cochrane Database Syst Rev 2017;6:CD005297. 10.1002/14651858.CD005297.pub3 28605006 PMC6481493

[111] Guinto VT, De Guia B, Festin MR, et al. Different antibiotic regimens for treating asymptomatic bacteriuria in pregnancy. Cochrane Database Syst Rev 2010:CD007855. 10.1002/14651858.CD007855.pub2 20824868 PMC4033758

[112] Widmer M, Lopez I, Gülmezoglu AM, et al. Duration of treatment for asymptomatic bacteriuria during pregnancy. Cochrane Database Syst Rev 2015;11:CD000491. 10.1002/14651858.CD000491.pub3 PMC7043273 26560337

[113] Angelescu K, Nussbaumer-Streit B, Sieben W, et al. Benefits and harms of screening for and treatment of asymptomatic bacteriuria in pregnancy: a systematic review. BMC Pregnancy Childbirth 2016;16:336. 10.1186/s12884-016-1128-0 27806709 PMC5093995

[114] Fell DB, Platt RW, Lanes A, et al. Fetal death and preterm birth associated with maternal influenza vaccination: systematic review. BJOG 2015;122:17–26. 10.1111/1471-0528.12977 25040307

[115] Zhang C, Wang X, Liu D, et al. A systematic review and meta-analysis of fetal outcomes following the administration of influenza A/H1N1 vaccination during pregnancy. Int J Gynaecol Obstet 2018;141:141–50. 10.1002/ijgo.12394 29149524

[116] Schneeberger C, Geerlings SE, Middleton P, et al. Interventions for preventing recurrent urinary tract infection during pregnancy. Cochrane Database Syst Rev 2015:CD009279. 10.1002/14651858.CD009279.pub3 26221993 PMC6457953

[117] Vazquez JC, Abalos E. Treatments for symptomatic urinary tract infections during pregnancy. Cochrane Database Syst Rev 2011;1:CD002256. 10.1002/14651858.CD002256.pub2 PMC7144687 21249652

[118] Flenady V, Hawley G, Stock OM, et al. Prophylactic antibiotics for inhibiting preterm labour with intact membranes. Cochrane Database Syst Rev 2013;12:CD000246. 10.1002/14651858.CD000246.pub2 PMC11975194 24307518

[119] Thinkhamrop J, Hofmeyr GJ, Adetoro O. Antibiotic prophylaxis during the second and third trimester to reduce adverse pregnancy outcomes and morbidity. Cochrane Database of Systematic Reviews 2015:CD002250. 10.1002/14651858.CD002250.pub2 25621770

[120] Manyando C, Kayentao K, D'Alessandro U, et al. A systematic review of the safety and efficacy of artemether-lumefantrine against uncomplicated Plasmodium falciparum malaria during pregnancy. Malar J 2012;11:141. 10.1186/1475-2875-11-141 22548983 PMC3405476

[121] Gamble C, Ekwaru PJ, Garner P, et al. Insecticide-Treated nets for the prevention of malaria in pregnancy: a systematic review of randomised controlled trials. PLoS Med 2007;4:e107. 10.1371/journal.pmed.0040107 17388668 PMC1831739

[122] Alfirevic Z, Stampalija T, Medley N. Cervical stitch (cerclage) for preventing preterm birth in singleton pregnancy. Cochrane Database Syst Rev 2017;6:CD008991. 10.1002/14651858.CD008991.pub3 28586127 PMC6481522

[123] Berghella V, Keeler SM, To MS, et al. Effectiveness of cerclage according to severity of cervical length shortening: a meta-analysis. Ultrasound Obstet Gynecol 2010;35:468–73. 10.1002/uog.7547 20052661

[124] Berghella V, Rafael TJ, Szychowski JM, et al. Cerclage for short cervix on ultrasonography in women with singleton gestations and previous preterm birth: a meta-analysis. Obstet Gynecol 2011;117:663–71. 10.1097/AOG.0b013e31820ca847 21446209

[125] Defranco EA, Valent AM, Newman T, et al. Adjunctive therapies to cerclage for the prevention of preterm birth: a systematic review. Obstet Gynecol Int 2013;2013:528158. 10.1155/2013/528158 23606847 PMC3625609

[126] Moawad GN, Tyan P, Bracke T, et al. Systematic review of transabdominal Cerclage placed via laparoscopy for the prevention of preterm birth. J Minim Invasive Gynecol 2018;25:277–86. 10.1016/j.jmig.2017.07.021 28797657

[127] Namouz S, Porat S, Okun N. Emergency cerclage: literature review (provisional Abstract). Obstetrical and Gynecological Survey 2013;68:379–88. 23624963 10.1097/OGX.0b013e31828737c7

[128] Rafael TJ, Berghella V, Alfirevic Z. Cervical stitch (cerclage) for preventing preterm birth in multiple pregnancy. Cochrane Database Syst Rev 2014;9:CD009166. 10.1002/14651858.CD009166.pub2 PMC10629495 25208049

[129] Saccone G, Rust O, Althuisius S, et al. Cerclage for short cervix in twin pregnancies: systematic review and meta-analysis of randomized trials using individual patient-level data. Acta Obstet Gynecol Scand 2015;94:352–8. 10.1111/aogs.12600 25644964

[130] Smith J, DeFranco EA. Tocolytics used as adjunctive therapy at the time of cerclage placement: a systematic review. J Perinatol 2015;35:561–5. 10.1038/jp.2015.38 25905689

[131] Zeybek B, Hill A, Menderes G, et al. Robot-Assisted abdominal Cerclage during pregnancy. JSLS 2016;20. 10.4293/JSLS.2016.00072 PMC5118107 27904309

[132] Liu X, Luo X, Xiao X. Cervical cerclage for preventing preterm birth in twin pregnancies. A systematic review and meta-analysis (provisional Abstract). Database of Abstracts of Reviews of Effects 2013;4:632–8. 23756929

[133] Conde-Agudelo A, Romero R, Da Fonseca E, et al. Vaginal progesterone is as effective as cervical cerclage to prevent preterm birth in women with a singleton gestation, previous spontaneous preterm birth, and a short cervix: updated indirect comparison meta-analysis. Am J Obstet Gynecol 2018;219:10–25. 10.1016/j.ajog.2018.03.028 29630885 PMC6449041

[134] Conde-Agudelo A, Romero R, Nicolaides K, et al. Vaginal progesterone vs. cervical cerclage for the prevention of preterm birth in women with a sonographic short cervix, previous preterm birth, and singleton gestation: a systematic review and indirect comparison metaanalysis. Am J Obstet Gynecol 2013;208:42.e1-42.e18. 10.1016/j.ajog.2012.10.877 23157855 PMC3529767

[135] Jarde A, Lutsiv O, Park CK, et al. Preterm birth prevention in twin pregnancies with progesterone, pessary, or cerclage: a systematic review and meta-analysis. BJOG 2017;124:1163–73. 10.1111/1471-0528.14513 28176485

[136] Jarde A, Lutsiv O, Park CK, et al. Effectiveness of progesterone, cerclage and pessary for preventing preterm birth in singleton pregnancies: a systematic review and network meta-analysis. BJOG 2017;124:1176–89. 10.1111/1471-0528.14624 28276151

[137] Crowther CA, Han S. Hospitalisation and bed rest for multiple pregnancy. Cochrane Database Syst Rev 2010;7:CD000110. 10.1002/14651858.CD000110.pub2 PMC7051031 20614420

[138] Maloni JA. Antepartum bed rest for pregnancy complications: efficacy and safety for preventing preterm birth. Biol Res Nurs 2010;12:106–24. 10.1177/1099800410375978 20798159

[139] Sosa CG, Althabe F, Belizán JM, et al. Bed rest in singleton pregnancies for preventing preterm birth. Cochrane Database Syst Rev 2015;3:CD003581. 10.1002/14651858.CD003581.pub3 PMC7144825 25821121

[140] Jin Z, Chen L, Qiao D. Cervical pessary for preventing preterm birth: a meta-analysis. J Matern Fetal Neonatal Med 2017. 10.1080/14767058.2017.1401998 29103351

[141] Liem SMS, van Pampus MG, Mol BWJ, et al. Cervical pessaries for the prevention of preterm birth: a systematic review. Obstet Gynecol Int 2013;2013:1–10. 10.1155/2013/576723 PMC3628502 23606848

[142] Saccone G, Ciardulli A, Xodo S, et al. Cervical pessary for preventing preterm birth in twin pregnancies with short cervical length: a systematic review and meta-analysis. J Matern Fetal Neonatal Med 2017;30:2918–25. 10.1080/14767058.2016.1268595 27915496

[143] Saccone G, Ciardulli A, Xodo S, et al. Cervical pessary for preventing preterm birth in singleton pregnancies with short cervical length: a systematic review and meta-analysis. Obstet Gynecol Surv 2018;73:13–14. 10.1097/01.ogx.0000528014.01777.83 28398701

[144] Thangatorai R, Lim FC, Nalliah S. Cervical pessary in the prevention of preterm births in multiple pregnancies with a short cervix: PRISMA compliant systematic review and meta-analysis. J Matern Fetal Neonatal Med 2018;31:1638–45. 10.1080/14767058.2017.1319930 28412851

[145] Zheng L, Dong J, Dai Y, et al. Cervical pessaries for the prevention of preterm birth: a systematic review and meta-analysis. J Matern Fetal Neonatal Med 2019;32:1654–63. 10.1080/14767058.2017.1414795 29212400

[146] Romero R, Nicolaides KH, Conde-Agudelo A, et al. Vaginal progesterone decreases preterm birth ≤ 34 weeks of gestation in women with a singleton pregnancy and a short cervix: an updated meta-analysis including data from the OPPTIMUM study. Ultrasound Obstet Gynecol 2016;48:308–17. 10.1002/uog.15953 27444208 PMC5053235

[147] Saccone G, Khalifeh A, Elimian A, et al. Vaginal progesterone vs intramuscular 17α-hydroxyprogesterone caproate for prevention of recurrent spontaneous preterm birth in singleton gestations: systematic review and meta-analysis of randomized controlled trials. Ultrasound Obstet Gynecol 2017;49:315–21. 10.1002/uog.17245 27546354

[148] Samuel M, Chong YS. Progestational agents for treating threatened or established preterm labour. Cochrane Database of Systematic Reviews 2010:CD006770. 20091604 10.1002/14651858.CD006770.pub2

[149] Suhag A, Saccone G, Berghella V. Vaginal progesterone for maintenance tocolysis: a systematic review and metaanalysis of randomized trials. Am J Obstet Gynecol 2015;213:479–87. 10.1016/j.ajog.2015.03.031 25797233

[150] Dodd JM, Grivell RM, OBrien CM, et al. Prenatal administration of progestogens for preventing spontaneous preterm birth in women with a multiple pregnancy. Cochrane Database Syst Rev 2019;2019. 10.1002/14651858.CD012024.pub3. [Epub ahead of print: 20 11 2019]. PMC6864412 31745984

[151] Sotiriadis A, Papatheodorou S, Makrydimas G. Perinatal outcome in women treated with progesterone for the prevention of preterm birth: a meta-analysis. Ultrasound Obstet Gynecol 2012;40:257–66. 10.1002/uog.11178 22611023

[152] Lim CED, Ho KKW, Cheng NCL, et al. Combined oestrogen and progesterone for preventing miscarriage. Cochrane Database Syst Rev 2013;9:CD009278. 10.1002/14651858.CD009278.pub2 PMC7389506 24068368

[153] Norman JE, Mackenzie F, Owen P, et al. Progesterone for the prevention of preterm birth in twin pregnancy (STOPPIT): a randomised, double-blind, placebo-controlled study and meta-analysis. Lancet 2009;373:646–8. 10.1016/S0140-6736(09)60947-8 19523680

[154] Likis FE, Edwards DRV, Andrews JC, et al. Progestogens for preterm birth prevention: a systematic review and meta-analysis. Obstet Gynecol 2012;120:897–907. 10.1097/AOG.0b013e3182699a15 22955308

[155] Palacio M, Ronzoni S, Sánchez-Ramos L, et al. Progestogens as maintenance treatment in arrested preterm labor: a systematic review and meta-analysis. Obstet Gynecol 2016;128:989–1000. 10.1097/AOG.0000000000001676 27741193

[156] Prior M, Hibberd R, Asemota N, et al. Inadvertent P-hacking among trials and systematic reviews of the effect of progestogens in pregnancy? A systematic review and meta-analysis. BJOG 2017;124:1008–15. 10.1111/1471-0528.14506 28318099

[157] Rode L, Langhoff-Roos J, Andersson C, et al. Systematic review of progesterone for the prevention of preterm birth in singleton pregnancies. Acta Obstet Gynecol Scand 2009;88:1180–9. 10.3109/00016340903280982 19900136

[158] Schmouder VM, Prescott GM, Franco A, et al. The rebirth of progesterone in the prevention of preterm labor. Ann Pharmacother 2013;47:527–36. 10.1345/aph.1R281 23535817

[159] Velez Edwards DR, Likis FE, Andrews JC, et al. Progestogens for preterm birth prevention: a systematic review and meta-analysis by drug route. Arch Gynecol Obstet 2013;287:1059–66. 10.1007/s00404-013-2789-9 23532387

[160] Chawanpaiboon S, Laopaiboon M, Lumbiganon P, et al. Terbutaline pump maintenance therapy after threatened preterm labour for reducing adverse neonatal outcomes. Cochrane Database Syst Rev 2014;3:CD010800. 10.1002/14651858.CD010800.pub2 PMC11193541 24659357

[161] Crowther CA, Brown J, McKinlay CJD, et al. Magnesium sulphate for preventing preterm birth in threatened preterm labour. Cochrane Database Syst Rev 2014;8:CD001060. 10.1002/14651858.CD001060.pub2 PMC10838393 25126773

[162] Gaudet LM, Singh K, Weeks L, et al. Effectiveness of terbutaline pump for the prevention of preterm birth. A systematic review and meta-analysis. PLoS One 2012;7:e31679. 10.1371/journal.pone.0031679 22363704 PMC3283660

[163] Haas DM, Caldwell DM, Kirkpatrick P, et al. Tocolytic therapy for preterm delivery: systematic review and network meta-analysis. BMJ 2012;345:e6226. 10.1136/bmj.e6226 23048010 PMC4688428

[164] McNamara HC, Crowther CA, Brown J. Different treatment regimens of magnesium sulphate for tocolysis in women in preterm labour. Cochrane Database Syst Rev 2015;12:CD011200. 10.1002/14651858.CD011200.pub2 PMC8697562 26662716

[165] Vogel JP, Nardin JM, Dowswell T, et al. Combination of tocolytic agents for inhibiting preterm labour. Cochrane Database Syst Rev 2014;7:CD006169. 10.1002/14651858.CD006169.pub2 PMC10657484 25010869

[166] Dodd JM, Crowther CA, Middleton P. Oral betamimetics for maintenance therapy after threatened preterm labour. Cochrane Database Syst Rev 2012;12:CD003927. 10.1002/14651858.CD003927.pub3 23235600 PMC10964130

[167] Flenady V, Reinebrant HE, Liley HG, et al. Oxytocin receptor antagonists for inhibiting preterm labour. Cochrane Database Syst Rev 2014;30:CD004452. 10.1002/14651858.CD004452.pub3 PMC11086629 24903678

[168] Giorgino FL, Egan CG. Use of isoxsuprine hydrochloride as a tocolytic agent in the treatment of preterm labour: a systematic review of previous literature. Arzneimittelforschung 2010;60:415–20. 10.1055/s-0031-1296305 20712130

[169] Mackeen AD, Seibel-Seamon J, Grimes-Dennis J, et al. Tocolytics for preterm premature rupture of membranes. Cochrane Database Syst Rev 2011:CD007062. 10.1002/14651858.CD007062.pub2 21975760

[170] Saccone G, Suhag A, Berghella V. 17-alpha-hydroxyprogesterone caproate for maintenance tocolysis: a systematic review and metaanalysis of randomized trials. Am J Obstet Gynecol 2015;213:16–22. 10.1016/j.ajog.2015.01.054 25659469

[171] van Vliet E, Dijkema GH, Schuit E, et al. Nifedipine maintenance tocolysis and perinatal outcome: an individual participant data meta-analysis. BJOG 2016;123:1753–60. 10.1111/1471-0528.14249 27550838

[172] Yamasmit W, Chaithongwongwatthana S, Tolosa JE, et al. Prophylactic oral betamimetics for reducing preterm birth in women with a twin pregnancy. Cochrane Database Syst Rev 2012;9:CD004733. 10.1002/14651858.CD004733.pub3 16034944

