# Supplementary material for: Interventions for the prevention of spontaneous preterm birth: a scoping review of systematic reviews
Source: BMJ Open. 2022 May 13;12(5):e052576. doi: 10.1136/bmjopen-2021-052576 (PMC9109033; doi:10.1136/bmjopen-2021-052576)
Supplement: Author's manuscript [file bmjopen-2021-052576.draft_revisions.pdf]

BMJ Open

BMJ Open is committed to open peer review. As part of this commitment we make the peer review history of every article we publish publicly available.

When an article is published we post the peer reviewers' comments and the authors' responses online. We also post the versions of the paper that were used during peer review. These are the versions that the peer review comments apply to.

The versions of the paper that follow are the versions that were submitted during the peer review process. They are not the versions of record or the final published versions. They should not be cited or distributed as the published version of this manuscript.

BMJ Open is an open access journal and the full, final, typeset and author-corrected version of record of the manuscript is available on our site with no access controls, subscription charges or pay-per-view fees (http://bmjopen.bmj.com).

If you have any questions on BMJ Open's open peer review process please email info.bmjopen@bmj.com

BMJ Open

Spontaneous Preterm Birth Prevention: A Scoping Review Highlighting an Inverse Pattern of Research with a Lack of Research Evidence from High Burden Settings

Journal:	*BMJ Open*
Manuscript ID	bmjopen-2021-052576
Article Type:	Original research
Date Submitted by the Author:	19-Apr-2021

Complete List of Authors:	Campbell, Fiona; ScHARR<br>Salam, Shumona; The University of Sheffield, Department of Oncology and Metabolism<br>Sutton , Anthea; The University of Sheffield, ScHARR<br>Jayasooriya, Shamanthi; The University of Sheffield, Academic Unit of Primary Medical Care<br>Mitchell, Caroline; The University of Sheffield, Academic Unit of Primary Medical Care<br>Soma-Pillay, Priya; University of Pretoria, Steve Biko Academic Hospital<br>Chauke, Lawrence; University of Witwatersrand Department of Obstetrics and Gynaecology<br>Gillespie, Bronwen; The University of Sheffield, Department of Oncology and Metabolism<br>Narice, Brenda; The University of Sheffield, Department of Oncology and Metabolism<br>Balen, Julie; The University of Sheffield,<br>Amabebe, Emmanuel; The University of Sheffield, Department of Oncology and Metabolism<br>Parris, Kerry ; The University of Sheffield, Department of Oncology and Metabolism<br>Anumba, Dilichukwu; The University of Sheffield, Department of Oncology and Metabolism

Keywords:	Maternal medicine < OBSTETRICS, International health services < HEALTH SERVICES ADMINISTRATION & MANAGEMENT, Public health < INFECTIOUS DISEASES, Health policy < HEALTH SERVICES ADMINISTRATION & MANAGEMENT

SCHOLARONE™
Manuscripts

I, the Submitting Author has the right to grant and does grant on behalf of all authors of the Work (as defined in the below author licence), an exclusive licence and/or a non-exclusive licence for contributions from authors who are: i) UK Crown employees; ii) where BMJ has agreed a CC-BY licence shall apply, and/or iii) in accordance with the terms applicable for US Federal Government officers or employees acting as part of their official duties; on a worldwide, perpetual, irrevocable, royalty-free basis to BMJ Publishing Group Ltd ("BMJ") its licensees and where the relevant Journal is co-owned by BMJ to the co-owners of the Journal, to publish the Work in this journal and any other BMJ products and to exploit all rights, as set out in our licence.

The Submitting Author accepts and understands that any supply made under these terms is made by BMJ to the Submitting Author unless you are acting as an employee on behalf of your employer or a postgraduate student of an affiliated institution which is paying any applicable article publishing charge ("APC") for Open Access articles. Where the Submitting Author wishes to make the Work available on an Open Access basis (and intends to pay the relevant APC), the terms of reuse of such Open Access shall be governed by a Creative Commons licence – details of these licences and which Creative Commons licence will apply to this Work are set out in our licence referred to above.

Other than as permitted in any relevant BMJ Author's Self Archiving Policies, I confirm this Work has not been accepted for publication elsewhere, is not being considered for publication elsewhere and does not duplicate material already published. I confirm all authors consent to publication of this Work and authorise the granting of this licence.

For peer review only

Spontaneous Preterm Birth Prevention: A Scoping Review Highlighting an Inverse Pattern of Research with a Lack of Research Evidence from High Burden Settings

Fiona Campbell[1] Shumona Salam[3], Anthea Sutton[1], Shamanthi Maya Jayasooriya[2], Caroline Mitchell[2] Emmanuel Aamabebe[3] Julie Balen[1] Bronwen Gillespie[3] Kerry Parris[3] Priya Soma-Pillay[5], Lawrence Chauke[4] , Brenda Narrice[3] , Dilichukwu O Anumba[3],

[1]School of Health and Related Research, University of Sheffield, Sheffield, UK
[2]Academic Unit of Primary Medical Care, University of Sheffield, Sheffield, UK
[3]Department of Oncology and Metabolism, University of Sheffield
[4]Witwatersrand, Johannesburg, South Africa.
[5]Steve Biko Academic Hospital and University of Pretoria, Pretoria, South Africa

Corresponding Author:
Fiona Campbell
ScHARR, University of Sheffield, Regent Court, 30 Regent Street, S1 4DA,
f.campbell@sheffield.ac.uk
https://orcid.org/0000-0002-4141-8863

Data extracted from included studies is available on request by email from the corresponding author: f.campbell@sheffield.ac.uk

Work Count: 3,473

Keywords: Preterm Birth, Scoping Review, Health services research, Meta-analyses

Contributors: NIHR provided funding for researcher support, PRIME established study objectives, FC, PSP, LC prepared the protocol, AS designed search strategies, SS, SJ, CM EA, JB, BG, KP, BN collected and analysed or interpreted data. FC prepared manuscript. SS, AS, SJ, CM, EA, JB, BG, KP, PSP, LC, BN, DA edited or read and approved the final manuscript.

Abstract

Background: Globally, 11% of babies are born preterm annually and this is one of the leading causes of neonatal death and under-5 mortality and morbidity with lifelong sequelae in those that survive. Preterm birth (PTB) disproportionately impacts low- and middle income countries **(**LMICs) where the burden is highest. This mapping review sought to map the evidence for interventions that reduce the risk of PTB, focusing on the evidence from LMICs and to describe how context is considered in evidence synthesis

Method: We conducted a Scoping review, to describe this wide topic area. We searched five electronic databases, and contacted experts to identify relevant systematic reviews of interventions to reduce the risk of PTB. Data was extracted and is described narratively.

For peer review only

Results: We included 139 published systematic reviews for analysis. Interventions were categorised as primary or secondary. The interventions where the results showed a greater effect size and consistency across review findings included treatment of syphilis and vaginal candidiasis, vitamin D supplementation and cervical cerclage. Included in the 139 reviews were 1372 unique primary source studies. 113 systematic reviews included data indicating the country in which the primary studies were undertaken. 390 primary studies (28%) were undertaken in LMIC contexts. Of those undertaken in LMIC contexts, 58 (4.2%) were undertaken in a low income (LI) setting. Only 15 (10.8%) of the reviews sought to explore the impact of context on findings, and 26 reviews did not report the settings in which data was derived.

Conclusion: This mapping review highlights the lack of research evidence derived from contexts where the burden of PTB globally is greatest. The lack of rigour in addressing contextual applicability within systematic review methods is also highlighted. This presents a risk of inappropriate and unsafe recommendations for practice within these contexts. It also highlights a need for primary research, developing and testing interventions in low resource settings.

Strengths and Limitations of this Study

This is the first review of reviews of interventions to reduce the risk of preterm birth that has sought to describe the context in which primary studies were undertaken and consider how this relates to the distribution of burden of PTB across the globe. 139 reviews were included, with a total of 1372 primary studies. Only 4.2% of the primary studies were conducted in low resource settings.

We describe a pattern of inverse research, where there is little correlation between burden of disease and the global distribution of clinical trial research. This inverse pattern has not been previously described or quantified in interventions to reduce the risk of preterm birth, the leading cause of under-5 morbidity and mortality.

Few reviews found that the intervention had a positive effect in reducing the risk of preterm birth and none of these findings included data that was derived from trials conducted in low resource settings.

Most reviews describe the context of the primary studies, but few (n=15) sought to explore contextual variation in effect. In 19% of reviews context is not described. This has implications for the application of findings in contexts where underlying mechanisms may influence the cause of PTB.

We were not able to identify the setting of all primary studies where this was not reported and there is a risk that some studies, which have multiple publications may have been double counted.

For peer review only

BACKGROUND

Preterm birth (PTB) is a global and public health priority. Preterm birth (PTB) is defined by the World Health Organization (WHO) as delivery before 37 completed weeks of gestation, with extremely PTB defined as occurring at less than 28 weeks, very preterm delivery occurring between 28 and 32 weeks, and moderate to late PTB occurring from 32 through 36 weeks.[1] It is one of the leading causes of neonatal death and under five mortality and morbidity with lifelong sequelae.[2] Children born prematurely have increased risks of cognitive problems, such as academic underachievement, behavioural problems and cerebral palsy than those born at full term.[3] They are more likely to experience hospital admission due to infection, particularly during infancy[4] For parents, the financial, social and emotional effects are devastating.[3]

The global burden of PTB is falling more heavily on countries with fewer resources to manage the medical, social, and economic complexities of caring for premature infants. Globally, there are approximately 15 million live preterm births each year, which is estimated to be about 11% of all deliveries each year, ranging from about 8.7% in northern Europe to 13.4% in North Africa.[5 6] The majority of PTBs are occur in Low- or Middle Income countries (LMICs) .[6] The highest PTB rates in 2014 occurred in southeast Asia, south Asia and sub-Saharan Africa. Nine of the 11 countries with the highest rates were in Africa. Furthermore, 60% of all PTBs were estimated to have occurred in sub-Saharan Africa and south Asia accounting for just over nine million of the almost 15 million PTBs that occurred worldwide in 2010 resulting in a PTB rate of 12.8% in those settings.

Patterns of PTB differ between high-income countries and LMICs. However, the differences in these patterns, causes and distribution of PTB is unclear and have not been fully explored. PTB is multifactorial in its aetiology and has distinct biological pathways. The aetiologies differ according to gestational age, ethnicity and characteristics unique to each population. In order to redress the burden of PTB in LMICs, additional insight into the causative and associated factors in these settings is required.

While a number of reviews and overview of reviews of interventions to reduce the risk of PTB[7-10] have been undertaken, none of these reviews have explored how many of the primary studies were undertaken in LMIC contexts. It is clear that some interventions that are effective in high income contexts but may be harmful in LMIC settings, such as the use of antenatal corticosteroids[11] and cerclage.[12] It is also possible that treatments effective in HI country contexts may be even more beneficial or appropriate in LMIC contexts, such as nutritional supplements, interventions to increase birth spacing, interventions to improve the accuracy of measuring gestational age.

We have undertaken a broad scoping review of systematic reviews on interventions to reduce the risk of PTB identifying primary studies undertaken in LMICs. This will allow us to identify potential areas for further synthesis of the evidence and also to identify gaps in the research in order to direct future primary research.

For peer review only

Review objectives

1. To identify systematic reviews that have sought to explore the effectiveness, safety and acceptability of interventions to prevent PTB.

2. To map research evidence to global settings to identify the geographical and economic contexts in which evidence is derived.

3. To identify where gaps in the research base exist (for real world, effectiveness, pragmatic studies) in LMIC contexts to inform future research and to generate research priorities.

4. To describe the methods used in meta-analysis to take into account geographical and regional differences in PTB.

METHODS

We used a scoping review methodology[13] to describe the existing evidence (systematic reviews) available across primary and secondary interventions to prevent preterm birth (PTB) published between 2009 and 2019. Systematic scoping draws upon methods described by Arksey & O'Malley (2005)[14] for scoping reviews: "[…a form of knowledge synthesis that addresses an exploratory research question aimed at scoping key concepts, types of evidence, and gaps in research related to a defined area or field by systematically searching, selecting, and synthesizing existing knowledge".[14] The approach enabled us to highlight the evidence gap and to assist with simultaneously undertaking a research prioritisation exercise and guideline development and to inform a programme of research to develop effective postnatal interventions to mitigate PTB in LMIC settings. It also enabled us to generate an interactive mega-map, an interactive table supported on our project website and designed as a visual tool to identify research gaps and facilitate ready access to relevant evidence. https://www.primeglobalhealth.co.uk/evidence-map-2-7-2020.html.

Patient and Public Involvement

This review was undertaken as part of a larger program of research in preterm birth (NIHR Global Health under grant (17/63/26)). The program is informed by key stakeholders and a PPI advisory group comprising of representatives from Sheffield, Bangladesh, and South Africa. The design and questions for the review were informed by consultation with these groups.

Identifying relevant studies

Relevant systematic reviews were identified by systematic searches in the following electronic databases: MEDLINE, The Cochrane Library, PsycINFO, EMBASE and CINAHL. Each database was searched using the database thesaurus and the key word/free text method. The search strategy, incorporated the following limitations: articles written in English, and Human studies only from April 2009 to July 2020.

For peer review only

We began with a framework of interventions identified by two existing reviews[7,8] as these were broad in their focus and encompassed a range of interventions. Any new intervention types identified during the screening process were then added to the map.

The process of study selection was based on inclusion and exclusion criteria as described in Table 1. After removal of duplicates and irrelevant studies, based on the titles and abstracts, all potentially relevant reviews were read in full.

Table 1 Inclusion/ exclusion criteria based on PICOS

Population

- Pregnant women at less than 37 completed weeks gestation without signs of threatened preterm labour or premature rupture of membranes (PPROM).
- Excluded reviews where the study population was defined by co-morbidities

Intervention

- All interventions deliverable during pregnancy to prevent spontaneous preterm birth. (these included clinical, behavioural and nutritional interventions and health systems and policy interventions).
- All interventions assessed the risk of preterm birth.
- Excluded interventions given to pregnant women to improve neonatal outcomes.

Comparators

- We included any comparator, including placebo or alternative treatments

Outcomes

- We included reviews which focused to PTB as an outcome.
- Where it is reported, we state how many of the primary studies measured PTB as an outcome and the resulting data used in the synthesis.

Study design

1. Systematic reviews published between 1/1/2009-31/12/2019, of studies that have evaluated interventions to prevent PTB, or that measured PTB as a relevant outcome.

Outcomes

1. Preterm birth (<28, <34, <37 weeks gestation) .
2. We recorded neonatal outcomes and adverse outcomes if reported within the review.

Data extraction and coding

Data were extracted using an agreed and piloted template and coded in Excel by two reviewers. The following data categories were extracted: number of included studies, review PICO, setting of primary studies, how this was reported and inclusion in the analysis review methods for the analysis of context of study, PTB outcomes, assessment of adverse effects and recommendations for practice and research. Preterm birth rates in LI, LM, UM and HI settings were drawn data published in a rigorous review of national civil registration and vital statistics to determine global, regional and national estimates of levels of preterm birth.[6]

For peer review only

Where reported information allowed, we used the World Bank categories to identify the categories of LMICs identified in the reviews.[151] .

The population, interventions, comparators, outcomes and reviewer conclusions for future research were tabulated and described narratively. The country or countries where the included primary studies were noted, and the methods used in the review for analyses of data from different settings was also recorded and described. We did not contact review authors for missing data.

FINDINGS

Our search identified 9,517 citations which were screened by two reviewers. A third reviewer was also involved where there was a lack of consensus or uncertainty regarding inclusion. Following screening, 424 full text papers were retrieved for data extraction. At data extraction a further 285 were excluded. The process of identifying the included reviews is summarised in figure 1.

We included 139 reviews which addressed a range of primary and secondary interventions and measured the effectiveness of the intervention in reducing the risk of PTB. These are summarised in Table 2. There was a considerable variation in the number of included studies in the reviews for each intervention, reflecting differing research questions objectives (therefore different PICOs) and search strategies.

[1] Low-income economies are defined as those with a GNI per capita, calculated using the World Bank Atlas method, of $995 or less; lower middle-income economies are those with a GNI per capita between $996 and $3,895; upper middle-income economies are those with a GNI per capita between $3,896 and $12,055; high-income economies are those with a GNI per capita of $12,056 or more.

For peer review only

Table 1 Summary of included systematic reviews and settings of primary studies included in the review

Interventions	Number of reviews	Number of primary studies	country NR	country of primary study LI	LM	UM	HI	mixed	studies where setting NK
**Primary prevention interventions**:									
**Health Systems**									
Models of antenatal care delivery (group/specialised)[16-26]	11	68	2	0	2	2	64	0	0
Midwifery led care[27]	1	15	0	0	0	0	15	0	0
Improving ANC coverage[28]	1	34	0	10	15	5	0	0	0
**Health behaviours**									
Smoking cessation[29 30]	2	111	0	0	0	1	110	0	0
Weight management [31-36]	6	70	1	0	2	8	60	0	0
**Nutritional interventions**									
Macronutrient supplements[37 38]	2	34	0	3	9	10	8	4	0
Micronutrient supplements [24-55]	33	481	2	29	82	122	214	6	9
Vitamin D[39-44]	6	75							
Vitamin A[45 46]	2	24							
Vitamin E, C, E and C[47-49]	3	67							
Iron, folic acid, iron and folic acid[50-57]	8	182							
Fish oil[58-62]	5	38							

For peer review only

Zinc[63 64]	2	25							
Calcium[65 66]	2	27							
Iodine[67]	2	14							
Multiple micronutrients[68-70]	3	29							
**Screening and treatment of periodontal disease[71-82]**	12	46	0	0	3	7	36	0	0
**Screening and prevention/treatment of infection**	14	91	2	2	2	6	79	0	2
Asymptomatic bacteriuria[83-86]	4								
Screening and antibiotics for syphilis[87]	1								
Influenza vaccine[88 89]	2								
Lower genital tract infection[90]	1								
UTI[91 92]	2								
Vaginal candidiasis[93]	1								
Nonspecific infection[94 95]	2								
Malaria[96-98]	3	17	0	8	7	2	2	0	0
**Secondary prevention interventions**:									
Cerclage[99-116]	18	123	10	0	7	11	42		51
Bed rest[117-119]	3	40	1	4	0	0	36	0	0
Cervical pessary[120-125]	6	16	0	0	0	1	14	1	0
Progesterone[126-141]	16	59	5	1	7	8	28	4	11
Tocolytics[142-154]	11	167	3	1	0	13	68	0	84

ANC: antenatal care, NK: not known, NR: not reported, LI: low income, LM: low middle, UM: upper middle, HI: high income, UTI: urinary tract infection.

For peer review only - http://bmjopen.bmj.com/site/about/guidelines.xhtml

Context of primary studies

A total of 1372 primary studies was included across all of the 139 reviews, with 390 (28.4%) undertaken in LMICs. Not all of these studies will have been measuring PTB as an outcome but were included within the review which may have been measuring a range of maternal outcomes including PTB. The largest number of primary studies were those evaluating micronutrient supplements (n=481) and tocolytics (n=167). A total of 113 of the reviews described the country in which the primary study was undertaken and so this data was known for 1288 of the included primary studies. Fifteen (15) primary studies were multicentre and included data gathered from LMIC and HI settings, though only three of these studies included low income countries. Three hundred and ninety (28.4%) of the studies were undertaken in LMIC settings. The majority of these (n=122) were in studies that examined the effects of micronutrient supplements. Excluding nutritional interventions studies, the proportion of LMIC primary studies of interventions to reduce PTB accounts for only 17.6% of the included studies.

The number of primary studies undertaken in low income countries represented only 4.2% of the total number of studies, and if the nutritional intervention studies are excluded, they account for only 3.2% of the interventions. Of those primary studies that were undertaken in LMIC settings the numbers within each category differed significantly. The proportion of the studies that are undertaken in LI, LM and UM were 14.9% (n=58), 34.8% (n=136) and 50.2% (n=196) respectively. There are only single trials that have evaluated the impact of progesterone, tocolytics and interventions to increase calorie intake in LI settings. There are no trials that have evaluated smoking cessation, preventing excessive weight gain, prevention and treatment of periodontal disease, flu vaccine and cervical pessaries. The number of trials in each of the country categories within each intervention type are shown in table 2.

When this data is compared alongside data that shows the prevalence of PTB globally it is clear that there is an inverse pattern in the distribution of the data (figure 2).

Figure 2: Rates of PTB and proportion of primary studies undertaken in each setting.

The effectiveness of interventions

The effectiveness of interventions in reducing the risk of PTB was variable with no intervention showing consistent effectiveness across the included reviews. Although interpretation of this data is limited by the lack of quality appraisal of the included reviews, and therefore should be interpreted with caution. Overall, the scoping review demonstrates considerable inconsistency of results of interventions. Of the 139 reviews, 28 reported a reduction in PTB in intervention versus a control, 80% of the reviews found that the intervention had no impact in reducing the risk of PTB. The summary result (relative risk and odds ratio are shown in Figure 3). The results show the reduction in PTB less than 37weeks gestation. In three reviews the intervention was not statistically significant at 37 weeks but was reported as statistically significant at 34 weeks[107], 35 weeks[132] and 36 weeks[126]. Two reviews reported a positive effect of the intervention in reducing risk of preterm birth but reported the outcome on a continuous measure. These included the effectiveness of macronutrient supplements[32] (SMD -0.19 (95% CI -0.34 to -0.04)) and cerclage (mean difference 95% CI 33.98 days (17.88 to 50.08))[104]. The interventions, reporting binary outomes which appear to have the greatest

For peer review only

effect (RR = 0.2-0.4) in reducing PTB are: antibiotics for asymptomatic bacteriuria[84] (RR = 0.34 (95% CI 0.11 to 0.62), the screening and treatment of syphilis[87] (RR = 0.36 (95% CI 0.27-0.47), treatment of vaginal candidiasis[93] (RR = 0.36, (95% CI 0.17 to 0.75). Interventions with moderate effects (RR = 0.4-0.6) included treating lower genital tract infection[90] and vitamin D supplements.[44] Four of the reviews (figure 2) with a positive effect of the intervention considered that the strength of evidence supporting the finding could be considered high and the finding reliable. None of these reviews included studies conducted in low resource settings, and only one included one study in a lower middle income country.

Figure 3 Summary results of systematic reviews of interventions showing reduction in risk of PTB

ANC: antenatal care, RR: relative risk, OR: odds ratio, LGT: lower genital tract, L,M,IC: low, low middle, upper middle income countries.

For peer review only

Dealing with context and generalisability within evidence synthesis

The authors of the included reviews used different approaches to dealing with the contextual variation when pooling data from primary studies, which was either to ignore, document, explore or control differences. Twenty-seven reviews (19.8%) did not describe the setting of the primary study, ignoring variation in outcomes that may arise as a result of these differences. This occurred most frequently in those reviews of cervical cerclage (see Table 2). The majority of the included reviews 91 (67%) documented the country in which the primary study was carried out either within the text, tables of study characteristics or in accompanying appendices, but this was not considered further in terms of its implications for the findings, or application for future practice or research.

Eight reviews[28 37 38 42 44 63 97 155] sought to explore the impact of geographical and economic context by undertaking a subgroup analysis comparing trials conducted in low income settings with those in high income settings or regression analysis with geographical regions as covariates (Africa, Americas, South east Asia, Europe, Eastern Mediterranean, Western Pacific). In addition one study[155] listed the country instead of author name on the forest plot allowing ready visualisation of differences across settings. Nine reviews[29 40 45 48 49 53 65 66 69] undertook subgroup analysis based on features of the population that might vary across settings and influence the effectiveness of the intervention, such as baseline nutritional status of the mother. One review[70] exploring multiple micronutrient supplementation controlled for settings by limiting the review to include only those studies undertaken in LMIC contexts. Four reviews[70 100 126 132] undertook an IPD (individual patient data) analyses allowing subgroup analyses about differences in effect more easily than with aggregate data. This approach allowed comparison between effects for women recruited and receiving the intervention in different settings, effect sizes in each country could also be shown in the analyses.

DISCUSSION

This scoping review has revealed an inverse pattern of research, with only 28.% of published research included in systematic reviews of interventions reporting PTB outcomes, carried out in LMIC settings, and only 4.2% conducted in the poorest countries in the world where the burden of preterm birth is greatest. The distribution of types of intervention tested and evaluated in these settings is not evenly distributed across interventions, but is largely focused on very context specific interventions (prevention of malarial infection) and nutritional supplementation. Similar patterns of a mismatch between research effort and health needs in non- high income regions have been identified across a broad range of diseases.[156 157] It has also been previously reported that primary research often fails to capture those with the greatest health care needs such as vulnerable populations.[158 159]

This review has also revealed a limited approach in evidence synthesis to explore the applicability of findings across geographical settings and to draw attention to these gaps with a resultant risk that interventions shown to be effective in HI settings may not translate to LI settings and may indeed have adverse effects when applied to LI settings. Likewise, the focus of research in HI settings means that interventions that may have greater benefit in LI settings, where the problem is greatest remain untested or replicated with larger numbers of participants. Adolescent pregnancy, short inter pregnancy interval have been highlighted as important risk factors for PTB[160] yet there is a lack of data on interventions to address these and their effectiveness in reducing the risk of PTB.

For peer review only

The lack of robust evidence to inform both the primary and secondary prevention of PTB in low resource settings, where the prevalence of PTB is highest presents challenges for developing appropriate and contextually relevant clinical guidance.

Two recent overviews of reviews[9 10] also found that few interventions are effective in PTB prevention. The following interventions were identified in these reviews as showing positive or possible benefit: lifestyle and behavioural changes (including diet and exercise); nutritional supplements (including calcium zinc and vitamin D supplementation); nutritional education; screening for lower genital tract infections. Positive effects of secondary interventions were found for low dose aspirin among women at risk of preeclampsia; clindamycin for treatment of bacterial vaginosis; treatment of vaginal candidiasis; progesterone in women with prior spontaneous PTB and in those with short midtrimester cervical length; L-arginine in women at risk for preeclampsia; levothyroxine among women with thyroid disease; calcium supplementation in women at risk of hypertensive disorders; smoking cessation; cervical length screening in women with history of PTB with placement of cerclage in those with short cervix; cervical pessary in singleton gestations with short cervix; and treatment of periodontal disease. Our review findings were in concordance, although, in addition, we identified screening and antibiotic treatment for syphilis, and positive effects of fish oil supplements. In most instances the trials were small and authors recommended larger well-designed RCTs. The lack of consistency across review findings for interventions also merits more exploration. Compromised methodological rigour can inflate trial findings by 30% to 50%. Some of the differences in our review findings reflect some differences in the included reviews.

The interventions identified in this review, and those of Matei et al (2019)[9] and Medley et al (2019)[10] informing guideline development, clinical practice and policy decision making have been little tested in LMIC settings. In those interventions where there is more consistency in review findings such as cervical cerclage, there are no studies that have been conducted in low income settings and over half of the reviews did not report or consider settings in their analyses.

This scoping review has shown that many authors of systematic reviews fail to use design and statistical approaches that adequately address contextual variations between the included source studies and imperfectly represent 'real world' conditions within the target context (Higgins et al 2019). While those reviews that sought to take into account LMIC contexts were unable to conduct the analyses due to a lack of data, they nonetheless were able to highlight the gaps in research, for example the lack of studies in vitamin D undertaken in Africa.[42]

The Preferred Reporting Items for Systematic reviews and Meta-analyses (PRISMA) reporting standards reference 'context' in terms of the circumstances requiring the review itself, rather than referencing the contexts of studies included in the review(Moher et al 2009) The PRISMA extension for Complex Interventions includes the elements of 'time' and 'setting' (Guise et al 2017). However, grouping LMIC data, or even LI data may still be too broad. Even within the categories of Low income there is considerable diversity that may impact on how an intervention works and within countries there may also be considerable diversity. For example, the time taken to reach comprehensive emergency obstetric care facilities in low resource settings is often underestimated and for most women is likely to be 120 minutes of travel time.[161] Context cannot be standardised, it will vary from review to review, as different interventions and different populations are considered. 'Context' and the factors that might influence the efficacy, uptake, acceptability, appropriateness, accessibility and

For peer review only

availability of an intervention requires a good understanding of the aetiology and mechanisms by which risk factors interact with environmental, microbial, socio-political and health system variations across settings

It must be acknowledged that there are significant barriers to undertaking research in many settings across the globe. These include very practical challenges such as a lack of access to high quality data and the challenges of estimating gestational age.[162] Recent reductions in funding of global health research by the UK government,[163] will undermine what has been a growth in research in LMIC settings and will impede efforts to address the imbalances highlighted in this scoping review

A number of limitations exist in this scoping review. We have not sought to identify the setting of primary studies where this is not reported in the systematic review. We have also not limited our analysis to studies within the review that only contributed findings to the risk of PTB. Most reviews are exploring several maternal and infant outcomes. Included primary studies in this scoping review may therefore have not have included PTB outcome data. Nevertheless, it gives an indication of the distribution of research being undertaken in the poorest regions of the world that address preterm birth.

CONCLUSION

Only 4.2% of primary research to examine the effectiveness of interventions to reduce the risk of preterm birth is carried out in those settings where the burden of preterm birth is greatest. No interventions which reduce the risk of PTB, judged to be supported by strong evidence, include studies undertaken in low resource settings. In the synthesis of studies, current methods often fail to address the contextual variation and consider the applicability of findings in low resource, high burden settings. This has implications for supporting policy making, and development of contextually relevant clinical guidelines. While methods can be undertaken to improve approaches to evidence synthesis, they cannot compensate for the lack of primary research in low resource settings. This is critical if global health inequalities are to be addressed and millennium development goals[164] to reduce under-five mortality are to be achieved.

For peer review only

References

1. WHO. WHO: recommended definitions, terminology and format for statistical tables related to the perinatal period and use of a new certificate for cause of perinatal deaths. Modifications recommended by FIGO as amended October 14, 1976. . *Acta Obstet Gynecol Scand* 1977;1977; 56: 247–53.
2. Liu L, Oza S, Hogan D, et al. Global, regional, and national causes of child mortality in 2000–13, with projections to inform post-2015 priorities: an updated systematic analysis. *The Lancet* 2015;385(9966):430-40.
3. Brydges CR, Landes JK, Reid CL, et al. Cognitive outcomes in children and adolescents born very preterm: a meta-analysis. *Developmental Medicine & Child Neurology* 2018;60(5):452-68.
4. Coathup V, Boyle E, Carson C, et al. Gestational age and hospital admissions during childhood: population based, record linkage study in England (TIGAR study). *bmj* 2020;371
5. Howson CP, Kinney MV, McDougall L, et al. Born too soon: preterm birth matters. *Reproductive health* 2013;10(1):1-9.
6. Chawanpaiboon S, Vogel JP, Moller A-B, et al. Global, regional, and national estimates of levels of preterm birth in 2014: a systematic review and modelling analysis. *The Lancet Global Health* 2019;7(1):e37-e46.
7. Barros FC, Bhutta ZA, Batra M, et al. Global report on preterm birth and stillbirth (3 of 7): evidence for effectiveness of interventions. *BMC pregnancy and childbirth* 2010;10(1):1-36.
8. Bhutta ZA, Das JK, Bahl R, et al. Can available interventions end preventable deaths in mothers, newborn babies, and stillbirths, and at what cost? *The Lancet* 2014;384(9940):347-70.
9. Matei A, Saccone G, Vogel JP, et al. Primary and secondary prevention of preterm birth: a review of systematic reviews and ongoing randomized controlled trials. *European Journal of Obstetrics & Gynecology and Reproductive Biology* 2019;236:224-39.
10. Medley N, Vogel JP, Care A, et al. Interventions during pregnancy to prevent preterm birth: an overview of Cochrane systematic reviews. *Cochrane Database of Systematic Reviews* 2018(11)
11. Opiyo N, Stones W. Corticosteroids for preterm deliveries: missing evidence. *Cochrane Database Syst Rev* 2017;5:ED000121.
12. Egwuatu V. Complications of cervical cerclage in Igbo women. *Journal of the National Medical Association* 1986;78(3):245.
13. White H, Albers B, Gaarder M, et al. Guidance for producing a Campbell evidence and gap map. *Campbell Systematic Reviews* 2020;16(4):e1125.
14. Arksey H, O'Malley L. Scoping studies: towards a methodological framework. *International journal of social research methodology* 2005;8(1):19-32.
15. Bank TW. world Bank country and Lending Groups 2021 [Available from: https://datahelpdesk.worldbank.org/knowledgebase/articles/906519 accessed 18/03/2021.
16. Allen J, Gamble J, Stapleton H, et al. Does the way maternity care is provided affect maternal and neonatal outcomes for young women? A review of the research literature (Structured abstract). *Women and Birth* 2012;25(2):54-63.

For peer review only

17. Catling CJ, Medley N, Foureur M, et al. Group versus conventional antenatal care for women. *Cochrane Database of Systematic Reviews* 2015(2):CD007622.
18. Dodd JM, Dowswell T, Crowther CA. Specialised antenatal clinics for women with a multiple pregnancy for improving maternal and infant outcomes. *Cochrane Database of Systematic Reviews* 2015(11) doi: 10.1002/14651858.CD005300.pub4
19. Dowswell T, Carroli G, Duley L, et al. Alternative versus standard packages of antenatal care for low-risk pregnancy. *Cochrane Database of Systematic Reviews* 2015(7):CD000934.
20. Dowswell T, Middleton P, Weeks A. Antenatal day care units versus hospital admission for women with complicated pregnancy. *Cochrane Database of Systematic Reviews* 2009(4) doi: 10.1002/14651858.CD001803.pub2
21. Fernandez Turienzo C, Sandall J, Peacock JL. Models of antenatal care to reduce and prevent preterm birth: a systematic review and meta-analysis. *BMJ Open* 2016;6(1):e009044.
22. Lathrop B. A systematic review comparing group prenatal care to traditional prenatal care. *Nursing for Women's Health* 2013;17(2):118-30.
23. Malouf R, Redshaw M. Specialist antenatal clinics for women at high risk of preterm birth: a systematic review of qualitative and quantitative research. *BMC Pregnancy & Childbirth* 2017;17(1):51.
24. Ruiz-Mirazo E, Lopez-Yarto M, McDonald SD. Group prenatal care versus individual prenatal care: a systematic review and meta-analyses. *Journal of Obstetrics & Gynaecology Canada: JOGC* 2012;34(3):223-29.
25. Sheeder J, Weber Yorga K, Kabir-Greher K. A review of prenatal group care literature: the need for a structured theoretical framework and systematic evaluation. *Maternal & Child Health Journal* 2012;16(1):177-87.
26. Whitworth M, Quenby S, Cockerill RO, et al. Specialised antenatal clinics for women with a pregnancy at high risk of preterm birth (excluding multiple pregnancy) to improve maternal and infant outcomes. *Cochrane Database of Systematic Reviews* 2011(9):CD006760.
27. Sandall J, Soltani H, Gates S, et al. Midwife-led continuity models versus other models of care for childbearing women. *Cochrane Database of Systematic Reviews* 2013(8):CD004667.
28. Mbuagbaw L, Medley N, Darzi AJ, et al. Health system and community level interventions for improving antenatal care coverage and health outcomes. *Cochrane Database of Systematic Reviews* 2015(12) doi: 10.1002/14651858.CD010994.pub2
29. Chamberlain C, O'Mara-Eves A, Oliver S, et al. Psychosocial interventions for supporting women to stop smoking in pregnancy. *Cochrane Database of Systematic Reviews* 2013(10):CD001055.
30. Coleman T, Chamberlain C, Davey MA, et al. Pharmacological interventions for promoting smoking cessation during pregnancy. *Cochrane Database of Systematic Reviews* 2012(9):CD010078.
31. Dodd J, Grivell R, Crowther C, et al. Antenatal interventions for overweight or obese pregnant women: a systematic review of randomised trials (Structured abstract). *BJOG An International Journal of Obstetrics and Gynaecology* 2010;117(11):1316-26.
32. Gresham E, Bisquera A, Byles JE, et al. Effects of dietary interventions on pregnancy outcomes: a systematic review and meta-analysis. *Maternal & Child Nutrition* 2016;12(1):5-23.

For peer review only

33. Muktabhant B, Lawrie TA, Lumbiganon P, et al. Diet or exercise, or both, for preventing excessive weight gain in pregnancy. *Cochrane Database of Systematic Reviews* 2015(6):CD007145.
34. Shepherd E, Gomersall JC, Tieu J, et al. Combined diet and exercise interventions for preventing gestational diabetes mellitus. *Cochrane Database of Systematic Reviews* 2017(11) doi: 10.1002/14651858.CD010443.pub3
35. Tanentsapf I, Heitmann BL, Adegboye AR. Systematic review of clinical trials on dietary interventions to prevent excessive weight gain during pregnancy among normal weight, overweight and obese women. *BMC Pregnancy & Childbirth* 2011;11:81.
36. Thangaratinam S, Rogozinska E, Jolly K, et al. Interventions to reduce or prevent obesity in pregnant women: a systematic review. *Health Technology Assessment (Winchester, England)* 2012;16(31):iii-iv, 1-191.
37. Girard AW, Olude O. Nutrition education and counselling provided during pregnancy: effects on maternal, neonatal and child health outcomes. *Paediatric and perinatal epidemiology* 2012;26:191-204.
38. Ota E, Hori H, Mori R, et al. Antenatal dietary education and supplementation to increase energy and protein intake. *Cochrane Database of Systematic Reviews* 2015(6):CD000032.
39. Bi WG, Nuyt AM, Weiler H, et al. Association Between Vitamin D Supplementation During Pregnancy and Offspring Growth, Morbidity, and Mortality: A Systematic Review and Meta-analysis. *JAMA Pediatrics* 2018;172(7):635-45.
40. Palacios C, De-Regil LM, Lombardo LK, et al. Vitamin D supplementation during pregnancy: Updated meta-analysis on maternal outcomes. *Journal of Steroid Biochemistry & Molecular Biology* 2016;164:148-55.
41. Perez-Lopez FR, Pasupuleti V, Mezones-Holguin E, et al. Effect of vitamin D supplementation during pregnancy on maternal and neonatal outcomes: a systematic review and meta-analysis of randomized controlled trials. *Fertility & Sterility* 2015;103(5):1278-88.e74.
42. Roth DE, Leung M, Mesfin E, et al. Vitamin D supplementation during pregnancy: state of the evidence from a systematic review of randomised trials. *Bmj* 2017;359:j5237.
43. Thorne-Lyman A, Fawzi WW. Vitamin D during pregnancy and maternal, neonatal and infant health outcomes: a systematic review and meta-analysis. *Paediatric and Perinatal Epidemiology* 2012;26 Suppl 1:75-90.
44. Zhou SS, Tao YH, Huang K, et al. Vitamin D and risk of preterm birth: Up-to-date meta-analysis of randomized controlled trials and observational studies. *Journal of Obstetrics & Gynaecology Research* 2017;43(2):247-56.
45. McCauley ME, van den Broek N, Dou L, et al. Vitamin A supplementation during pregnancy for maternal and newborn outcomes. *Cochrane Database of Systematic Reviews* 2015(10):CD008666.
46. Thorne-Lyman AL, Fawzi WW. Vitamin A and carotenoids during pregnancy and maternal, neonatal and infant health outcomes: a systematic review and meta-analysis. *Paediatric and Perinatal Epidemiology* 2012;26 Suppl 1:36-54.
47. Rahimi R, Nikfar S, Rezaie A, et al. A meta-analysis on the efficacy and safety of combined vitamin C and E supplementation in preeclamptic women. *Hypertension in Pregnancy* 2009;28(4):417-34.
48. Rumbold A, Ota E, Hori H, et al. Vitamin E supplementation in pregnancy. *Cochrane Database of Systematic Reviews* 2015(9):CD004069.

For peer review only

49. Rumbold A, Ota E, Nagata C, et al. Vitamin C supplementation in pregnancy. *Cochrane Database of Systematic Reviews* 2015(9):CD004072.
50. Lassi ZS, Salam RA, Haider BA, et al. Folic acid supplementation during pregnancy for maternal health and pregnancy outcomes. *Cochrane Database of Systematic Reviews* 2013(3):CD006896.
51. Mantovani E, Filippini F, Bortolus R, et al. Folic acid supplementation and preterm birth: results from observational studies. *BioMed Research International* 2014;2014:481914.
52. Pena-Rosas JP, De-Regil LM, Dowswell T, et al. Intermittent oral iron supplementation during pregnancy. *Cochrane Database of Systematic Reviews* 2012(7):CD009997.
53. Peña-Rosas JP, De-Regil LM, Garcia-Casal MN, et al. Daily oral iron supplementation during pregnancy. *Cochrane Database of Systematic Reviews* 2015(7) doi: 10.1002/14651858.CD004736.pub5
54. Pena-Rosas JP, Viteri FE. Effects and safety of preventive oral iron or iron+folic acid supplementation for women during pregnancy. *Cochrane Database of Systematic Reviews* 2009(4):CD004736.
55. Saccone G, Berghella V. Folic acid supplementation in pregnancy to prevent preterm birth: a systematic review and meta-analysis of randomized controlled trials. *European Journal of Obstetrics, Gynecology, & Reproductive Biology* 2016;199:76-81.
56. Zhang Q, Wang Y, Xin X, et al. Effect of folic acid supplementation on preterm delivery and small for gestational age births: A systematic review and meta-analysis. *Reproductive Toxicology* 2017;67:35-41.
57. Imdad A, Bhutta Z. Routine iron/folate supplementation during pregnancy: effect on maternal anaemia and birth outcomes (Structured abstract). *Paediatric and Perinatal Epidemiology* 2012;26(Supplement 1):168-77.
58. Chen B, Ji X, Zhang L, et al. Fish oil supplementation improves pregnancy outcomes and size of the newborn: a meta-analysis of 21 randomized controlled trials. *Journal of Maternal-Fetal & Neonatal Medicine* 2016;29(12):2017-27.
59. Kar S, Wong M, Rogozinska E, et al. Effects of omega-3 fatty acids in prevention of early preterm delivery: a systematic review and meta-analysis of randomized studies. *European Journal of Obstetrics, Gynecology, & Reproductive Biology* 2016;198:40-46.
60. Saccone G, Berghella V. Omega-3 supplementation to prevent recurrent preterm birth: a systematic review and metaanalysis of randomized controlled trials. *American Journal of Obstetrics & Gynecology* 2015;213(2):135-40.
61. Salvig JD, Lamont RF. Evidence regarding an effect of marine n-3 fatty acids on preterm birth: a systematic review and meta-analysis. *Acta Obstetricia et Gynecologica Scandinavica* 2011;90(8):825-38.
62. Saccone G, Saccone I, Berghella V. Omega-3 long-chain polyunsaturated fatty acids and fish oil supplementation during pregnancy: which evidence? *Journal of Maternal-Fetal & Neonatal Medicine* 2016;29(15):2389-97.
63. Chaffee BW, King JC. Effect of zinc supplementation on pregnancy and infant outcomes: a systematic review. *Paediatric and Perinatal Epidemiology* 2012;26 Suppl 1:118-37.
64. Mori R, Ota E, Middleton P, et al. Zinc supplementation for improving pregnancy and infant outcome. *Cochrane Database of Systematic Reviews* 2012(7):CD000230.
65. Hofmeyr GJ, Lawrie TA, Atallah ÁN, et al. Calcium supplementation during pregnancy for preventing hypertensive disorders and related problems. *Cochrane database of systematic reviews* 2018(10)

For peer review only

66. Hofmeyr GJ, Manyame S, Medley N, et al. Calcium supplementation commencing before or early in pregnancy, for preventing hypertensive disorders of pregnancy. *Cochrane Database of Systematic Reviews* 2019(9)
67. Harding KB, Pena-Rosas JP, Webster AC, et al. Iodine supplementation for women during the preconception, pregnancy and postpartum period. *Cochrane Database of Systematic Reviews* 2017;3:CD011761.
68. Fall CH, Fisher DJ, Osmond C, et al. Multiple micronutrient supplementation during pregnancy in low-income countries: a meta-analysis of effects on birth size and length of gestation. *Food & Nutrition Bulletin* 2009;30(4 Suppl):S533-46.
69. Keats EC, Haider BA, Tam E, et al. Multiple-micronutrient supplementation for women during pregnancy. *Cochrane Database of Systematic Reviews* 2019(3)
70. Smith ER, Shankar AH, Wu LS, et al. Modifiers of the effect of maternal multiple micronutrient supplementation on stillbirth, birth outcomes, and infant mortality: a meta-analysis of individual patient data from 17 randomised trials in low-income and middle-income countries. *The Lancet Global Health* 2017;5(11):e1090-e100.
71. Corbella S, Del Fabbro M, Taschieri S, et al. Periodontal disease and adverse pregnancy outcomes: A systematic review. *Italian Oral Surgery* 2012;11(4):132-46.
72. Fogacci MF, Vettore MV, Leao AT. The effect of periodontal therapy on preterm low birth weight: a meta-analysis. *Obstetrics & Gynecology* 2011;117(1):153-65.
73. George A, Shamim S, Johnson M, et al. Periodontal treatment during pregnancy and birth outcomes: a meta-analysis of randomised trials. *International Journal of Evidence-Based Healthcare* 2011;9(2):122-47.
74. Pimentel Lopes De Oliveira GJ, Amaral Fontanari L, Chaves De Souza JA, et al. Effect of periodontal treatment on the incidence of preterm delivery: a systematic review. *Minerva Stomatologica* 2010;59(10):543-50.
75. Polyzos N, Polyzos I, Zavos A, et al. Obstetric outcomes after treatment of periodontal disease during pregnancy: systematic review and meta-analysis (Structured abstract). *Bmj* 2010;341(2):c7017.
76. Rosa MI, Pires PD, Medeiros LR, et al. Periodontal disease treatment and risk of preterm birth: a systematic review and meta-analysis. *Cadernos de Saude Publica* 2012;28(10):1823-33.
77. Shah M, Muley A, Muley P. Effect of nonsurgical periodontal therapy during gestation period on adverse pregnancy outcome: a systematic review. *Journal of Maternal-Fetal & Neonatal Medicine* 2013;26(17):1691-95.
78. Uppal A, Uppal S, Pinto A, et al. The effectiveness of periodontal disease treatment during pregnancy in reducing the risk of experiencing preterm birth and low birth weight: a meta-analysis. *Journal of the American Dental Association* 2010;141(12):1423-34.
79. Kim AJ, Lo AJ, Pullin DA, et al. Scaling and root planing treatment for periodontitis to reduce preterm birth and low birth weight: a systematic review and meta-analysis of randomized controlled trials. *Journal of Periodontology* 2012;83(12):1508-19.
80. da Silva HEC, Stefani CM, de Santos Melo N, et al. Effect of intra-pregnancy nonsurgical periodontal therapy on inflammatory biomarkers and adverse pregnancy outcomes: a systematic review with meta-analysis. *Systematic Reviews* 2017;6(1):197.
81. Iheozor-Ejiofor Z, Middleton P, Esposito M, et al. Treating periodontal disease for preventing adverse birth outcomes in pregnant women. *Cochrane Database of Systematic Reviews* 2017;6:CD005297.

For peer review only

82. Schwendicke F, Karimbux N, Allareddy V, et al. Periodontal treatment for preventing adverse pregnancy outcomes: a meta- and trial sequential analysis. *PLoS ONE [Electronic Resource]* 2015;10(6):e0129060.
83. Guinto VT, De GB, Festin MR, et al. Different antibiotic regimens for treating asymptomatic bacteriuria in pregnancy. *Cochrane Database of Systematic Reviews* 2010(9) doi: 10.1002/14651858.CD007855.pub2
84. Smaill FM, Vazquez JC. Antibiotics for asymptomatic bacteriuria in pregnancy. *Cochrane Database of Systematic Reviews* 2015(8):CD000490.
85. Widmer M, Lopez I, Gülmezoglu AM, et al. Duration of treatment for asymptomatic bacteriuria during pregnancy. *Cochrane Database of Systematic Reviews* 2015(11)
86. Angelescu K, Nussbaumer-Streit B, Sieben W, et al. Benefits and harms of screening for and treatment of asymptomatic bacteriuria in pregnancy: a systematic review. *BMC Pregnancy & Childbirth* 2016;16(1):336.
87. Blencowe H, Cousens S, Kamb M, et al. Lives Saved Tool supplement detection and treatment of syphilis in pregnancy to reduce syphilis related stillbirths and neonatal mortality. *BMC Public Health* 2011;11 Suppl 3:S9.
88. Fell DB, Platt RW, Lanes A, et al. Fetal death and preterm birth associated with maternal influenza vaccination: systematic review. *BJOG: An International Journal of Obstetrics & Gynaecology* 2015;122(1):17-26.
89. Zhang C, Wang X, Liu D, et al. A systematic review and meta-analysis of fetal outcomes following the administration of influenza A/H1N1 vaccination during pregnancy. *International Journal of Gynaecology & Obstetrics* 2018;141(2):141-50.
90. Sangkomkamhang US, Lumbiganon P, Prasertcharoensuk W, et al. Antenatal lower genital tract infection screening and treatment programs for preventing preterm delivery. *Cochrane Database of Systematic Reviews* 2015(2) doi: 10.1002/14651858.CD006178.pub3
91. Schneeberger C, Geerlings SE, Middleton P, et al. Interventions for preventing recurrent urinary tract infection during pregnancy. *Cochrane Database of Systematic Reviews* 2015(7) doi: 10.1002/14651858.CD009279.pub3
92. Vazquez JC, Abalos E. Treatments for symptomatic urinary tract infections during pregnancy. *Cochrane Database of Systematic Reviews* 2011(1):CD002256.
93. Roberts CL, Algert CS, Rickard KL, et al. Treatment of vaginal candidiasis for the prevention of preterm birth: a systematic review and meta-analysis. *Systematic Reviews* 2015;4:31.
94. Flenady V, Hawley G, Stock OM, et al. Prophylactic antibiotics for inhibiting preterm labour with intact membranes. *Cochrane Database of Systematic Reviews* 2013(12):CD000246.
95. Thinkhamrop J, Hofmeyr GJ, Adetoro O, et al. Antibiotic prophylaxis during the second and third trimester to reduce adverse pregnancy outcomes and morbidity. *Cochrane Database of Systematic Reviews* 2015(6):CD002250.
96. "C. K. Manyando K, Dalessandro U, Okafor HU, et al. A systematic review of the safety and efficacy of artemether-lumefantrine against uncomplicated Plasmodium falciparum malaria during pregnancy. *Malaria Journal* 2012;11 (no pagination)(141)
97. Radeva-Petrova D, Kayentao K, ter Kuile FO, et al. Drugs for preventing malaria in pregnant women in endemic areas: any drug regimen versus placebo or no treatment. *Cochrane Database of Systematic Reviews* 2014(10):CD000169.

For peer review only

98. Gamble C, Ekwaru PJ, Garner P, et al. Insecticide-treated nets for the prevention of malaria in pregnancy: a systematic review of randomised controlled trials. *PLoS Med* 2007;4(3):e107.
99. Alfirevic Z, Stampalija T, Medley N. Cervical stitch (cerclage) for preventing preterm birth in singleton pregnancy. *Cochrane Database of Systematic Reviews* 2017;6:CD008991.
100. Berghella V, Ciardulli A, Rust OA, et al. Cerclage for sonographic short cervix in singleton gestations without prior spontaneous preterm birth: systematic review and meta-analysis of randomized controlled trials using individual patient-level data. *Ultrasound in Obstetrics & Gynecology* 2017;50(5):569-77.
101. Berghella V, Keeler SM, To MS, et al. Effectiveness of cerclage according to severity of cervical length shortening: a meta-analysis. *Ultrasound in Obstetrics & Gynecology* 2010;35(4):468-73.
102. Berghella V, Rafael TJ, Szychowski JM, et al. Cerclage for short cervix on ultrasonography in women with singleton gestations and previous preterm birth: a meta-analysis. *Obstetrics & Gynecology* 2011;117(3):663-71.
103. DeFranco E, Valent A, Newman T, et al. Adjunctive therapies to cerclage for the prevention of preterm birth: a systematic review (Provisional abstract). *Obstetrics and Gynecology International* 2013;2013(2):528158.
104. Ehsanipoor RM, Seligman NS, Saccone G, et al. Physical Examination-Indicated Cerclage: A Systematic Review and Meta-analysis. *Obstetrics & Gynecology* 2015;126(1):125-35.
105. Moawad GN, Tyan P, Bracke T, et al. Systematic Review of Transabdominal Cerclage Placed via Laparoscopy for the Prevention of Preterm Birth. *Journal of Minimally Invasive Gynecology* 2018;25(2):277-86.
106. Namouz S, Porat S, Okun N, et al. Emergency cerclage: literature review (Provisional abstract). *Obstetrical and Gynecological Survey* 2013;68(5):379-88.
107. Pergialiotis V, Vlachos DG, Prodromidou A, et al. Double versus single cervical cerclage for the prevention of preterm births. *Journal of Maternal-Fetal & Neonatal Medicine* 2015;28(4):379-85.
108. Rafael TJ, Berghella V, Alfirevic Z. Cervical stitch (cerclage) for preventing preterm birth in multiple pregnancy. *Cochrane Database of Systematic Reviews* 2014(9):CD009166.
109. Saccone G, Rust O, Althuisius S, et al. Cerclage for short cervix in twin pregnancies: systematic review and meta-analysis of randomized trials using individual patient-level data. *Acta Obstetricia et Gynecologica Scandinavica* 2015;94(4):352-58.
110. Smith J, DeFranco EA. Tocolytics used as adjunctive therapy at the time of cerclage placement: a systematic review. *Journal of Perinatology* 2015;35(8):561-65.
111. Zeybek B, Hill A, Menderes G, et al. Robot-Assisted Abdominal Cerclage During Pregnancy. *Journal of the Society of Laparoendoscopic Surgeons* 2016;20(4):Oct-Dec.
112. Liu X, Luo X, Xiao X, et al. Cervical cerclage for preventing preterm birth in twin pregnancies. A systematic review and meta-analysis (Provisional abstract). *Database of Abstracts of Reviews of Effects* 2013(4):632-38.
113. Conde-Agudelo A, Romero R, Da Fonseca E, et al. Vaginal progesterone is as effective as cervical cerclage to prevent preterm birth in women with a singleton gestation, previous spontaneous preterm birth, and a short cervix: updated indirect comparison meta-analysis. *American Journal of Obstetrics & Gynecology* 2018;219(1):10-25.

For peer review only

114. Conde-Agudelo A, Romero R, Nicolaides K, et al. Vaginal progesterone vs. cervical cerclage for the prevention of preterm birth in women with a sonographic short cervix, previous preterm birth, and singleton gestation: a systematic review and indirect comparison metaanalysis. *American Journal of Obstetrics & Gynecology* 2013;208(1):42.e41-42.e18.
115. Jarde A, Lutsiv O, Park CK, et al. Preterm birth prevention in twin pregnancies with progesterone, pessary, or cerclage: a systematic review and meta-analysis. *BJOG: An International Journal of Obstetrics & Gynaecology* 2017;124(8):1163-73.
116. Jarde A, Lutsiv O, Park CK, et al. Effectiveness of progesterone, cerclage and pessary for preventing preterm birth in singleton pregnancies: a systematic review and network meta-analysis. *BJOG: An International Journal of Obstetrics & Gynaecology* 2017;124(8):1176-89.
117. Crowther CA, Han S. Hospitalisation and bed rest for multiple pregnancy. *Cochrane Database of Systematic Reviews* 2010(7):CD000110.
118. Maloni J. Antepartum bed rest for pregnancy complications: efficacy and safety for preventing preterm birth (Provisional abstract). *Biological Research for Nursing* 2010;12(2):106-24.
119. Sosa CG, Althabe F, Belizan JM, et al. Bed rest in singleton pregnancies for preventing preterm birth. *Cochrane Database of Systematic Reviews* 2015(3):CD003581.
120. Jin Z, Chen L, Qiao D, et al. Cervical pessary for preventing preterm birth: a meta-analysis. *Journal of Maternal-Fetal and Neonatal Medicine* 2017:1-7.
121. Liem SMS, van Pampus MG, Mol BWJ, et al. Cervical Pessaries for the Prevention of Preterm Birth: A Systematic Review. *Obstetrics and Gynecology International* 2013;2013:576723. doi: 10.1155/2013/576723
122. Saccone G, Ciardulli A, Xodo S, et al. Cervical pessary for preventing preterm birth in twin pregnancies with short cervical length: a systematic review and meta-analysis. *Journal of Maternal-Fetal & Neonatal Medicine* 2017;30(24):2918-25.
123. Saccone G, Ciardulli A, Xodo S, et al. Cervical Pessary for Preventing Preterm Birth in Singleton Pregnancies with Short Cervical Length: A Systematic Review and Meta-analysis. *Obstetrical and Gynecological Survey* 2018;73(1):13-14.
124. Thangatorai R, Lim FC, Nalliah S. Cervical pessary in the prevention of preterm births in multiple pregnancies with a short cervix: PRISMA compliant systematic review and meta-analysis. *Journal of Maternal-Fetal & Neonatal Medicine* 2018;31(12):1638-45.
125. Zheng L, Dong J, Dai Y, et al. Cervical pessaries for the prevention of preterm birth: a systematic review and meta-analysis. *Journal of Maternal-Fetal & Neonatal Medicine* 2017:1-10.
126. Romero R, Conde-Agudelo A, Da Fonseca E, et al. Vaginal progesterone for preventing preterm birth and adverse perinatal outcomes in singleton gestations with a short cervix: a meta-analysis of individual patient data. *American Journal of Obstetrics & Gynecology* 2018;218(2):161-80.
127. Romero R, Nicolaides KH, Conde-Agudelo A, et al. Vaginal progesterone decreases preterm birth<=34weeks of gestation in women with a singleton pregnancy and a short cervix: an updated meta-analysis including data from the OPPTIMUM study. *Ultrasound in Obstetrics & Gynecology* 2016;48(3):308-17.
128. Saccone G, Khalifeh A, Elimian A, et al. Vaginal progesterone vs intramuscular 17alpha-hydroxyprogesterone caproate for prevention of recurrent spontaneous preterm

For peer review only

birth in singleton gestations: systematic review and meta-analysis of randomized controlled trials. *Ultrasound in Obstetrics & Gynecology* 2017;49(3):315-21.
129. Su LL, Samuel M, Chong YS. Progestational agents for treating threatened or established preterm labour. *Cochrane Database of Systematic Reviews* 2010(1):CD006770.
130. Suhag A, Saccone G, Berghella V. Vaginal progesterone for maintenance tocolysis: a systematic review and metaanalysis of randomized trials. *American Journal of Obstetrics & Gynecology* 2015;213(4):479-87.
131. Dodd JM, Grivell RM, Obrien CM, et al. Prenatal administration of progestogens for preventing spontaneous preterm birth in women with a multiple pregnancy. *Cochrane Database of Systematic Reviews* 2019(11) doi: 10.1002/14651858.CD012024.pub3
132. Romero R, Conde-Agudelo A, El-Refaie W, et al. Vaginal progesterone decreases preterm birth and neonatal morbidity and mortality in women with a twin gestation and a short cervix: an updated meta-analysis of individual patient data. *Ultrasound in Obstetrics & Gynecology* 2017;49(3):303-14.
133. Sotiriadis A, Papatheodorou S, Makrydimas G. Perinatal outcome in women treated with progesterone for the prevention of preterm birth: a meta-analysis. *Ultrasound in Obstetrics & Gynecology* 2012;40(3):257-66.
134. Lim CE, Ho KK, Cheng NC, et al. Combined oestrogen and progesterone for preventing miscarriage. *Cochrane Database of Systematic Reviews* 2013(9):CD009278.
135. Norman JE, Mackenzie F, Owen P, et al. Progesterone for the prevention of preterm birth in twin pregnancy (STOPPIT): A randomized, double-blind, placebo-controlled study and meta-analysis. *Obstetrical and Gynecological Survey* 2009;64(10):646-48.
136. Likis FE, Edwards DR, Andrews JC, et al. Progestogens for preterm birth prevention: a systematic review and meta-analysis. *Obstetrics & Gynecology* 2012;120(4):897-907.
137. Palacio M, Ronzoni S, Sanchez-Ramos L, et al. Progestogens as Maintenance Treatment in Arrested Preterm Labor: A Systematic Review and Meta-analysis. *Obstetrics & Gynecology* 2016;128(5):989-1000.
138. Prior M, Hibberd R, Asemota N, et al. Inadvertent P-hacking among trials and systematic reviews of the effect of progestogens in pregnancy? A systematic review and meta-analysis. *BJOG: An International Journal of Obstetrics and Gynaecology* 2017;124(7):1008-15.
139. Rode L, Langhoff-Roos J, Andersson C, et al. Systematic review of progesterone for the prevention of preterm birth in singleton pregnancies. *Acta Obstetricia et Gynecologica Scandinavica* 2009;88(11):1180-89.
140. Schmouder VM, Prescott GM, Franco A, et al. The rebirth of progesterone in the prevention of preterm labor. *Annals of Pharmacotherapy* 2013;47(4):527-36.
141. Velez Edwards DR, Likis FE, Andrews JC, et al. Progestogens for preterm birth prevention: a systematic review and meta-analysis by drug route. *Archives of Gynecology & Obstetrics* 2013;287(6):1059-66.
142. Chawanpaiboon S, Laopaiboon M, Lumbiganon P, et al. Terbutaline pump maintenance therapy after threatened preterm labour for reducing adverse neonatal outcomes. *Cochrane Database of Systematic Reviews* 2014(3):CD010800.
143. Crowther CA, Brown J, McKinlay CJ, et al. Magnesium sulphate for preventing preterm birth in threatened preterm labour. *Cochrane Database of Systematic Reviews* 2014(8):CD001060.

For peer review only

144. Gaudet LM, Singh K, Weeks L, et al. Effectiveness of terbutaline pump for the prevention of preterm birth. A systematic review and meta-analysis. *PLoS ONE [Electronic Resource]* 2012;7(2):e31679.
145. Haas D, Caldwell D, Kirkpatrick P, et al. Tocolytic therapy for preterm delivery: systematic review and network meta-analysis (Structured abstract). *Bmj* 2012;344(2):e6226.
146. McNamara HC, Crowther CA, Brown J. Different treatment regimens of magnesium sulphate for tocolysis in women in preterm labour. *Cochrane Database of Systematic Reviews* 2015(12):CD011200.
147. Vogel JP, Nardin JM, Dowswell T, et al. Combination of tocolytic agents for inhibiting preterm labour. *Cochrane Database of Systematic Reviews* 2014(7):CD006169.
148. Dodd JM, Crowther CA, Middleton P. Oral betamimetics for maintenance therapy after threatened preterm labour. *Cochrane Database of Systematic Reviews* 2012;12:CD003927.
149. Flenady V, Reinebrant HE, Liley HG, et al. Oxytocin receptor antagonists for inhibiting preterm labour. *Cochrane Database of Systematic Reviews* 2014(6):CD004452.
150. Giorgino FL, Egan CG. Use of isoxsuprine hydrochloride as a tocolytic agent in the treatment of preterm labour: a systematic review of previous literature. *Arzneimittel-Forschung* 2010;60(7):415-20.
151. Mackeen AD, Seibel-Seamon J, Grimes-Dennis J, et al. Tocolytics for preterm premature rupture of membranes. *Cochrane Database of Systematic Reviews* 2011(10):CD007062.
152. Saccone G, Suhag A, Berghella V. 17-alpha-hydroxyprogesterone caproate for maintenance tocolysis: a systematic review and metaanalysis of randomized trials. *American Journal of Obstetrics & Gynecology* 2015;213(1):16-22.
153. van Vliet EOG, Dijkema GH, Schuit E, et al. Nifedipine maintenance tocolysis and perinatal outcome: an individual participant data meta-analysis. *BJOG: An International Journal of Obstetrics and Gynaecology* 2016;123(11):1753-60.
154. Yamasmit W, Chaithongwongwatthana S, Tolosa JE, et al. Prophylactic oral betamimetics for reducing preterm birth in women with a twin pregnancy. *Cochrane Database of Systematic Reviews* 2012(9):CD004733.
155. Ota E, Mori R, Middleton P, et al. Zinc supplementation for improving pregnancy and infant outcome. *Cochrane Database of Systematic Reviews* 2015(2)
156. Bellows BW, Conlon CM, Higgs ES, et al. A taxonomy and results from a comprehensive review of 28 maternal health voucher programmes. *Journal of health, population, and nutrition* 2013;31(4 Suppl 2):S106.
157. Atal I, Trinquart L, Ravaud P, et al. A mapping of 115,000 randomized trials revealed a mismatch between research effort and health needs in non–high-income regions. *Journal of clinical epidemiology* 2018;98:123-32.
158. Dab W. Commentary on SPHERE (Strengthening Public Health Research in Europe) literature reviews. *European journal of public health* 2007;17(suppl_1):8-9.
159. Shepherd V. Research involving adults lacking capacity to consent: the impact of research regulation on 'evidence biased'medicine. *BMC Medical Ethics* 2016;17(1):1-8.
160. Organization WH. Born too soon: the global action report on preterm birth. 2012

For peer review only

161. Banke-Thomas A, Wong KL, Ayomoh FI, et al. "In cities, it's not far, but it takes long": comparing estimated and replicated travel times to reach life-saving obstetric care in Lagos, Nigeria. *BMJ Global Health* 2021;6(1):e004318.
162. Vogel JP, Chawanpaiboon S, Moller A-B, et al. The global epidemiology of preterm birth. *Best Practice & Research Clinical Obstetrics & Gynaecology* 2018;52:3-12.
163. C S. UKRI Official Development Assistance letter 11 March 2021: UKRI; 2021 [Available from: https://www.ukri.org/our-work/ukri-oda-letter-11-march-2021/ accessed March 23 2021.
164. Organization WH. Millennium development goals. 2008

For peer review only

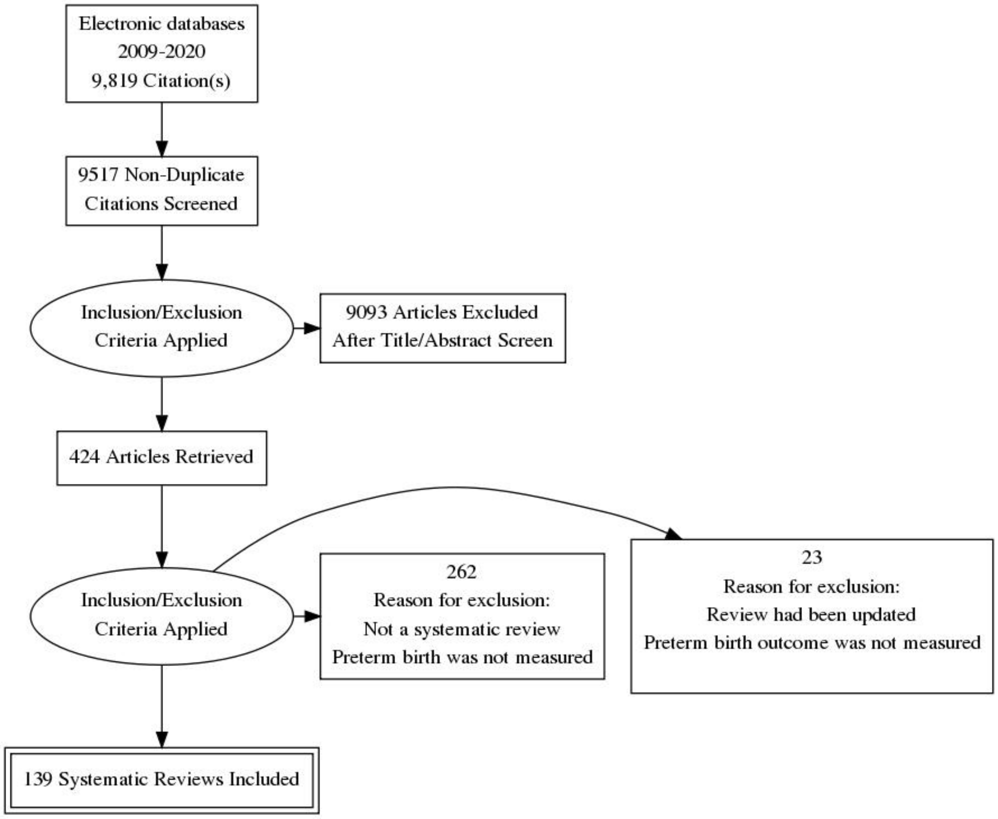

Figure 1: Flow of studies

314x258mm (72 x 72 DPI)

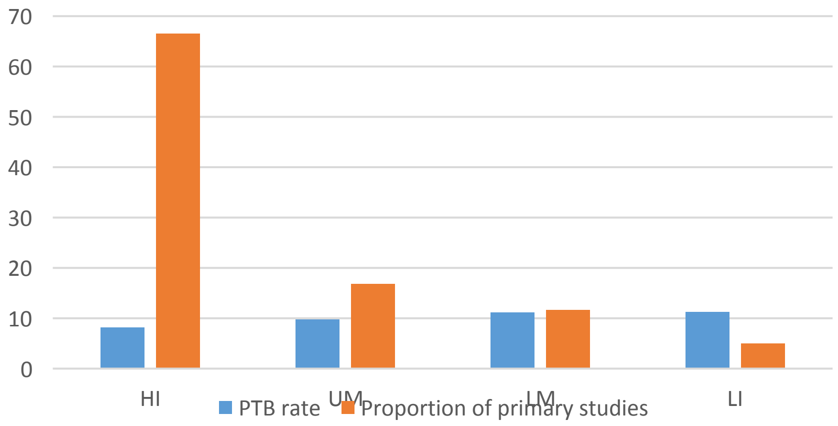

Figure 2: Rates of PTB and proportion of primary studies undertaken in each setting.

Figure 3: Systematic reviews of interventions showing reduction in risk of PTB

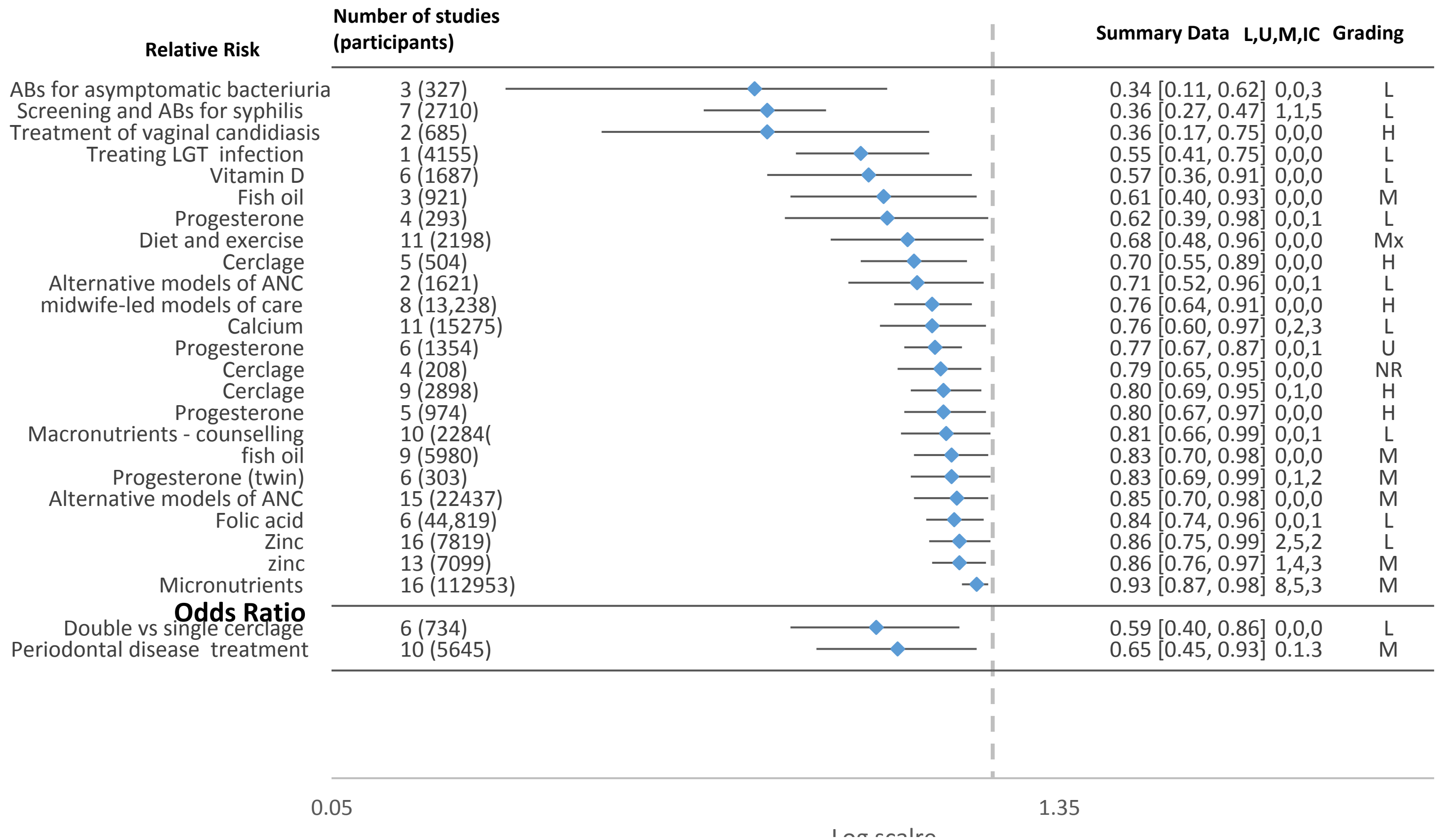

Appendix 1 Search strategy from MEDLINE (via Ovid SP)

The McMaster Reviews Search Filter was utilised (Line 6), available from: https://hiru.mcmaster.ca/hiru/HIRU_Hedges_MEDLINE_Strategies.aspx#Reviews [Accessed 26 March 2021]. The Allen et al review referred to in Line 8 is: Allen J, Gamble J, Stapleton H, Kildea S. Does the way maternity care is provided affect maternal and neonatal outcomes for young women? A review of the research literature. *Women and Birth*. 2012;25(2):54-63.

1 (pre-term birth* or pre term birth* or preterm birth*).tw. (14683)<br>2 exp OBSTETRIC LABOR, PREMATURE/ (23309)<br>3 exp PREMATURE BIRTH/ (11040)<br>4 ((preterm or pre-term or premature) adj3 (birth* or labo?r or deliver*)).tw. (41213)<br>5 or/1-4 (48162)	**Pre-term birth terms**
6 meta analysis.mp,pt. or review.pt. or search:.tw. (2681845)	**Systematic Review (SR) Filter**
7 5 and 6 (8021)	**Pre-term birth terms and SR Filter**
8 (2009 04* or 2009 05* or 2009 06* or 2009 07* or 2009 08* or 2009 09* or 2009 10* or 2009 11* or 2009 12* or 2010* or 2011* or 2012* or 2013* or 2014* or 2015* or 2016* or 2017* or 2018*).dt. (9961452)	**Added to MEDLINE post 1 April 2009 (when Allen et al searches were conducted)**
9 7 and 8 (4156)	**Systematic Reviews post 1 April 2009**
10 limit 9 to english language (3872)	
11 limit 10 to humans (3245)	**Systematic Reviews post 1 April 2009 (English Language and Human only)**

For peer review only

Preferred Reporting Items for Systematic reviews and Meta-Analyses extension for Scoping Reviews (PRISMA-ScR) Checklist

SECTION	ITEM	PRISMA-ScR CHECKLIST ITEM	REPORTED ON PAGE #
**TITLE**			
Title	1	Identify the report as a scoping review.	Yes
**ABSTRACT**			
Structured summary	2	Provide a structured summary that includes (as applicable): background, objectives, eligibility criteria, sources of evidence, charting methods, results, and conclusions that relate to the review questions and objectives.	Yes
**INTRODUCTION**			
Rationale	3	Describe the rationale for the review in the context of what is already known. Explain why the review questions/objectives lend themselves to a scoping review approach.	Yes
Objectives	4	Provide an explicit statement of the questions and objectives being addressed with reference to their key elements (e.g., population or participants, concepts, and context) or other relevant key elements used to conceptualize the review questions and/or objectives.	Yes
**METHODS**			
Protocol and registration	5	Indicate whether a review protocol exists; state if and where it can be accessed (e.g., a Web address); and if available, provide registration information, including the registration number.	unpublished protocol which is available on request to the
Eligibility criteria	6	Specify characteristics of the sources of evidence used as eligibility criteria (e.g., years considered, language, and publication status), and provide a rationale.	Yes
Information sources*	7	Describe all information sources in the search (e.g., databases with dates of coverage and contact with authors to identify additional sources), as well as the date the most recent search was executed.	Yes
Search	8	Present the full electronic search strategy for at least 1 database, including any limits used, such that it could be repeated.	Yes
Selection of sources of evidence†	9	State the process for selecting sources of evidence (i.e., screening and eligibility) included in the scoping review.	Yes
Data charting process‡	10	Describe the methods of charting data from the included sources of evidence (e.g., calibrated forms or forms that have been tested by the team before their use, and whether data charting was done independently or in duplicate) and any processes for obtaining and confirming data from investigators.	Yes
Data items	11	List and define all variables for which data were sought and any assumptions and simplifications made.	Yes
Critical appraisal of individual sources of evidence§	12	If done, provide a rationale for conducting a critical appraisal of included sources of evidence; describe the methods used and how this information was used in any data synthesis (if appropriate).	We did not undertake quality appraisal
Synthesis of results	13	Describe the methods of handling and summarizing the data that were charted.	Yes

St. Michael's
Inspired Care.
Inspiring Science.

For peer review only - http://bmjopen.bmj.com/site/about/guidelines.xhtml

SECTION	ITEM	PRISMA-ScR CHECKLIST ITEM	REPORTED ON PAGE #
**RESULTS**			
Selection of sources of evidence	14	Give numbers of sources of evidence screened, assessed for eligibility, and included in the review, with reasons for exclusions at each stage, ideally using a flow diagram.	Yes
Characteristics of sources of evidence	15	For each source of evidence, present characteristics for which data were charted and provide the citations.	Yes
Critical appraisal within sources of evidence	16	If done, present data on critical appraisal of included sources of evidence (see item 12).	Critical appraisal was not undertaken
Results of individual sources of evidence	17	For each included source of evidence, present the relevant data that were charted that relate to the review questions and objectives.	Yes
Synthesis of results	18	Summarize and/or present the charting results as they relate to the review questions and objectives.	Yes
**DISCUSSION**			
Summary of evidence	19	Summarize the main results (including an overview of concepts, themes, and types of evidence available), link to the review questions and objectives, and consider the relevance to key groups.	Yes
Limitations	20	Discuss the limitations of the scoping review process.	Yes
Conclusions	21	Provide a general interpretation of the results with respect to the review questions and objectives, as well as potential implications and/or next steps.	Yes
**FUNDING**			
Funding	22	Describe sources of funding for the included sources of evidence, as well as sources of funding for the scoping review. Describe the role of the funders of the scoping review.	Yes

JBI = Joanna Briggs Institute; PRISMA-ScR = Preferred Reporting Items for Systematic reviews and Meta-Analyses extension for Scoping Reviews.

* Where *sources of evidence* (see second footnote) are compiled from, such as bibliographic databases, social media platforms, and Web sites.

† A more inclusive/heterogeneous term used to account for the different types of evidence or data sources (e.g., quantitative and/or qualitative research, expert opinion, and policy documents) that may be eligible in a scoping review as opposed to only studies. This is not to be confused with *information sources* (see first footnote).

‡ The frameworks by Arksey and O'Malley (6) and Levac and colleagues (7) and the JBI guidance (4, 5) refer to the process of data extraction in a scoping review as data charting.

§ The process of systematically examining research evidence to assess its validity, results, and relevance before using it to inform a decision. This term is used for items 12 and 19 instead of "risk of bias" (which is more applicable to systematic reviews of interventions) to include and acknowledge the various sources of evidence that may be used in a scoping review (e.g., quantitative and/or qualitative research, expert opinion, and policy document).

From: Tricco AC, Lillie E, Zarin W, O'Brien KK, Colquhoun H, Levac D, et al. PRISMA Extension for Scoping Reviews (PRISMAScR): Checklist and Explanation. Ann Intern Med. 2018;169:467–473. doi: 10.7326/M18-0850.

For peer review only - http://bmjopen.bmj.com/site/about/guidelines.xhtml

BMJ Open

Interventions for the prevention of spontaneous preterm birth: a scoping review of systematic reviews

Journal:	*BMJ Open*
Manuscript ID	bmjopen-2021-052576.R1
Article Type:	Original research
Date Submitted by the Author:	03-Feb-2022
Complete List of Authors:	Campbell, Fiona; University of Sheffield , ScHARR<br>Salam, Shumona; The University of Sheffield, Department of Oncology and Metabolism<br>Sutton , Anthea; The University of Sheffield, ScHARR<br>Jayasooriya, Shamanthi; The University of Sheffield, Academic Unit of Primary Medical Care<br>Mitchell, Caroline; The University of Sheffield, Academic Unit of Primary Medical Care<br>Amabebe, Emmanuel; The University of Sheffield, Department of Oncology and Metabolism<br>Balen, Julie; The University of Sheffield,<br>Gillespie, Bronwen; The University of Sheffield, Department of Oncology and Metabolism<br>Parris, Kerry ; The University of Sheffield, Department of Oncology and Metabolism<br>Soma-Pillay, Priya; University of Pretoria, Steve Biko Academic Hospital<br>Chauke, Lawrence; University of Witwatersrand Department of Obstetrics and Gynaecology<br>Narice, Brenda; The University of Sheffield, Department of Oncology and Metabolism<br>Anumba, Dilichukwu; The University of Sheffield, Department of Oncology and Metabolism
**Primary Subject Heading**:	Evidence based practice
Secondary Subject Heading:	Public health
Keywords:	Maternal medicine < OBSTETRICS, International health services < HEALTH SERVICES ADMINISTRATION & MANAGEMENT, Public health < INFECTIOUS DISEASES, Health policy < HEALTH SERVICES ADMINISTRATION & MANAGEMENT

SCHOLARONE™
Manuscripts

I, the Submitting Author has the right to grant and does grant on behalf of all authors of the Work (as defined in the below author licence), an exclusive licence and/or a non-exclusive licence for contributions from authors who are: i) UK Crown employees; ii) where BMJ has agreed a CC-BY licence shall apply, and/or iii) in accordance with the terms applicable for US Federal Government officers or employees acting as part of their official duties; on a worldwide, perpetual, irrevocable, royalty-free basis to BMJ Publishing Group Ltd ("BMJ") its licensees and where the relevant Journal is co-owned by BMJ to the co-owners of the Journal, to publish the Work in this journal and any other BMJ products and to exploit all rights, as set out in our licence.

The Submitting Author accepts and understands that any supply made under these terms is made by BMJ to the Submitting Author unless you are acting as an employee on behalf of your employer or a postgraduate student of an affiliated institution which is paying any applicable article publishing charge ("APC") for Open Access articles. Where the Submitting Author wishes to make the Work available on an Open Access basis (and intends to pay the relevant APC), the terms of reuse of such Open Access shall be governed by a Creative Commons licence – details of these licences and which Creative Commons licence will apply to this Work are set out in our licence referred to above.

Other than as permitted in any relevant BMJ Author's Self Archiving Policies, I confirm this Work has not been accepted for publication elsewhere, is not being considered for publication elsewhere and does not duplicate material already published. I confirm all authors consent to publication of this Work and authorise the granting of this licence.

For peer review only

Interventions for the prevention of spontaneous preterm birth: a scoping review of systematic reviews

Fiona Campbell[1] Shumona Salam[3], Anthea Sutton[1], Shamanthi Maya Jayasooriya[2], Caroline Mitchell[2] Emmanuel Aamabebe[3] Julie Balen[1] Bronwen Gillespie[3] Kerry Parris[3] Priya Soma-Pillay[5], Lawrence Chauke[4] , Brenda Narice[3] , Dilichukwu O Anumba[3],

[1]School of Health and Related Research, University of Sheffield, Sheffield, UK
[2]Academic Unit of Primary Medical Care, University of Sheffield, Sheffield, UK
[3]Department of Oncology and Metabolism, University of Sheffield
[4]Witwatersrand, Johannesburg, South Africa.
[5]Steve Biko Academic Hospital and University of Pretoria, Pretoria, South Africa

Corresponding Author:
Fiona Campbell
ScHARR, University of Sheffield, Regent Court, 30 Regent Street, S1 4DA,
f.campbell@sheffield.ac.uk
https://orcid.org/0000-0002-4141-8863

Data extracted from included studies is available on request by email from the corresponding author: f.campbell@sheffield.ac.uk

Work Count: 3,473

Keywords: Preterm Birth, Scoping Review, Health services research, Meta-analyses

Contributors: PRIME (Preterm birth Prevention and Management) research program established the study objectives, FC, PSP, LC prepared the protocol, AS designed search strategies, SS, SJ, CM EA, JB, BG, KP, BN collected and analysed or interpreted data. FC prepared manuscript. SS, AS, SJ, CM, EA, JB, BG, KP, PSP, LC, BN, DA edited or read and approved the final manuscript.

Funding: NIHR provided funding for researcher support.

Competing Interests: We declare that there are no competing interests.

Data availability statement: Data extracted from included studies is available on request by email from the corresponding author

Ethical approval: Ethical approval was not required for this study as it drew on existing published research and did not involve human or animal participants.

For peer review only

Abstract

Objectives: Globally, 11% of babies are born preterm each year. Preterm birth (PTB) a leading cause of neonatal death and under-5 mortality and morbidity, with lifelong sequelae in those who survive. PTB disproportionately impacts low- and middle income countries **(**LMICs) where the burden is highest. This scoping review maps the evidence for interventions that reduce the risk of PTB, focusing on the evidence from LMICs and describing how context is considered in evidence synthesis.

Design: We conducted a scoping review, to describe this wide topic area. We searched five electronic databases and contacted experts to identify relevant systematic reviews of interventions to reduce the risk of PTB. We included published systematic reviews that examined the effectiveness of interventions and their effect on reducing the risk of PTB. Data was extracted and is described narratively.

Results: 139 published systematic reviews were included in the review. Interventions were categorised as primary or secondary. The interventions where the results showed a greater effect size and consistency across review findings included treatment of syphilis and vaginal candidiasis, vitamin D supplementation and cervical cerclage. Included in the 139 reviews were 1372 unique primary source studies. 28% primary studies were undertaken in LMIC contexts and only 4.5% undertaken in a low income country (LIC) Only 10.8% of the reviews sought to explore the impact of context on findings, and 19.4% reviews did not report the settings or the primary studies

Conclusion: This scoping review highlights the lack of research evidence derived from contexts where the burden of PTB globally is greatest. The lack of rigour in addressing contextual applicability within systematic review methods is also highlighted. This presents a risk of inappropriate and unsafe recommendations for practice within these contexts. It also highlights a need for primary research, developing and testing interventions LIC settings.

Strengths and Limitations of this Study

- The first review of reviews looking at interventions to reduce the risk of PTB and describe the context in which primary studies were undertaken.
- This is the first review to analyse how context is taken into account in the meta-analyses reported in the reviews.
- This scoping review focused on existing reviews. Primary studies not reported in systematic reviews will therefore have not been included in our analysis.
- We were not able to identify the setting of all primary studies where this was not reported and there is a risk that some studies, which have multiple publications may have been double counted.

For peer review only

BACKGROUND

Preterm birth (PTB) is a global and public health priority. It is defined by the World Health Organization (WHO) as delivery before 37 completed weeks of gestation, with extremely preterm delivery defined as occurring at less than 28 weeks, very preterm delivery occurring between 28 and 32 weeks, and moderate to late preterm delivery occurring from 32 through 36 weeks.[1] It is one of the leading causes of neonatal death and under five mortality and morbidity, with lifelong sequelae.[2] Children born prematurely have increased risks of cognitive problems, such as academic underachievement, behavioural problems and cerebral palsy than those born at full term.[3] They are more likely to experience hospital admission due to infection, particularly during infancy.[4] For parents, the financial, social and emotional effects are devastating.[3]

The global burden of PTB is falling more heavily on countries with fewer resources to manage the medical, social, and economic complexities of caring for premature infants. Globally, there are approximately 15 million live preterm births each year, which is estimated to be about 11% of all deliveries each year, ranging from about 8.7% in northern Europe to 13.4% in North Africa.[5 6] The majority of PTBs occur in Low- or Middle Income countries (LMICs) .[6] The highest PTB rates in 2014 occurred in southeast Asia, south Asia and sub-Saharan Africa. Nine of the 11 countries with the highest rates were in Africa. Furthermore, 60% of all PTBs were estimated to have occurred in sub-Saharan Africa and south Asia accounting for just over nine million of the almost 15 million PTBs that occurred worldwide in 2010 resulting in a PTB rate of 12.8% in those settings.

Patterns of PTB differ between high-income countries and LMICs. However, the differences in these patterns, causes and distribution of PTB is unclear and have not been fully explored. PTB is multifactorial in its aetiology and has distinct biological pathways. The aetiologies differ according to gestational age, ethnicity and characteristics unique to each population. In order to redress the burden of PTB in LMICs, additional insight into the causative and associated factors in these settings is required.

While a number of reviews and overviews of reviews of interventions to reduce the risk of PTB have been undertaken[7-10], none have explored how many of the primary studies included in these reviews were undertaken in LMIC contexts. It is clear that some interventions that are effective in HIC (high income country) contexts but may be harmful in LMIC settings, such as the use of antenatal corticosteroids[11] and cerclage.[12] It is also possible that treatments effective in HIC contexts may be even more beneficial or appropriate in LMIC contexts, such as nutritional supplements, interventions to increase birth spacing, or interventions to improve the accuracy of measuring gestational age.

We have undertaken a broad scoping review of systematic reviews on interventions to reduce the risk of PTB identifying primary studies undertaken in LMICs. This will allow us to identify potential areas for further synthesis of the evidence and also to identify gaps in the research in order to direct future primary research.

For peer review only

Review objectives

1. To identify systematic reviews that have sought to explore the effectiveness, safety and acceptability of interventions to prevent PTB.
2. To map research evidence to global settings to identify the geographical and economic contexts in which evidence is derived.
3. To identify where gaps in the research base exist (for real world, effectiveness, pragmatic studies) in LMIC contexts to inform future research and to generate research priorities.
4. To describe the methods used in meta-analysis to take into account geographical and regional differences in PTB.

METHODS

We used a scoping review methodology[13] to describe the existing evidence (systematic reviews) available across primary and secondary interventions to prevent PTB,published between 2009 and 2019. Systematic scoping draws upon methods described by Arksey & O'Malley (2005)[14] for scoping reviews: "[…a form of knowledge synthesis that addresses an exploratory research question aimed at scoping key concepts, types of evidence, and gaps in research related to a defined area or field by systematically searching, selecting, and synthesizing existing knowledge".[14] The approach enabled us to highlight the evidence gap and to assist with simultaneously undertaking a research prioritisation exercise and guideline development, as well as to inform a broader programme of research that aimed to develop effective postnatal interventions to mitigate PTB in LMIC settings. It also enabled us to generate a mega-map, an interactive table supported on our project website and designed as a visual tool to identify research gaps and facilitate ready access to relevant evidence. https://www.primeglobalhealth.co.uk/evidence-map-2-7-2020.html.

Identifying relevant studies

Relevant systematic reviews were identified by systematic searches in the following electronic databases: Ovid MEDLINE, Cochrane Database of Systematic Reviews (CDSR), PsycINFO via Ovid, EMBASE via Ovid and CINAHL via EBSCO. Each database was searched using the database thesaurus and the key word/free text method with terms relating to preterm birth combined with a systematic reviews filter. The search strategy, incorporated the following limitations: articles written in English, and Human studies only from April 2009 to July 2020. Relevant systematic reviews were identified by systematic searches in the following electronic databases: MEDLINE, The Cochrane Library, PsycINFO, EMBASE and CINAHL. Each database was searched using the database thesaurus and the key word/free text method. The search strategy, incorporated the following limitations: articles written in English, and Human studies only from April 2009 to July 2020. The date limit was selected due to the existence of a previous review for which the studies were conducted in April 2009.[15] Full search strategies can be found in Appendix 1.

For peer review only

We began with a framework of interventions identified by two existing reviews[7,8] as these were broad in their focus and encompassed a range of interventions. Any new intervention types identified during the screening process were then added to the map.

The process of study selection was based on inclusion and exclusion criteria as described in Table 1. After removal of duplicates and irrelevant studies, based on the titles and abstracts, all potentially relevant reviews were read in full. Citations were screen by two reviewers (FC and one of the following team members SS, SJ, EA, JB, BG, BN, KP) independently and differences were resolved by discussion.

Table 1 Inclusion/ exclusion criteria based on PICOS

Population

- Pregnant women at less than 37 completed weeks gestation without signs of threatened preterm labour or premature rupture of membranes (PPROM).
- Excluded reviews where the study population was defined by co-morbidities.

Intervention

- All interventions deliverable during pregnancy to prevent spontaneous preterm birth, (these included clinical, behavioural and nutritional interventions and health systems and policy interventions).
- All interventions assessed the risk of preterm birth.
- Excluded interventions given to pregnant women to improve neonatal outcomes.

Comparators

- We included any comparator, including placebo or alternative treatments.

Outcomes

- We included reviews which focused to PTB as an outcome.
- Where it is reported, we state how many of the primary studies measured PTB as an outcome and the resulting data used in the synthesis.

Study design

1. Systematic reviews published between April 2009-July 2020, of studies that have evaluated interventions to prevent PTB, or that measured PTB as a relevant outcome.

Outcomes

1. Preterm birth (<28, <34, <37 weeks gestation) .
2. We recorded neonatal outcomes and adverse outcomes if reported within the review.

For peer review only

Data extraction and coding

Data were extracted using an agreed and piloted template and coded in Excel by two reviewers working independently (FC and one of the following team members SS, SJ, EA, JB, BG, BN, KP) differences were resolved by discussion. The following data categories were extracted: number of included studies, review PICO, setting of primary studies, and any analysis that took into account study setting or population characteristics, PTB outcomes, assessment of adverse effects and recommendations for practice and research. Preterm birth rates in LICs, LMCs, UMCs and HICs settings were drawn from data published in a rigorous

review of national civil registration and vital statistics to determine global, regional and national estimates of levels of preterm birth.[6]

Where reported information allowed, we used the World Bank categories to identify the categories of all country settings identified in the reviews.[161] .

The population, interventions, comparators, outcomes and reviewer conclusions for future research were tabulated and described narratively. The country or countries of the included primary studies were noted, and the methods used in the review for analyses of data from different settings was also recorded and described. We did not contact review authors for missing data.

Patient and Public Involvement

This review was undertaken as part of a larger program of research in preterm birth (NIHR Global Health under grant (17/63/26)). The program is informed by key stakeholders and a PPI advisory group comprising of representatives from Sheffield, Bangladesh, and South Africa. The design and questions for the review were informed by consultation with these groups.

Results

Our search identified 3,133 citations which were screened by two reviewers. A third reviewer was also involved where there was a lack of consensus or uncertainty regarding inclusion. Following screening, 424 full text papers were retrieved for data extraction. At data extraction a further 285 were excluded. The process of identifying the included reviews is summarised in Figure 1.

We included 139 reviews which addressed a range of primary and secondary interventions and measured the effectiveness of the intervention in reducing the risk of PTB. These are summarised in Table 2 There was a considerable variation in the number of included studies in the reviews for each intervention, reflecting differing research questions objectives (therefore different PICOs) and search strategies.

[1] Low-income economies are defined as those with a GNI per capita, calculated using the World Bank Atlas method, of $995 or less; lower middle-income economies are those with a GNI per capita between $996 and $3,895; upper middle-income economies are those with a GNI per capita between $3,896 and $12,055; high-income economies are those with a GNI per capita of $12,056 or more.

For peer review only

Table 2 Summary of included systematic reviews and settings of primary studies included in the review

Interventions	Number of reviews	Number of primary studies	country NR	country of primary study					studies where setting NK
				LI	LM	UM	HI	mixed	
**Primary prevention interventions**:									
**Health Systems**									
Models of antenatal care delivery (group/specialised)[17-27]	11	68	2	0	2	2	64	0	0
Midwifery led care[28]	1	15	0	0	0	0	15	0	0
Improving ANC coverage[29]	1	34	0	10	15	5	0	0	0
**Health behaviours**									
Smoking cessation[30 31]	2	111	0	0	0	1	110	0	0
Weight management[32-37]	6	70	1	0	2	8	60	0	0
**Nutritional interventions**									
Macronutrient supplements[38 39]	2	34	0	3	9	10	8	4	0
Micronutrient supplements[24-55]	33	481	2	29	82	122	214	6	9
Vitamin D[40-45]	6	75							
Vitamin A[46 47]	2	24							
Vitamin E, C, E and C[48-50]	3	67							
Iron, folic acid, iron and folic acid[51-58]	8	182							
Fish oil[59-63]	5	38							

For peer review only

Zinc[64 65]	2	25							
Calcium[66 67]	2	27							
Iodine[68]	2	14							
Multiple micronutrients[69-71]	3	29							
**Screening and treatment of periodontal disease[72-83]**	12	46	0	0	3	7	36	0	0
**Screening and prevention/treatment of infection**	14	91	2	2	2	6	79	0	2
Asymptomatic bacteriuria[84-87]	4								
Screening and antibiotics for syphilis[88]	1								
Influenza vaccine[89 90]	2								
Lower genital tract infection[91]	1								
UTI[92 93]	2								
Vaginal candidiasis[94]	1								
Nonspecific infection[95 96]	2								
Malaria[97-99]	3	17	0	8	7	2	2	0	0
**Secondary prevention interventions**:									
Cerclage[100-117]	18	123	10	0	7	11	42		51
Bed rest[118-120]	3	40	1	4	0	0	36	0	0
Cervical pessary[121-126]	6	16	0	0	0	1	14	1	0
Progesterone[127-142]	16	59	5	1	7	8	28	4	11
Tocolytics[143-155]	11	167	3	1	0	13	68	0	84

ANC: antenatal care, NK: not known, NR: not reported, LI: low income, LM: low middle, UM: upper middle, HI: high income, UTI: urinary tract infection.

For peer review only

Context of primary studies

A total of 1372 primary studies were included across all of the 139 reviews Not all of these studies will have been measuring PTB as an outcome but were included within the review which may have been measuring a range of maternal outcomes including PTB. The largest number of primary studies were those evaluating micronutrient supplements (n=481) and tocolytics (n=167). A total of 113 of the reviews described the country in which the primary studies were undertaken and so this data was known for 1288 (93.9%) of the 1372 included primary studies. Of these, 390 (30.3%) were undertaken in LMICs, fifteen primary studies were multicentre and included data gathered from LMIC and HIC settings, though only three of these studies included LICs. Of the studies undertaken in LMICs, a majority (n=255;) examined the effects of nutritional supplements. Excluding nutritional intervention studies, the proportion of LMIC-based primary studies of interventions to reduce PTB accounts for only (n=135) 10.5% of the included studies where settings are known..

Of the total number of primary studies undertaken in LMIC contexts, those studies undertaken in LIC settings represented a very small proportion of included studies. Participants from LICs were represented in only 4.5% (n=58) of the total number of studies, and if the nutritional intervention studies are excluded, they account for only 2.5% (n=32) of the studies evaluating interventions. Of those primary studies that were undertaken in LMIC settings the numbers within each country category differed significantly. The proportion of the studies that are undertaken in LIC, LMC and UMC were 14.9% (n=58), 34.8% (n=136) and 50.2% (n=196) respectively. There are only single trials that have evaluated the impact of progesterone, tocolytics and interventions to increase calorie intake in LIC settings. There are no trials that have evaluated smoking cessation, preventing excessive weight gain, prevention and treatment of periodontal disease, flu vaccine and cervical pessaries. The number of trials in each of the country categories within each intervention type are shown in Table 2.

When this data is compared alongside data that shows the prevalence of PTB globally it is clear that there is an inverse pattern in the distribution of the data (Figure 2).

Figure 2: Rates of PTB and proportion of primary studies undertaken in each setting.

The effectiveness of interventions

The effectiveness of interventions in reducing the risk of PTB was variable with no intervention showing consistent effectiveness across the included reviews. Although interpretation of this data is limited by the lack of quality appraisal of the included reviews, and therefore should be viewed with caution. Overall, the scoping review demonstrates considerable inconsistency of results of interventions. Of the 139 reviews, 28 reported a reduction in PTB in intervention versus a control, 80% (n=111) of the reviews found that the intervention had no impact in reducing the risk of PTB. The summary result (relative risk and odds ratio are shown in Figure 3). The results show the reduction in PTB less than 37 weeks gestation. In three reviews the intervention was not statistically significant at 37 weeks but was reported as statistically significant at 34 weeks[108], 35 weeks[133] and 36 weeks[127]. Two reviews reported a positive effect of the intervention in reducing risk of preterm birth but reported the outcome on a continuous measure. These included the effectiveness of macronutrient supplements[33] (SMD -0.19 (95% CI -0.34 to -0.04)) and cerclage (mean difference 95% CI 33.98 days

For peer review only - http://bmjopen.bmj.com/site/about/guidelines.xhtml

(17.88 to 50.08))[105]. The interventions reporting binary outcomes which appear to have the greatest effect (RR = 0.2-0.4) in reducing PTB are: antibiotics for asymptomatic bacteriuria[85] (RR = 0.34 (95% CI 0.11 to 0.62), the screening and treatment of syphilis[88] (RR = 0.36 (95% CI 0.27-0.47), and treatment of vaginal candidiasis[94] (RR = 0.36, (95% CI 0.17 to 0.75). Interventions with moderate effects (RR = 0.4-0.6) included treating lower genital tract infection[91] and vitamin D supplements.[45] Four of the reviews (Figure 2) with a positive effect of the intervention considered that the strength of evidence supporting the finding could be considered high and the finding reliable. None of these reviews included studies conducted in LIC settings, and only one included one study in a LMIC.

Figure 3 Summary results of systematic reviews of interventions showing reduction in risk of PTB

ANC: antenatal care, RR: relative risk, OR: odds ratio, LGT: lower genital tract, L,M,IC: low, low middle, upper middle income countries.

For peer review only

Dealing with context and generalisability within evidence synthesis

The authors of the included reviews used different approaches to dealing with the contextual variation when pooling data from primary studies, which was either to ignore, document, explore or control for differences. Twenty-seven reviews (23.8%) did not describe the setting of the primary study, ignoring variation in outcomes that may arise as a result of these differences. This occurred most frequently in reviews of cervical cerclage (see Table 2). The majority of the included reviews 86 (76.1%) documented the country in which the primary study was carried out either within the text, tables of study characteristics or in accompanying appendices, but this was not considered further in terms of its implications for the findings, or application for future practice or research.

Eight reviews[29 38 39 43 45 64 98 156] sought to explore the impact of geographical and economic context by undertaking a subgroup analysis comparing trials conducted in low income settings with those in high income settings or regression analysis with geographical regions as covariates (Africa, Americas, Southeast Asia, Europe, Eastern Mediterranean, Western Pacific). In addition, one study[156] listed the country instead of the author name on the forest plot allowing ready visualisation of differences across settings. Nine reviews[30 41 46 49 50 54 66 67 70] undertook subgroup analysis based on features of the population that might vary across settings and influence the effectiveness of the intervention, such as baseline nutritional status of the mother. One review[71] exploring multiple micronutrient supplementation controlled for settings by limiting the review to include only those studies undertaken in LMIC contexts. Four reviews[71 101 127 133] undertook an IPD (individual patient data) analysis, allowing subgroup analyses about differences in effect more easily than with aggregate data. This approach allowed comparison between effects for women recruited and receiving the intervention in different settings, effect sizes in each country could also be shown in the analyses.

DISCUSSION

This scoping review has revealed an inverse pattern of research, with only 30.3% of published research included in systematic reviews of interventions reporting PTB outcomes carried out in LMIC settings, and only 4.5% was conducted in the poorest countries in the world where the burden of PTB is greatest. The distribution of types of intervention tested and evaluated in these settings is not even across interventions, but is largely focused on very context specific interventions (prevention of malarial infection) and nutritional supplementation. Similar patterns of a mismatch between research effort and health needs in non- high income regions have been identified across a broad range of diseases.[157 158] It has also been previously reported that primary research often fails to capture those with the greatest health care needs such as vulnerable populations.[159 160]

This review has also revealed a limited approach in evidence synthesis to explore the applicability of findings across geographical settings and to draw attention to these gaps with a resultant risk that interventions shown to be effective in HI settings may not translate to LIC settings and may indeed have adverse effects when applied to LIC settings. Likewise, the focus of research in HIC settings means that interventions that may have greater benefit in LIC settings – where the problem is greatest – remain untested or replicated with larger numbers of participants. Adolescent pregnancy and short inter pregnancy intervals, both of which are more common in LMICs, have been highlighted as important risk factors for PTB[161] yet there is a lack of data on interventions to address these and their effectiveness in reducing the risk of PTB.

For peer review only

The lack of robust evidence to inform both the primary and secondary prevention of PTB in LIC settings, where the prevalence of PTB is highest presents challenges for developing appropriate and contextually relevant clinical guidance. . The factors that mean findings cannot be generalised from high resource settings to low and middle resource settings are multiple and will differ across interventions. Ethnicity, poverty, gender dynamics, pollution, temperature, climate, diet, access to health care, educational status, employment conditions are all examples of factors that might play a role in these differences. Improved understanding of the etiopathogenesis of PTB is also necessary for defining an accurate model of risk prediction and would help in understanding what factors in local settings increase risk and facilitate the development of an accurate model of risk prediction. [162]

Two recent overviews of reviews[9 10] also found that few interventions are effective in PTB prevention. The following interventions were identified in these reviews as showing positive or possible benefit: lifestyle and behavioural changes (including diet and exercise); nutritional supplements (including calcium, zinc and vitamin D supplementation); nutritional education; and screening for lower genital tract infections. Positive effects of secondary interventions were found for low dose aspirin among women at risk of preeclampsia; clindamycin for treatment of bacterial vaginosis; treatment of vaginal candidiasis; progesterone in women with prior spontaneous PTB and in those with short mid-trimester cervical length; L-arginine in women at risk for preeclampsia; levothyroxine among women with thyroid disease; calcium supplementation in women at risk of hypertensive disorders; smoking cessation; cervical length screening in women with history of PTB with placement of cerclage in those with short cervix; cervical pessary in singleton gestations with short cervix; and treatment of periodontal disease. Our review findings were in concordance, although, in addition, we identified screening and antibiotic treatment for syphilis, and positive effects of fish oil supplements. In most instances the trials were small and authors recommended larger well-designed RCTs. The lack of consistency across review findings for interventions also merits more exploration. Compromised methodological rigour can inflate trial findings by 30% to 50%.[163 164] Some of the differences in our review findings reflect some differences in the included reviews.

The interventions identified in this review, and those of Matei et al (2019)[9] and Medley et al (2019)[10] informing guideline development, clinical practice and policy decision making have been little tested in LMIC settings. In those interventions where there is more consistency in review findings such as cervical cerclage, there are no studies that have been conducted in low income settings and over half of the reviews did not report or consider settings in their analyses.

This scoping review has shown that many authors of systematic reviews fail to use design and statistical approaches that adequately address contextual variations between the included source studies and imperfectly represent 'real world' conditions within the target context (Higgins et al 2019). While those reviews that sought to take into account LMIC contexts were unable to conduct the analyses due to a lack of data, they nonetheless were able to highlight the gaps in research, for example the lack of studies in vitamin D undertaken in Africa.[43]

The Preferred Reporting Items for Systematic reviews and Meta-analyses (PRISMA) reporting standards reference 'context' in terms of the circumstances requiring the review itself, rather than referencing the contexts of studies included in the review.[165] The PRISMA extension for Complex Interventions includes the elements of 'time' and 'setting'.[166] However, grouping LMIC data, or even LI data may still be too broad. Even within the categories of LIC there is considerable diversity that

For peer review only

may impact on how an intervention works and within countries there may also be considerable diversity between the wealthiest and poorest groups. For example, the time taken to reach comprehensive emergency obstetric care facilities in low resource settings is often underestimated and for most women is likely to be 120 minutes of travel time.[167] Context cannot be standardised, it will vary from review to review, as different interventions and different populations are considered. 'Context' and the factors that might influence the efficacy, uptake, acceptability, appropriateness, accessibility and availability of an intervention requires a good understanding of the aetiology and mechanisms by which risk factors interact with environmental, microbial, socio-political and health system variations across settings.[168]

It must be acknowledged that there are significant barriers to undertaking research in many settings across the globe. These include very practical challenges such as a lack of access to high quality data and the challenges of estimating gestational age.[169] Recent changes to global health funding arena include a very large proportion being spent on the pandemic as well as government reductions, e.g. in the UK [170]. These reductions in funding will undermine what has been a growth in research in LMIC settings and will impede efforts to address the imbalances highlighted in this scoping review.

A number of limitations exist in this scoping review. We have not sought to identify the setting of primary studies where this is not reported in the systematic review. We have also not limited our analysis to studies within the reviews that only contributed findings to the risk of PTB. Most reviews explored several maternal and infant outcomes. Therefore, in this scoping review, included primary studies may not have contained PTB outcome data. We limited our scoping review to exploring evidence within systematic reviews as these are key sources of evidence to inform guideline development and policy decision making. It is possible that further primary studies have been published but are not included in this analysis. Nevertheless, it gives an indication of the distribution of research being undertaken in the poorest regions of the world that address PTB.

CONCLUSION

Only 4.5% of primary research to examine the effectiveness of interventions to reduce the risk of PTB is carried out in settings where the burden is greatest. No interventions which reduce the risk of PTB, judged to be supported by strong evidence, include studies undertaken in low resource settings. In the synthesis of studies, current methods often fail to address the contextual variation and consider the applicability of findings in low resource, high burden settings. This has implications for supporting policy making, and development of contextually relevant clinical guidelines. While methods can be undertaken to improve approaches to evidence synthesis, they cannot compensate for the lack of primary research in low resource settings. This is critical if global health inequalities are to be addressed and millennium development goals171 to reduce under-five mortality are to be achieved. Funding and supporting research in LMICs would have a three-fold benefit; firstly, if the prevalence of the disease is higher it is easier to reach statistical significance for efficacy or inefficacy of each tested intervention. Secondly, it would address the knowledge gap highlighted in this review and finally – and most importantly – the implementation of effective interventions would have the potential for greater public health impact where the risks are greater, more prevalent and outcomes more severe.

For peer review only

References

1. WHO. WHO: recommended definitions, terminology and format for statistical tables related to the perinatal period and use of a new certificate for cause of perinatal deaths. Modifications recommended by FIGO as amended October 14, 1976. . *Acta Obstet Gynecol Scand*

1977;1977; 56: 247–53.

2. Liu L, Oza S, Hogan D, et al. Global, regional, and national causes of child mortality in 2000–13, with projections to inform post-2015 priorities: an updated systematic analysis. *The Lancet* 2015;385(9966):430-40.
3. Brydges CR, Landes JK, Reid CL, et al. Cognitive outcomes in children and adolescents born very preterm: a meta-analysis. *Developmental Medicine & Child Neurology* 2018;60(5):452-68.
4. Coathup V, Boyle E, Carson C, et al. Gestational age and hospital admissions during childhood: population based, record linkage study in England (TIGAR study). *bmj* 2020;371
5. Howson CP, Kinney MV, McDougall L, et al. Born too soon: preterm birth matters. *Reproductive health* 2013;10(1):1-9.
6. Chawanpaiboon S, Vogel JP, Moller A-B, et al. Global, regional, and national estimates of levels of preterm birth in 2014: a systematic review and modelling analysis. *The Lancet Global Health* 2019;7(1):e37-e46.
7. Barros FC, Bhutta ZA, Batra M, et al. Global report on preterm birth and stillbirth (3 of 7): evidence for effectiveness of interventions. *BMC pregnancy and childbirth* 2010;10(1):1-36.
8. Bhutta ZA, Das JK, Bahl R, et al. Can available interventions end preventable deaths in mothers, newborn babies, and stillbirths, and at what cost? *The Lancet* 2014;384(9940):347-70.
9. Matei A, Saccone G, Vogel JP, et al. Primary and secondary prevention of preterm birth: a review of systematic reviews and ongoing randomized controlled trials. *European Journal of Obstetrics & Gynecology and Reproductive Biology* 2019;236:224-39.
10. Medley N, Vogel JP, Care A, et al. Interventions during pregnancy to prevent preterm birth: an overview of Cochrane systematic reviews. *Cochrane Database of Systematic Reviews* 2018(11)
11. Opiyo N, Stones W. Corticosteroids for preterm deliveries: missing evidence. *Cochrane Database Syst Rev* 2017;5:ED000121.
12. Egwuatu V. Complications of cervical cerclage in Igbo women. *Journal of the National Medical Association* 1986;78(3):245.
13. White H, Albers B, Gaarder M, et al. Guidance for producing a Campbell evidence and gap map. *Campbell Systematic Reviews* 2020;16(4):e1125.
14. Arksey H, O'Malley L. Scoping studies: towards a methodological framework. *International journal of social research methodology* 2005;8(1):19-32.
15. Allen F, Gray R, Oakley L, et al. Technical guide to the infant mortality evidence map: systematic reviews of interventions targeting major potentially modifiable risk factors for infant mortality. 2009

For peer review only

16. Bank TW. world Bank country and Lending Groups 2021 [Available from: https://datahelpdesk.worldbank.org/knowledgebase/articles/906519 accessed 18/03/2021.
17. Allen J, Gamble J, Stapleton H, et al. Does the way maternity care is provided affect maternal and neonatal outcomes for young women? A review of the research literature (Structured abstract). *Women and Birth* 2012;25(2):54-63.
18. Catling CJ, Medley N, Foureur M, et al. Group versus conventional antenatal care for women. *Cochrane Database of Systematic Reviews* 2015(2):CD007622.
19. Dodd JM, Dowswell T, Crowther CA. Specialised antenatal clinics for women with a multiple pregnancy for improving maternal and infant outcomes. *Cochrane Database of Systematic Reviews* 2015(11) doi: 10.1002/14651858.CD005300.pub4
20. Dowswell T, Carroli G, Duley L, et al. Alternative versus standard packages of antenatal care for low-risk pregnancy. *Cochrane Database of Systematic Reviews* 2015(7):CD000934.
21. Dowswell T, Middleton P, Weeks A. Antenatal day care units versus hospital admission for women with complicated pregnancy. *Cochrane Database of Systematic Reviews* 2009(4) doi: 10.1002/14651858.CD001803.pub2
22. Fernandez Turienzo C, Sandall J, Peacock JL. Models of antenatal care to reduce and prevent preterm birth: a systematic review and meta-analysis. *BMJ Open* 2016;6(1):e009044.
23. Lathrop B. A systematic review comparing group prenatal care to traditional prenatal care. *Nursing for Women's Health* 2013;17(2):118-30.
24. Malouf R, Redshaw M. Specialist antenatal clinics for women at high risk of preterm birth: a systematic review of qualitative and quantitative research. *BMC Pregnancy & Childbirth* 2017;17(1):51.
25. Ruiz-Mirazo E, Lopez-Yarto M, McDonald SD. Group prenatal care versus individual prenatal care: a systematic review and meta-analyses. *Journal of Obstetrics & Gynaecology Canada: JOGC* 2012;34(3):223-29.
26. Sheeder J, Weber Yorga K, Kabir-Greher K. A review of prenatal group care literature: the need for a structured theoretical framework and systematic evaluation. *Maternal & Child Health Journal* 2012;16(1):177-87.
27. Whitworth M, Quenby S, Cockerill RO, et al. Specialised antenatal clinics for women with a pregnancy at high risk of preterm birth (excluding multiple pregnancy) to improve maternal and infant outcomes. *Cochrane Database of Systematic Reviews* 2011(9):CD006760.
28. Sandall J, Soltani H, Gates S, et al. Midwife-led continuity models versus other models of care for childbearing women. *Cochrane Database of Systematic Reviews* 2013(8):CD004667.
29. Mbuagbaw L, Medley N, Darzi AJ, et al. Health system and community level interventions for improving antenatal care coverage and health outcomes. *Cochrane Database of Systematic Reviews* 2015(12) doi: 10.1002/14651858.CD010994.pub2
30. Chamberlain C, O'Mara-Eves A, Oliver S, et al. Psychosocial interventions for supporting women to stop smoking in pregnancy. *Cochrane Database of Systematic Reviews* 2013(10):CD001055.
31. Coleman T, Chamberlain C, Davey MA, et al. Pharmacological interventions for promoting smoking cessation during pregnancy. *Cochrane Database of Systematic Reviews* 2012(9):CD010078.

For peer review only

32. Dodd J, Grivell R, Crowther C, et al. Antenatal interventions for overweight or obese pregnant women: a systematic review of randomised trials (Structured abstract). *BJOG An International Journal of Obstetrics and Gynaecology* 2010;117(11):1316-26.
33. Gresham E, Bisquera A, Byles JE, et al. Effects of dietary interventions on pregnancy outcomes: a systematic review and meta-analysis. *Maternal & Child Nutrition* 2016;12(1):5-23.
34. Muktabhant B, Lawrie TA, Lumbiganon P, et al. Diet or exercise, or both, for preventing excessive weight gain in pregnancy. *Cochrane Database of Systematic Reviews* 2015(6):CD007145.
35. Shepherd E, Gomersall JC, Tieu J, et al. Combined diet and exercise interventions for preventing gestational diabetes mellitus. *Cochrane Database of Systematic Reviews* 2017(11) doi: 10.1002/14651858.CD010443.pub3
36. Tanentsapf I, Heitmann BL, Adegboye AR. Systematic review of clinical trials on dietary interventions to prevent excessive weight gain during pregnancy among normal weight, overweight and obese women. *BMC Pregnancy & Childbirth* 2011;11:81.
37. Thangaratinam S, Rogozinska E, Jolly K, et al. Interventions to reduce or prevent obesity in pregnant women: a systematic review. *Health Technology Assessment (Winchester, England)* 2012;16(31):iii-iv, 1-191.
38. Girard AW, Olude O. Nutrition education and counselling provided during pregnancy: effects on maternal, neonatal and child health outcomes. *Paediatric and perinatal epidemiology* 2012;26:191-204.
39. Ota E, Hori H, Mori R, et al. Antenatal dietary education and supplementation to increase energy and protein intake. *Cochrane Database of Systematic Reviews* 2015(6):CD000032.
40. Bi WG, Nuyt AM, Weiler H, et al. Association Between Vitamin D Supplementation During Pregnancy and Offspring Growth, Morbidity, and Mortality: A Systematic Review and Meta-analysis. *JAMA Pediatrics* 2018;172(7):635-45.
41. Palacios C, De-Regil LM, Lombardo LK, et al. Vitamin D supplementation during pregnancy: Updated meta-analysis on maternal outcomes. *Journal of Steroid Biochemistry & Molecular Biology* 2016;164:148-55.
42. Perez-Lopez FR, Pasupuleti V, Mezones-Holguin E, et al. Effect of vitamin D supplementation during pregnancy on maternal and neonatal outcomes: a systematic review and meta-analysis of randomized controlled trials. *Fertility & Sterility* 2015;103(5):1278-88.e74.
43. Roth DE, Leung M, Mesfin E, et al. Vitamin D supplementation during pregnancy: state of the evidence from a systematic review of randomised trials. *Bmj* 2017;359:j5237.
44. Thorne-Lyman A, Fawzi WW. Vitamin D during pregnancy and maternal, neonatal and infant health outcomes: a systematic review and meta-analysis. *Paediatric and Perinatal Epidemiology* 2012;26 Suppl 1:75-90.
45. Zhou SS, Tao YH, Huang K, et al. Vitamin D and risk of preterm birth: Up-to-date meta-analysis of randomized controlled trials and observational studies. *Journal of Obstetrics & Gynaecology Research* 2017;43(2):247-56.
46. McCauley ME, van den Broek N, Dou L, et al. Vitamin A supplementation during pregnancy for maternal and newborn outcomes. *Cochrane Database of Systematic Reviews* 2015(10):CD008666.

For peer review only

47. Thorne-Lyman AL, Fawzi WW. Vitamin A and carotenoids during pregnancy and maternal, neonatal and infant health outcomes: a systematic review and meta-analysis. *Paediatric and Perinatal Epidemiology* 2012;26 Suppl 1:36-54.
48. Rahimi R, Nikfar S, Rezaie A, et al. A meta-analysis on the efficacy and safety of combined vitamin C and E supplementation in preeclamptic women. *Hypertension in Pregnancy* 2009;28(4):417-34.
49. Rumbold A, Ota E, Hori H, et al. Vitamin E supplementation in pregnancy. *Cochrane Database of Systematic Reviews* 2015(9):CD004069.
50. Rumbold A, Ota E, Nagata C, et al. Vitamin C supplementation in pregnancy. *Cochrane Database of Systematic Reviews* 2015(9):CD004072.
51. Lassi ZS, Salam RA, Haider BA, et al. Folic acid supplementation during pregnancy for maternal health and pregnancy outcomes. *Cochrane Database of Systematic Reviews* 2013(3):CD006896.
52. Mantovani E, Filippini F, Bortolus R, et al. Folic acid supplementation and preterm birth: results from observational studies. *BioMed Research International* 2014;2014:481914.
53. Pena-Rosas JP, De-Regil LM, Dowswell T, et al. Intermittent oral iron supplementation during pregnancy. *Cochrane Database of Systematic Reviews* 2012(7):CD009997.
54. Peña-Rosas JP, De-Regil LM, Garcia-Casal MN, et al. Daily oral iron supplementation during pregnancy. *Cochrane Database of Systematic Reviews* 2015(7) doi: 10.1002/14651858.CD004736.pub5
55. Pena-Rosas JP, Viteri FE. Effects and safety of preventive oral iron or iron+folic acid supplementation for women during pregnancy. *Cochrane Database of Systematic Reviews* 2009(4):CD004736.
56. Saccone G, Berghella V. Folic acid supplementation in pregnancy to prevent preterm birth: a systematic review and meta-analysis of randomized controlled trials. *European Journal of Obstetrics, Gynecology, & Reproductive Biology* 2016;199:76-81.
57. Zhang Q, Wang Y, Xin X, et al. Effect of folic acid supplementation on preterm delivery and small for gestational age births: A systematic review and meta-analysis. *Reproductive Toxicology* 2017;67:35-41.
58. Imdad A, Bhutta Z. Routine iron/folate supplementation during pregnancy: effect on maternal anaemia and birth outcomes (Structured abstract). *Paediatric and Perinatal Epidemiology* 2012;26(Supplement 1):168-77.
59. Chen B, Ji X, Zhang L, et al. Fish oil supplementation improves pregnancy outcomes and size of the newborn: a meta-analysis of 21 randomized controlled trials. *Journal of Maternal-Fetal & Neonatal Medicine* 2016;29(12):2017-27.
60. Kar S, Wong M, Rogozinska E, et al. Effects of omega-3 fatty acids in prevention of early preterm delivery: a systematic review and meta-analysis of randomized studies. *European Journal of Obstetrics, Gynecology, & Reproductive Biology* 2016;198:40-46.
61. Saccone G, Berghella V. Omega-3 supplementation to prevent recurrent preterm birth: a systematic review and metaanalysis of randomized controlled trials. *American Journal of Obstetrics & Gynecology* 2015;213(2):135-40.
62. Salvig JD, Lamont RF. Evidence regarding an effect of marine n-3 fatty acids on preterm birth: a systematic review and meta-analysis. *Acta Obstetricia et Gynecologica Scandinavica* 2011;90(8):825-38.

For peer review only

63. Saccone G, Saccone I, Berghella V. Omega-3 long-chain polyunsaturated fatty acids and fish oil supplementation during pregnancy: which evidence? *Journal of Maternal-Fetal & Neonatal Medicine* 2016;29(15):2389-97.
64. Chaffee BW, King JC. Effect of zinc supplementation on pregnancy and infant outcomes: a systematic review. *Paediatric and Perinatal Epidemiology* 2012;26 Suppl 1:118-37.
65. Mori R, Ota E, Middleton P, et al. Zinc supplementation for improving pregnancy and infant outcome. *Cochrane Database of Systematic Reviews* 2012(7):CD000230.
66. Hofmeyr GJ, Lawrie TA, Atallah ÁN, et al. Calcium supplementation during pregnancy for preventing hypertensive disorders and related problems. *Cochrane database of systematic reviews* 2018(10)
67. Hofmeyr GJ, Manyame S, Medley N, et al. Calcium supplementation commencing before or early in pregnancy, for preventing hypertensive disorders of pregnancy. *Cochrane Database of Systematic Reviews* 2019(9)
68. Harding KB, Pena-Rosas JP, Webster AC, et al. Iodine supplementation for women during the preconception, pregnancy and postpartum period. *Cochrane Database of Systematic Reviews* 2017;3:CD011761.
69. Fall CH, Fisher DJ, Osmond C, et al. Multiple micronutrient supplementation during pregnancy in low-income countries: a meta-analysis of effects on birth size and length of gestation. *Food & Nutrition Bulletin* 2009;30(4 Suppl):S533-46.
70. Keats EC, Haider BA, Tam E, et al. Multiple-micronutrient supplementation for women during pregnancy. *Cochrane Database of Systematic Reviews* 2019(3)
71. Smith ER, Shankar AH, Wu LS, et al. Modifiers of the effect of maternal multiple micronutrient supplementation on stillbirth, birth outcomes, and infant mortality: a meta-analysis of individual patient data from 17 randomised trials in low-income and middle-income countries. *The Lancet Global Health* 2017;5(11):e1090-e100.
72. Corbella S, Del Fabbro M, Taschieri S, et al. Periodontal disease and adverse pregnancy outcomes: A systematic review. *Italian Oral Surgery* 2012;11(4):132-46.
73. Fogacci MF, Vettore MV, Leao AT. The effect of periodontal therapy on preterm low birth weight: a meta-analysis. *Obstetrics & Gynecology* 2011;117(1):153-65.
74. George A, Shamim S, Johnson M, et al. Periodontal treatment during pregnancy and birth outcomes: a meta-analysis of randomised trials. *International Journal of Evidence-Based Healthcare* 2011;9(2):122-47.
75. Pimentel Lopes De Oliveira GJ, Amaral Fontanari L, Chaves De Souza JA, et al. Effect of periodontal treatment on the incidence of preterm delivery: a systematic review. *Minerva Stomatologica* 2010;59(10):543-50.
76. Polyzos N, Polyzos I, Zavos A, et al. Obstetric outcomes after treatment of periodontal disease during pregnancy: systematic review and meta-analysis (Structured abstract). *Bmj* 2010;341(2):c7017.
77. Rosa MI, Pires PD, Medeiros LR, et al. Periodontal disease treatment and risk of preterm birth: a systematic review and meta-analysis. *Cadernos de Saude Publica* 2012;28(10):1823-33.
78. Shah M, Muley A, Muley P. Effect of nonsurgical periodontal therapy during gestation period on adverse pregnancy outcome: a systematic review. *Journal of Maternal-Fetal & Neonatal Medicine* 2013;26(17):1691-95.
79. Uppal A, Uppal S, Pinto A, et al. The effectiveness of periodontal disease treatment during pregnancy in reducing the risk of experiencing preterm birth and low birth

For peer review only

weight: a meta-analysis. *Journal of the American Dental Association* 2010;141(12):1423-34.
80. Kim AJ, Lo AJ, Pullin DA, et al. Scaling and root planing treatment for periodontitis to reduce preterm birth and low birth weight: a systematic review and meta-analysis of randomized controlled trials. *Journal of Periodontology* 2012;83(12):1508-19.
81. da Silva HEC, Stefani CM, de Santos Melo N, et al. Effect of intra-pregnancy nonsurgical periodontal therapy on inflammatory biomarkers and adverse pregnancy outcomes: a systematic review with meta-analysis. *Systematic Reviews* 2017;6(1):197.
82. Iheozor-Ejiofor Z, Middleton P, Esposito M, et al. Treating periodontal disease for preventing adverse birth outcomes in pregnant women. *Cochrane Database of Systematic Reviews* 2017;6:CD005297.
83. Schwendicke F, Karimbux N, Allareddy V, et al. Periodontal treatment for preventing adverse pregnancy outcomes: a meta- and trial sequential analysis. *PLoS ONE [Electronic Resource]* 2015;10(6):e0129060.
84. Guinto VT, De GB, Festin MR, et al. Different antibiotic regimens for treating asymptomatic bacteriuria in pregnancy. *Cochrane Database of Systematic Reviews* 2010(9) doi: 10.1002/14651858.CD007855.pub2
85. Smaill FM, Vazquez JC. Antibiotics for asymptomatic bacteriuria in pregnancy. *Cochrane Database of Systematic Reviews* 2015(8):CD000490.
86. Widmer M, Lopez I, Gülmezoglu AM, et al. Duration of treatment for asymptomatic bacteriuria during pregnancy. *Cochrane Database of Systematic Reviews* 2015(11)
87. Angelescu K, Nussbaumer-Streit B, Sieben W, et al. Benefits and harms of screening for and treatment of asymptomatic bacteriuria in pregnancy: a systematic review. *BMC Pregnancy & Childbirth* 2016;16(1):336.
88. Blencowe H, Cousens S, Kamb M, et al. Lives Saved Tool supplement detection and treatment of syphilis in pregnancy to reduce syphilis related stillbirths and neonatal mortality. *BMC Public Health* 2011;11 Suppl 3:S9.
89. Fell DB, Platt RW, Lanes A, et al. Fetal death and preterm birth associated with maternal influenza vaccination: systematic review. *BJOG: An International Journal of Obstetrics & Gynaecology* 2015;122(1):17-26.
90. Zhang C, Wang X, Liu D, et al. A systematic review and meta-analysis of fetal outcomes following the administration of influenza A/H1N1 vaccination during pregnancy. *International Journal of Gynaecology & Obstetrics* 2018;141(2):141-50.
91. Sangkomkamhang US, Lumbiganon P, Prasertcharoensuk W, et al. Antenatal lower genital tract infection screening and treatment programs for preventing preterm delivery. *Cochrane Database of Systematic Reviews* 2015(2) doi: 10.1002/14651858.CD006178.pub3
92. Schneeberger C, Geerlings SE, Middleton P, et al. Interventions for preventing recurrent urinary tract infection during pregnancy. *Cochrane Database of Systematic Reviews* 2015(7) doi: 10.1002/14651858.CD009279.pub3
93. Vazquez JC, Abalos E. Treatments for symptomatic urinary tract infections during pregnancy. *Cochrane Database of Systematic Reviews* 2011(1):CD002256.
94. Roberts CL, Algert CS, Rickard KL, et al. Treatment of vaginal candidiasis for the prevention of preterm birth: a systematic review and meta-analysis. *Systematic Reviews* 2015;4:31.

For peer review only

95. Flenady V, Hawley G, Stock OM, et al. Prophylactic antibiotics for inhibiting preterm labour with intact membranes. *Cochrane Database of Systematic Reviews* 2013(12):CD000246.
96. Thinkhamrop J, Hofmeyr GJ, Adetoro O, et al. Antibiotic prophylaxis during the second and third trimester to reduce adverse pregnancy outcomes and morbidity. *Cochrane Database of Systematic Reviews* 2015(6):CD002250.
97. "C. K. Manyando K, Dalessandro U, Okafor HU, et al. A systematic review of the safety and efficacy of artemether-lumefantrine against uncomplicated Plasmodium falciparum malaria during pregnancy. *Malaria Journal* 2012;11 (no pagination)(141)
98. Radeva-Petrova D, Kayentao K, ter Kuile FO, et al. Drugs for preventing malaria in pregnant women in endemic areas: any drug regimen versus placebo or no treatment. *Cochrane Database of Systematic Reviews* 2014(10):CD000169.
99. Gamble C, Ekwaru PJ, Garner P, et al. Insecticide-treated nets for the prevention of malaria in pregnancy: a systematic review of randomised controlled trials. *PLoS Med* 2007;4(3):e107.
100. Alfirevic Z, Stampalija T, Medley N. Cervical stitch (cerclage) for preventing preterm birth in singleton pregnancy. *Cochrane Database of Systematic Reviews* 2017;6:CD008991.
101. Berghella V, Ciardulli A, Rust OA, et al. Cerclage for sonographic short cervix in singleton gestations without prior spontaneous preterm birth: systematic review and meta-analysis of randomized controlled trials using individual patient-level data. *Ultrasound in Obstetrics & Gynecology* 2017;50(5):569-77.
102. Berghella V, Keeler SM, To MS, et al. Effectiveness of cerclage according to severity of cervical length shortening: a meta-analysis. *Ultrasound in Obstetrics & Gynecology* 2010;35(4):468-73.
103. Berghella V, Rafael TJ, Szychowski JM, et al. Cerclage for short cervix on ultrasonography in women with singleton gestations and previous preterm birth: a meta-analysis. *Obstetrics & Gynecology* 2011;117(3):663-71.
104. DeFranco E, Valent A, Newman T, et al. Adjunctive therapies to cerclage for the prevention of preterm birth: a systematic review (Provisional abstract). *Obstetrics and Gynecology International* 2013;2013(2):528158.
105. Ehsanipoor RM, Seligman NS, Saccone G, et al. Physical Examination-Indicated Cerclage: A Systematic Review and Meta-analysis. *Obstetrics & Gynecology* 2015;126(1):125-35.
106. Moawad GN, Tyan P, Bracke T, et al. Systematic Review of Transabdominal Cerclage Placed via Laparoscopy for the Prevention of Preterm Birth. *Journal of Minimally Invasive Gynecology* 2018;25(2):277-86.
107. Namouz S, Porat S, Okun N, et al. Emergency cerclage: literature review (Provisional abstract). *Obstetrical and Gynecological Survey* 2013;68(5):379-88.
108. Pergialiotis V, Vlachos DG, Prodromidou A, et al. Double versus single cervical cerclage for the prevention of preterm births. *Journal of Maternal-Fetal & Neonatal Medicine* 2015;28(4):379-85.
109. Rafael TJ, Berghella V, Alfirevic Z. Cervical stitch (cerclage) for preventing preterm birth in multiple pregnancy. *Cochrane Database of Systematic Reviews* 2014(9):CD009166.
110. Saccone G, Rust O, Althuisius S, et al. Cerclage for short cervix in twin pregnancies: systematic review and meta-analysis of randomized trials using individual patient-level data. *Acta Obstetricia et Gynecologica Scandinavica* 2015;94(4):352-58.

For peer review only

111. Smith J, DeFranco EA. Tocolytics used as adjunctive therapy at the time of cerclage placement: a systematic review. *Journal of Perinatology* 2015;35(8):561-65.
112. Zeybek B, Hill A, Menderes G, et al. Robot-Assisted Abdominal Cerclage During Pregnancy. *Journal of the Society of Laparoendoscopic Surgeons* 2016;20(4):Oct-Dec.
113. Liu X, Luo X, Xiao X, et al. Cervical cerclage for preventing preterm birth in twin pregnancies. A systematic review and meta-analysis (Provisional abstract). *Database of Abstracts of Reviews of Effects* 2013(4):632-38.
114. Conde-Agudelo A, Romero R, Da Fonseca E, et al. Vaginal progesterone is as effective as cervical cerclage to prevent preterm birth in women with a singleton gestation, previous spontaneous preterm birth, and a short cervix: updated indirect comparison meta-analysis. *American Journal of Obstetrics & Gynecology* 2018;219(1):10-25.
115. Conde-Agudelo A, Romero R, Nicolaides K, et al. Vaginal progesterone vs. cervical cerclage for the prevention of preterm birth in women with a sonographic short cervix, previous preterm birth, and singleton gestation: a systematic review and indirect comparison metaanalysis. *American Journal of Obstetrics & Gynecology* 2013;208(1):42.e41-42.e18.
116. Jarde A, Lutsiv O, Park CK, et al. Preterm birth prevention in twin pregnancies with progesterone, pessary, or cerclage: a systematic review and meta-analysis. *BJOG: An International Journal of Obstetrics & Gynaecology* 2017;124(8):1163-73.
117. Jarde A, Lutsiv O, Park CK, et al. Effectiveness of progesterone, cerclage and pessary for preventing preterm birth in singleton pregnancies: a systematic review and network meta-analysis. *BJOG: An International Journal of Obstetrics & Gynaecology* 2017;124(8):1176-89.
118. Crowther CA, Han S. Hospitalisation and bed rest for multiple pregnancy. *Cochrane Database of Systematic Reviews* 2010(7):CD000110.
119. Maloni J. Antepartum bed rest for pregnancy complications: efficacy and safety for preventing preterm birth (Provisional abstract). *Biological Research for Nursing* 2010;12(2):106-24.
120. Sosa CG, Althabe F, Belizan JM, et al. Bed rest in singleton pregnancies for preventing preterm birth. *Cochrane Database of Systematic Reviews* 2015(3):CD003581.
121. Jin Z, Chen L, Qiao D, et al. Cervical pessary for preventing preterm birth: a meta-analysis. *Journal of Maternal-Fetal and Neonatal Medicine* 2017:1-7.
122. Liem SMS, van Pampus MG, Mol BWJ, et al. Cervical Pessaries for the Prevention of Preterm Birth: A Systematic Review. *Obstetrics and Gynecology International* 2013;2013:576723. doi: 10.1155/2013/576723
123. Saccone G, Ciardulli A, Xodo S, et al. Cervical pessary for preventing preterm birth in twin pregnancies with short cervical length: a systematic review and meta-analysis. *Journal of Maternal-Fetal & Neonatal Medicine* 2017;30(24):2918-25.
124. Saccone G, Ciardulli A, Xodo S, et al. Cervical Pessary for Preventing Preterm Birth in Singleton Pregnancies with Short Cervical Length: A Systematic Review and Meta-analysis. *Obstetrical and Gynecological Survey* 2018;73(1):13-14.
125. Thangatorai R, Lim FC, Nalliah S. Cervical pessary in the prevention of preterm births in multiple pregnancies with a short cervix: PRISMA compliant systematic review and meta-analysis. *Journal of Maternal-Fetal & Neonatal Medicine* 2018;31(12):1638-45.

For peer review only

126. Zheng L, Dong J, Dai Y, et al. Cervical pessaries for the prevention of preterm birth: a systematic review and meta-analysis. *Journal of Maternal-Fetal & Neonatal Medicine* 2017:1-10.
127. Romero R, Conde-Agudelo A, Da Fonseca E, et al. Vaginal progesterone for preventing preterm birth and adverse perinatal outcomes in singleton gestations with a short cervix: a meta-analysis of individual patient data. *American Journal of Obstetrics & Gynecology* 2018;218(2):161-80.
128. Romero R, Nicolaides KH, Conde-Agudelo A, et al. Vaginal progesterone decreases preterm birth<=34weeks of gestation in women with a singleton pregnancy and a short cervix: an updated meta-analysis including data from the OPPTIMUM study. *Ultrasound in Obstetrics & Gynecology* 2016;48(3):308-17.
129. Saccone G, Khalifeh A, Elimian A, et al. Vaginal progesterone vs intramuscular 17alpha-hydroxyprogesterone caproate for prevention of recurrent spontaneous preterm birth in singleton gestations: systematic review and meta-analysis of randomized controlled trials. *Ultrasound in Obstetrics & Gynecology* 2017;49(3):315-21.
130. Su LL, Samuel M, Chong YS. Progestational agents for treating threatened or established preterm labour. *Cochrane Database of Systematic Reviews* 2010(1):CD006770.
131. Suhag A, Saccone G, Berghella V. Vaginal progesterone for maintenance tocolysis: a systematic review and metaanalysis of randomized trials. *American Journal of Obstetrics & Gynecology* 2015;213(4):479-87.
132. Dodd JM, Grivell RM, Obrien CM, et al. Prenatal administration of progestogens for preventing spontaneous preterm birth in women with a multiple pregnancy. *Cochrane Database of Systematic Reviews* 2019(11) doi: 10.1002/14651858.CD012024.pub3
133. Romero R, Conde-Agudelo A, El-Refaie W, et al. Vaginal progesterone decreases preterm birth and neonatal morbidity and mortality in women with a twin gestation and a short cervix: an updated meta-analysis of individual patient data. *Ultrasound in Obstetrics & Gynecology* 2017;49(3):303-14.
134. Sotiriadis A, Papatheodorou S, Makrydimas G. Perinatal outcome in women treated with progesterone for the prevention of preterm birth: a meta-analysis. *Ultrasound in Obstetrics & Gynecology* 2012;40(3):257-66.
135. Lim CE, Ho KK, Cheng NC, et al. Combined oestrogen and progesterone for preventing miscarriage. *Cochrane Database of Systematic Reviews* 2013(9):CD009278.
136. Norman JE, Mackenzie F, Owen P, et al. Progesterone for the prevention of preterm birth in twin pregnancy (STOPPIT): A randomized, double-blind, placebo-controlled study and meta-analysis. *Obstetrical and Gynecological Survey* 2009;64(10):646-48.
137. Likis FE, Edwards DR, Andrews JC, et al. Progestogens for preterm birth prevention: a systematic review and meta-analysis. *Obstetrics & Gynecology* 2012;120(4):897-907.
138. Palacio M, Ronzoni S, Sanchez-Ramos L, et al. Progestogens as Maintenance Treatment in Arrested Preterm Labor: A Systematic Review and Meta-analysis. *Obstetrics & Gynecology* 2016;128(5):989-1000.
139. Prior M, Hibberd R, Asemota N, et al. Inadvertent P-hacking among trials and systematic reviews of the effect of progestogens in pregnancy? A systematic review and meta-analysis. *BJOG: An International Journal of Obstetrics and Gynaecology* 2017;124(7):1008-15.

For peer review only

140. Rode L, Langhoff-Roos J, Andersson C, et al. Systematic review of progesterone for the prevention of preterm birth in singleton pregnancies. *Acta Obstetricia et Gynecologica Scandinavica* 2009;88(11):1180-89.
141. Schmouder VM, Prescott GM, Franco A, et al. The rebirth of progesterone in the prevention of preterm labor. *Annals of Pharmacotherapy* 2013;47(4):527-36.
142. Velez Edwards DR, Likis FE, Andrews JC, et al. Progestogens for preterm birth prevention: a systematic review and meta-analysis by drug route. *Archives of Gynecology & Obstetrics* 2013;287(6):1059-66.
143. Chawanpaiboon S, Laopaiboon M, Lumbiganon P, et al. Terbutaline pump maintenance therapy after threatened preterm labour for reducing adverse neonatal outcomes. *Cochrane Database of Systematic Reviews* 2014(3):CD010800.
144. Crowther CA, Brown J, McKinlay CJ, et al. Magnesium sulphate for preventing preterm birth in threatened preterm labour. *Cochrane Database of Systematic Reviews* 2014(8):CD001060.
145. Gaudet LM, Singh K, Weeks L, et al. Effectiveness of terbutaline pump for the prevention of preterm birth. A systematic review and meta-analysis. *PLoS ONE [Electronic Resource]* 2012;7(2):e31679.
146. Haas D, Caldwell D, Kirkpatrick P, et al. Tocolytic therapy for preterm delivery: systematic review and network meta-analysis (Structured abstract). *Bmj* 2012;344(2):e6226.
147. McNamara HC, Crowther CA, Brown J. Different treatment regimens of magnesium sulphate for tocolysis in women in preterm labour. *Cochrane Database of Systematic Reviews* 2015(12):CD011200.
148. Vogel JP, Nardin JM, Dowswell T, et al. Combination of tocolytic agents for inhibiting preterm labour. *Cochrane Database of Systematic Reviews* 2014(7):CD006169.
149. Dodd JM, Crowther CA, Middleton P. Oral betamimetics for maintenance therapy after threatened preterm labour. *Cochrane Database of Systematic Reviews* 2012;12:CD003927.
150. Flenady V, Reinebrant HE, Liley HG, et al. Oxytocin receptor antagonists for inhibiting preterm labour. *Cochrane Database of Systematic Reviews* 2014(6):CD004452.
151. Giorgino FL, Egan CG. Use of isoxsuprine hydrochloride as a tocolytic agent in the treatment of preterm labour: a systematic review of previous literature. *Arzneimittel-Forschung* 2010;60(7):415-20.
152. Mackeen AD, Seibel-Seamon J, Grimes-Dennis J, et al. Tocolytics for preterm premature rupture of membranes. *Cochrane Database of Systematic Reviews* 2011(10):CD007062.
153. Saccone G, Suhag A, Berghella V. 17-alpha-hydroxyprogesterone caproate for maintenance tocolysis: a systematic review and metaanalysis of randomized trials. *American Journal of Obstetrics & Gynecology* 2015;213(1):16-22.
154. van Vliet EOG, Dijkema GH, Schuit E, et al. Nifedipine maintenance tocolysis and perinatal outcome: an individual participant data meta-analysis. *BJOG: An International Journal of Obstetrics and Gynaecology* 2016;123(11):1753-60.
155. Yamasmit W, Chaithongwongwatthana S, Tolosa JE, et al. Prophylactic oral betamimetics for reducing preterm birth in women with a twin pregnancy. *Cochrane Database of Systematic Reviews* 2012(9):CD004733.
156. Ota E, Mori R, Middleton P, et al. Zinc supplementation for improving pregnancy and infant outcome. *Cochrane Database of Systematic Reviews* 2015(2)

For peer review only

157. Bellows BW, Conlon CM, Higgs ES, et al. A taxonomy and results from a comprehensive review of 28 maternal health voucher programmes. *Journal of health, population, and nutrition* 2013;31(4 Suppl 2):S106.
158. Atal I, Trinquart L, Ravaud P, et al. A mapping of 115,000 randomized trials revealed a mismatch between research effort and health needs in non–high-income regions. *Journal of clinical epidemiology* 2018;98:123-32.
159. Dab W. Commentary on SPHERE (Strengthening Public Health Research in Europe) literature reviews. *European journal of public health* 2007;17(suppl_1):8-9.
160. Shepherd V. Research involving adults lacking capacity to consent: the impact of research regulation on 'evidence biased'medicine. *BMC Medical Ethics* 2016;17(1):1-8.
161. Organization WH. Born too soon: the global action report on preterm birth. 2012
162. Della Rosa PA, Miglioli C, Caglioni M, et al. A hierarchical procedure to select intrauterine and extrauterine factors for methodological validation of preterm birth risk estimation. *BMC pregnancy and childbirth* 2021;21(1):1-17.
163. Linde K, Scholz M, Ramirez G, et al. Impact of study quality on outcome in placebo-controlled trials of homeopathy. *Journal of clinical epidemiology* 1999;52(7):631-36.
164. Schulz KF, Chalmers I, Hayes RJ, et al. Empirical evidence of bias: dimensions of methodological quality associated with estimates of treatment effects in controlled trials. *Jama* 1995;273(5):408-12.
165. Moher D, Liberati A, Tetzlaff J, et al. Preferred reporting items for systematic reviews and meta-analyses: the PRISMA statement. *PLoS medicine* 2009;6(7):e1000097.
166. Guise J-M, Butler ME, Chang C, et al. AHRQ series on complex intervention systematic reviews—paper 6: PRISMA-CI extension statement and checklist. *Journal of clinical epidemiology* 2017;90:43-50.
167. Banke-Thomas A, Wong KL, Ayomoh FI, et al. "In cities, it's not far, but it takes long": comparing estimated and replicated travel times to reach life-saving obstetric care in Lagos, Nigeria. *BMJ Global Health* 2021;6(1):e004318.
168. Rogers L, De Brún A, McAuliffe E. Defining and assessing context in healthcare implementation studies: a systematic review. *BMC Health Services Research* 2020;20(1):591. doi: 10.1186/s12913-020-05212-7
169. Vogel JP, Chawanpaiboon S, Moller A-B, et al. The global epidemiology of preterm birth. *Best Practice & Research Clinical Obstetrics & Gynaecology* 2018;52:3-12.
170. C S. UKRI Official Development Assistance letter 11 March 2021: UKRI; 2021 [Available from: https://www.ukri.org/our-work/ukri-oda-letter-11-march-2021/ accessed March 23 2021.
171. Organization WH. Millennium development goals. 2008

For peer review only

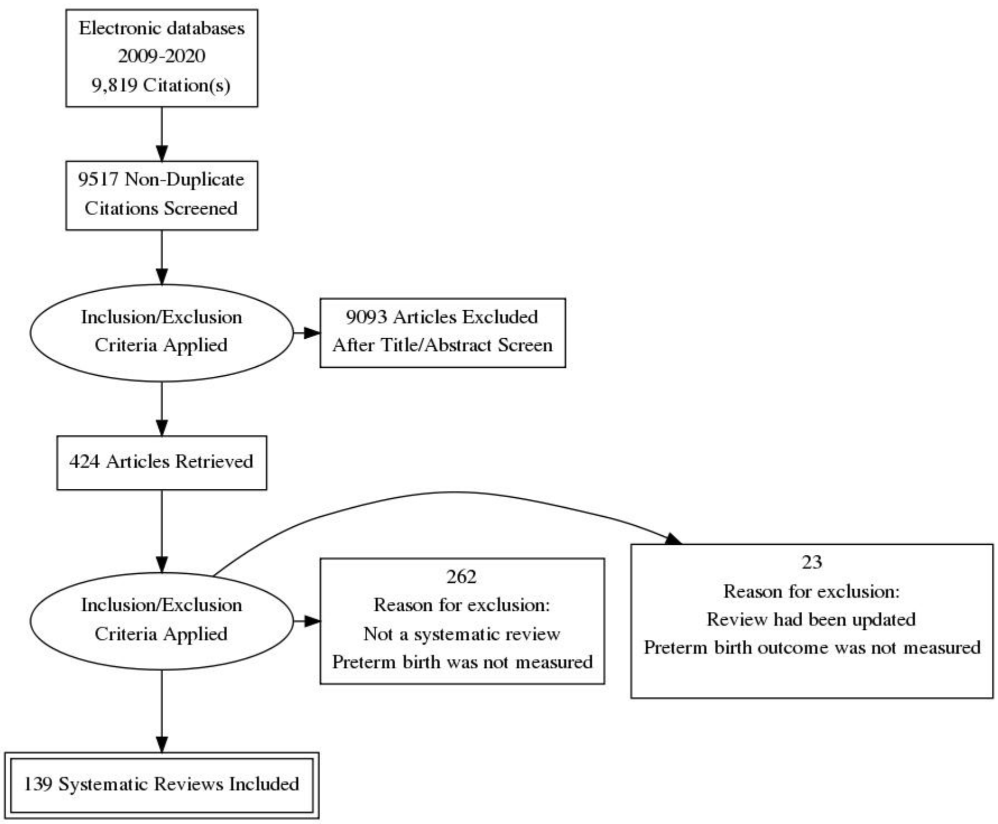

314x258mm (72 x 72 DPI)

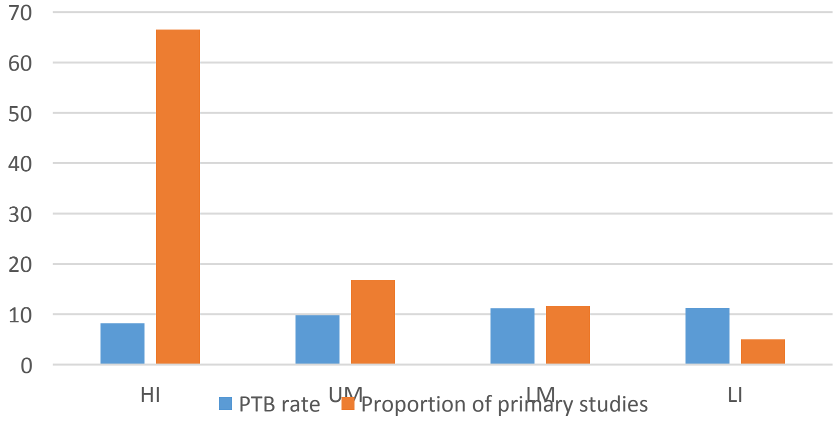

Figure 2: Rates of PTB and proportion of primary studies undertaken in each setting.

Figure 3: Systematic reviews of interventions showing reduction in risk of PTB

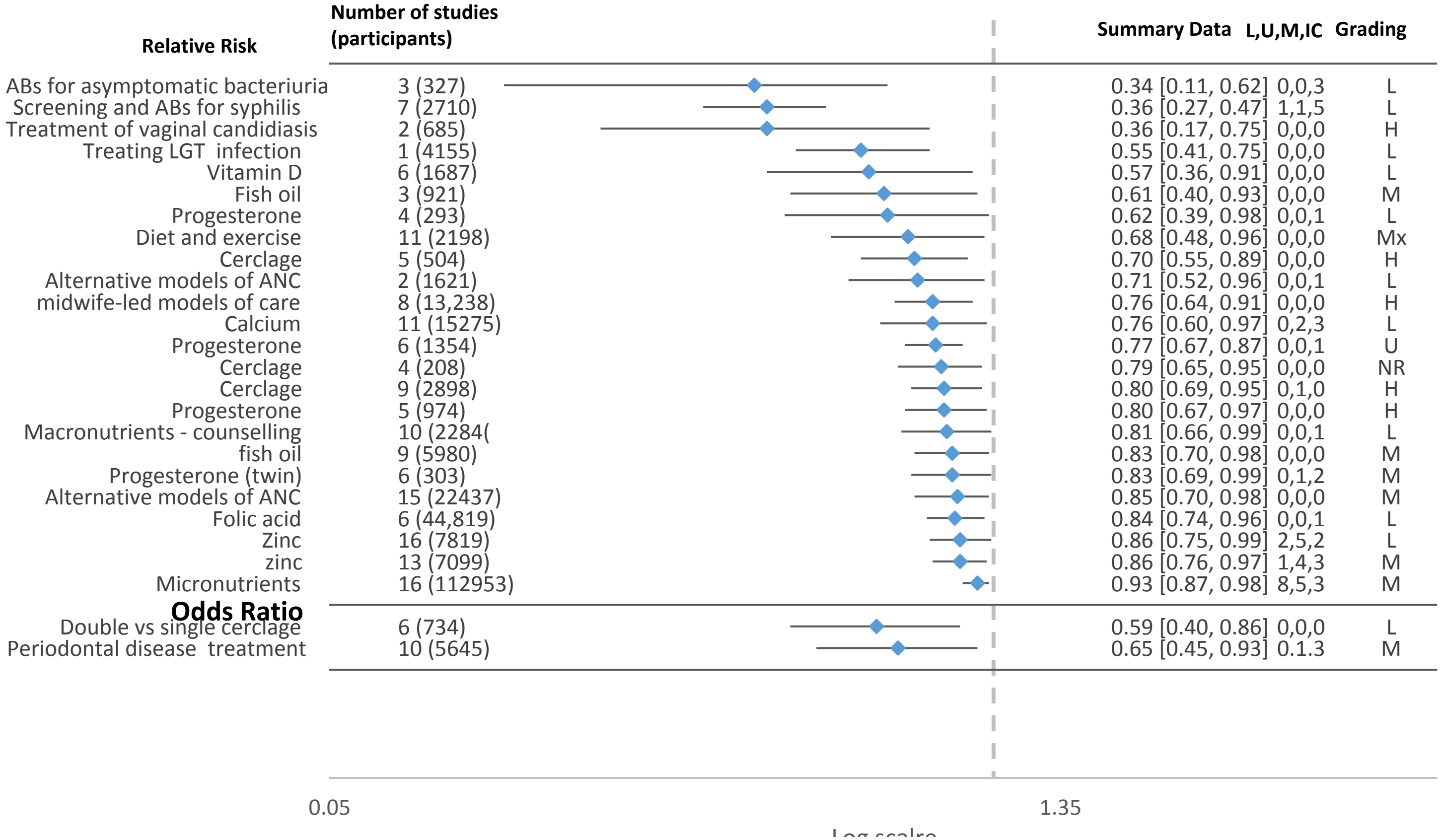

Appendix 1 Search Strategies

Search strategy from MEDLINE (via Ovid SP)

The McMaster Reviews Search Filter was utilised (Line 6), available from: https://hiru.mcmaster.ca/hiru/HIRU_Hedges_MEDLINE_Strategies.aspx#Reviews [Accessed 26 March 2021]. The Allen et al review referred to in Line 8 is: Felicity Allen, Ron Gray, Laura Oakley, Jennifer J Kurinczuk, Peter Brocklehurst, Jennifer Hollowell. Inequalities in Infant Mortality Project Evidence Map Report 2. Technical guide to the infant mortality evidence map: systematic reviews of interventions targeting infant mortality. Oxford: National Perinatal Epidemiology Unit, 2009. Available at: Infant-Mortality-Technical-Guide.pdf (ox.ac.uk) [Accessed 17 January 2022]

1 (pre-term birth* or pre term birth* or preterm birth*).tw.<br>2 exp OBSTETRIC LABOR, PREMATURE/<br>3 exp PREMATURE BIRTH/<br>4 ((preterm or pre-term or premature) adj3 (birth* or labo?r or deliver*)).tw.<br>5 or/1-4	**Pre-term birth terms**
6 meta analysis.mp,pt. or review.pt. or search:.tw.	**Systematic Review (SR) Filter**
7 5 and 6	**Pre-term birth terms and SR Filter**
8 (2009 04* or 2009 05* or 2009 06* or 2009 07* or 2009 08* or 2009 09* or 2009 10* or 2009 11* or 2009 12* or 2010* or 2011* or 2012* or 2013* or 2014* or 2015* or 2016* or 2017* or 2018* or 2019* or 2020*).dt.	**Added to MEDLINE post 1 April 2009 (when Allen et al searches were conducted)**
9 7 and 8	**Systematic Reviews post 1 April 2009**
10 limit 9 to english language	

For peer review only

11	limit 10 to humans	**Systematic Reviews post 1 April 2009 (English Language and Human only)**

The MEDLINE search strategy was then translated to the Cochrane Database of Systematic Reviews, PsycINFO (via Ovid), Embase (via Ovid), and CINAHL via EBSCO. Full search strategies for each database are presented below.

Cochrane Database of Systematic Reviews

#1 (pre-term birth* or pre term birth* or preterm birth*):ti,ab,kw
#2 MeSH descriptor: [Obstetric Labor, Premature] explode all trees
#3 MeSH descriptor: [Premature Birth] explode all trees
#4 ((preterm or pre-term or premature) near/3 (birth* or labo?r or deliver*))
#5 #1 or #2 or #3 or #4
with Publication Year from 2009 to 2020

PsycINFO (via Ovid)

1. (pre-term birth* or pre term birth* or preterm birth*).tw.
2. exp Premature Birth/
3. ((preterm or pre-term or premature) adj3 (birth* or labo?r or deliver*)).tw.
4. 1 or 2 or 3
5. (meta-analysis or search:).tw.
6. 4 and 5
7. (2009 04* or 2009 05* or 2009 06* or 2009 07* or 2009 08* or 2009 09* or 2009 10* or 2009 11* or 2009 12* or 2010* or 2011* or 2012* or 2013* or 2014* or 2015* or 2016* or 2017* or 2018* or 2019* or 2020*).dp.
8. 6 and 7
9. limit 8 to (human and english language)

Embase (via Ovid)

1. (pre-term birth* or pre term birth* or preterm birth*).tw.
2. exp premature labor/
3. exp prematurity/
4. ((preterm or pre-term or premature) adj3 (birth* or labo?r or deliver*)).tw.
5. 1 or 2 or 3 or 4
6. (meta-analysis or systematic review).tw.
7. 5 and 6
8. (2009 04* or 2009 05* or 2009 06* or 2009 07* or 2009 08* or 2009 09* or 2009 10* or 2009 11* or 2009 12* or 2010* or 2011* or 2012* or 2013* or 2014* or 2015* or 2016* or 2017* or 2018* or 2019* or 2020*).dc.
9. 7 and 8

For peer review only

10. limit 9 to (human and english language)
11. limit 10 to embase

CINAHL via EBSCO

S1 pre-term birth* or pre term birth* or preterm birth*
S2 (MH "Labor, Premature")
S3 (MH "Childbirth, Premature")
S4 ((preterm or pre-term or premature) N3 (birth* or labo?r or deliver*))
S5 S1 OR S2 OR S3 OR S4
S6 meta-analysis or systematic review
S7 S5 AND S6 Limiters - Published Date: 20090101-20201231

For peer review only

Preferred Reporting Items for Systematic reviews and Meta-Analyses extension for Scoping Reviews (PRISMA-ScR) Checklist

SECTION	ITEM	PRISMA-ScR CHECKLIST ITEM	REPORTED ON PAGE #
**TITLE**			
Title	1	Identify the report as a scoping review.	Yes
**ABSTRACT**			
Structured summary	2	Provide a structured summary that includes (as applicable): background, objectives, eligibility criteria, sources of evidence, charting methods, results, and conclusions that relate to the review questions and objectives.	Yes
**INTRODUCTION**			
Rationale	3	Describe the rationale for the review in the context of what is already known. Explain why the review questions/objectives lend themselves to a scoping review approach.	Yes
Objectives	4	Provide an explicit statement of the questions and objectives being addressed with reference to their key elements (e.g., population or participants, concepts, and context) or other relevant key elements used to conceptualize the review questions and/or objectives.	Yes
**METHODS**			
Protocol and registration	5	Indicate whether a review protocol exists; state if and where it can be accessed (e.g., a Web address); and if available, provide registration information, including the registration number.	unpublished protocol which is available on request to the
Eligibility criteria	6	Specify characteristics of the sources of evidence used as eligibility criteria (e.g., years considered, language, and publication status), and provide a rationale.	Yes
Information sources*	7	Describe all information sources in the search (e.g., databases with dates of coverage and contact with authors to identify additional sources), as well as the date the most recent search was executed.	Yes
Search	8	Present the full electronic search strategy for at least 1 database, including any limits used, such that it could be repeated.	Yes
Selection of sources of evidence†	9	State the process for selecting sources of evidence (i.e., screening and eligibility) included in the scoping review.	Yes
Data charting process‡	10	Describe the methods of charting data from the included sources of evidence (e.g., calibrated forms or forms that have been tested by the team before their use, and whether data charting was done independently or in duplicate) and any processes for obtaining and confirming data from investigators.	Yes
Data items	11	List and define all variables for which data were sought and any assumptions and simplifications made.	Yes
Critical appraisal of individual sources of evidence§	12	If done, provide a rationale for conducting a critical appraisal of included sources of evidence; describe the methods used and how this information was used in any data synthesis (if appropriate).	We did not undertake quality appraisal
Synthesis of results	13	Describe the methods of handling and summarizing the data that were charted.	Yes

St. Michael's
Inspired Care.
Inspiring Science.

For peer review only - http://bmjopen.bmj.com/site/about/guidelines.xhtml

SECTION	ITEM	PRISMA-ScR CHECKLIST ITEM	REPORTED ON PAGE #
**RESULTS**			
Selection of sources of evidence	14	Give numbers of sources of evidence screened, assessed for eligibility, and included in the review, with reasons for exclusions at each stage, ideally using a flow diagram.	Yes
Characteristics of sources of evidence	15	For each source of evidence, present characteristics for which data were charted and provide the citations.	Yes
Critical appraisal within sources of evidence	16	If done, present data on critical appraisal of included sources of evidence (see item 12).	Critical appraisal was not undertaken
Results of individual sources of evidence	17	For each included source of evidence, present the relevant data that were charted that relate to the review questions and objectives.	Yes
Synthesis of results	18	Summarize and/or present the charting results as they relate to the review questions and objectives.	Yes
**DISCUSSION**			
Summary of evidence	19	Summarize the main results (including an overview of concepts, themes, and types of evidence available), link to the review questions and objectives, and consider the relevance to key groups.	Yes
Limitations	20	Discuss the limitations of the scoping review process.	Yes
Conclusions	21	Provide a general interpretation of the results with respect to the review questions and objectives, as well as potential implications and/or next steps.	Yes
**FUNDING**			
Funding	22	Describe sources of funding for the included sources of evidence, as well as sources of funding for the scoping review. Describe the role of the funders of the scoping review.	Yes

JBI = Joanna Briggs Institute; PRISMA-ScR = Preferred Reporting Items for Systematic reviews and Meta-Analyses extension for Scoping Reviews.

* Where *sources of evidence* (see second footnote) are compiled from, such as bibliographic databases, social media platforms, and Web sites.

† A more inclusive/heterogeneous term used to account for the different types of evidence or data sources (e.g., quantitative and/or qualitative research, expert opinion, and policy documents) that may be eligible in a scoping review as opposed to only studies. This is not to be confused with *information sources* (see first footnote).

‡ The frameworks by Arksey and O'Malley (6) and Levac and colleagues (7) and the JBI guidance (4, 5) refer to the process of data extraction in a scoping review as data charting.

§ The process of systematically examining research evidence to assess its validity, results, and relevance before using it to inform a decision. This term is used for items 12 and 19 instead of "risk of bias" (which is more applicable to systematic reviews of interventions) to include and acknowledge the various sources of evidence that may be used in a scoping review (e.g., quantitative and/or qualitative research, expert opinion, and policy document).

From: Tricco AC, Lillie E, Zarin W, O'Brien KK, Colquhoun H, Levac D, et al. PRISMA Extension for Scoping Reviews (PRISMAScR): Checklist and Explanation. Ann Intern Med. 2018;169:467–473. doi: 10.7326/M18-0850.

BMJ Open

Interventions for the prevention of spontaneous preterm birth: a scoping review of systematic reviews

Journal:	*BMJ Open*
Manuscript ID	bmjopen-2021-052576.R2
Article Type:	Original research
Date Submitted by the Author:	31-Mar-2022
Complete List of Authors:	Campbell, Fiona; The University of Sheffield, ScHARR<br>Salam, Shumona; The University of Sheffield, Department of Oncology and Metabolism<br>Sutton , Anthea; The University of Sheffield, ScHARR<br>Jayasooriya, Shamanthi; The University of Sheffield, Academic Unit of Primary Medical Care<br>Mitchell, Caroline; The University of Sheffield, Academic Unit of Primary Medical Care<br>Amabebe, Emmanuel; The University of Sheffield, Department of Oncology and Metabolism<br>Balen, Julie; The University of Sheffield,<br>Gillespie, Bronwen; The University of Sheffield, Department of Oncology and Metabolism<br>Parris, Kerry ; The University of Sheffield, Department of Oncology and Metabolism<br>Soma-Pillay, Priya; University of Pretoria, Steve Biko Academic Hospital<br>Chauke, Lawrence; University of Witwatersrand Department of Obstetrics and Gynaecology<br>Narice, Brenda; The University of Sheffield, Department of Oncology and Metabolism<br>Anumba, Dilichukwu; The University of Sheffield, Department of Oncology and Metabolism
**Primary Subject Heading**:	Evidence based practice
Secondary Subject Heading:	Public health, Reproductive medicine, Global health
Keywords:	Maternal medicine < OBSTETRICS, International health services < HEALTH SERVICES ADMINISTRATION & MANAGEMENT, Public health < INFECTIOUS DISEASES, Health policy < HEALTH SERVICES ADMINISTRATION & MANAGEMENT

SCHOLARONE™
Manuscripts

I, the Submitting Author has the right to grant and does grant on behalf of all authors of the Work (as defined in the below author licence), an exclusive licence and/or a non-exclusive licence for contributions from authors who are: i) UK Crown employees; ii) where BMJ has agreed a CC-BY licence shall apply, and/or iii) in accordance with the terms applicable for US Federal Government officers or employees acting as part of their official duties; on a worldwide, perpetual, irrevocable, royalty-free basis to BMJ Publishing Group Ltd ("BMJ") its licensees and where the relevant Journal is co-owned by BMJ to the co-owners of the Journal, to publish the Work in this journal and any other BMJ products and to exploit all rights, as set out in our licence.

The Submitting Author accepts and understands that any supply made under these terms is made by BMJ to the Submitting Author unless you are acting as an employee on behalf of your employer or a postgraduate student of an affiliated institution which is paying any applicable article publishing charge ("APC") for Open Access articles. Where the Submitting Author wishes to make the Work available on an Open Access basis (and intends to pay the relevant APC), the terms of reuse of such Open Access shall be governed by a Creative Commons licence – details of these licences and which Creative Commons licence will apply to this Work are set out in our licence referred to above.

Other than as permitted in any relevant BMJ Author's Self Archiving Policies, I confirm this Work has not been accepted for publication elsewhere, is not being considered for publication elsewhere and does not duplicate material already published. I confirm all authors consent to publication of this Work and authorise the granting of this licence.

For peer review only

Interventions for the prevention of spontaneous preterm birth: a scoping review of systematic reviews

Fiona Campbell[1] Shumona Salam[3], Anthea Sutton[1], Shamanthi Maya Jayasooriya[2], Caroline Mitchell[2] Emmanuel Aamabebe[3] Julie Balen[1] Bronwen Gillespie[3] Kerry Parris[3] Priya Soma-Pillay[5], Lawrence Chauke[4] , Brenda Narice[3] , Dilichukwu O Anumba[3],

[1]School of Health and Related Research, University of Sheffield, Sheffield, UK
[2]Academic Unit of Primary Medical Care, University of Sheffield, Sheffield, UK
[3]Department of Oncology and Metabolism, University of Sheffield
[4]Witwatersrand, Johannesburg, South Africa.
[5]Steve Biko Academic Hospital and University of Pretoria, Pretoria, South Africa

Corresponding Author:
Fiona Campbell
ScHARR, University of Sheffield, Regent Court, 30 Regent Street, S1 4DA,
f.campbell@sheffield.ac.uk
https://orcid.org/0000-0002-4141-8863

Data extracted from included studies is available on request by email from the corresponding author: f.campbell@sheffield.ac.uk

Work Count: 3,473

Keywords: Preterm Birth, Scoping Review, Health services research, Meta-analyses

Contributors: PRIME (Preterm birth Prevention and Management) research program established the study objectives, FC, PSP, LC prepared the protocol, AS designed search strategies, SS, SJ, CM EA, JB, BG, KP, BN collected and analysed or interpreted data. FC prepared manuscript. SS, AS, SJ, CM, EA, JB, BG, KP, PSP, LC, BN, DA edited or read and approved the final manuscript.

Funding: NIHR provided funding for researcher support.

Competing Interests: We declare that there are no competing interests.

Data availability statement: Data extracted from included studies is available on request by email from the corresponding author

Ethical approval: Ethical approval was not required for this study as it drew on existing published research and did not involve human or animal participants.

For peer review only

Abstract

Background: Globally, 11% of babies are born preterm each year. Preterm birth (PTB) a leading cause of neonatal death and under-5 mortality and morbidity, with lifelong sequelae in those who survive. PTB disproportionately impacts low- and middle income countries **(**LMICs) where the burden is highest.

Objectives: This scoping review sought to the evidence for interventions that reduce the risk of PTB, focusing on the evidence from LMICs and describing how context is considered in evidence synthesis.

Design: We conducted a scoping review, to describe this wide topic area. We searched five electronic databases (2009-2020) and contacted experts to identify relevant systematic reviews of interventions to reduce the risk of PTB. We included published systematic reviews that examined the effectiveness of interventions and their effect on reducing the risk of PTB. Data was extracted and is described narratively.

Results: 139 published systematic reviews were included in the review. Interventions were categorised as primary or secondary. The interventions where the results showed a greater effect size and consistency across review findings included treatment of syphilis and vaginal candidiasis, vitamin D supplementation and cervical cerclage. Included in the 139 reviews were 1372 unique primary source studies. 28% primary studies were undertaken in LMIC contexts and only 4.5% undertaken in a low income country (LIC) Only 10.8% of the reviews sought to explore the impact of context on findings, and 19.4% reviews did not report the settings or the primary studies

Conclusion: This scoping review highlights the lack of research evidence derived from contexts where the burden of PTB globally is greatest. The lack of rigour in addressing contextual applicability within systematic review methods is also highlighted. This presents a risk of inappropriate and unsafe recommendations for practice within these contexts. It also highlights a need for primary research, developing and testing interventions LIC settings.

Strengths and Limitations of this Study

- Scoping review methodology enabled us to look at a broad topic area and analyse how context is taken into account in the included systematic reviews. Primary studies not reported in systematic reviews will therefore have not been included in our analysis.
- We were not able to identify the setting of all primary studies where this was not reported and there is a risk that some studies, which have multiple publications may have been double counted.
- We only included systematic reviews published in English.

BACKGROUND

For peer review only

Preterm birth (PTB) is a global and public health priority. It is defined by the World Health Organization (WHO) as delivery before 37 completed weeks of gestation, with extremely preterm delivery defined as occurring at less than 28 weeks, very preterm delivery occurring between 28 and 32 weeks, and moderate to late preterm delivery occurring from 32 through 36 weeks.[1] It is one of the leading causes of neonatal death and under five mortality and morbidity, with lifelong sequelae.[2] Children born prematurely have increased risks of cognitive problems, such as academic underachievement, behavioural problems and cerebral palsy than those born at full term.[3] They are more likely to experience hospital admission due to infection, particularly during infancy.[4] For parents, the financial, social and emotional effects are devastating.[3]

The global burden of PTB is falling more heavily on countries with fewer resources to manage the medical, social, and economic complexities of caring for premature infants. Globally, there are approximately 15 million live preterm births each year, which is estimated to be about 11% of all deliveries each year, ranging from about 8.7% in northern Europe to 13.4% in North Africa.[5 6] The majority of PTBs occur in Low- or Middle Income countries (LMICs) .[6] The highest PTB rates in 2014 occurred in southeast Asia, south Asia and sub-Saharan Africa. Nine of the 11 countries with the highest rates were in Africa. Furthermore, 60% of all PTBs were estimated to have occurred in sub-Saharan Africa and south Asia accounting for just over nine million of the almost 15 million PTBs that occurred worldwide in 2010 resulting in a PTB rate of 12.8% in those settings.

Patterns of PTB differ between high-income countries and LMICs. However, the differences in these patterns, causes and distribution of PTB is unclear and have not been fully explored. PTB is multifactorial in its aetiology and has distinct biological pathways. The aetiologies differ according to gestational age, ethnicity and characteristics unique to each population. In order to redress the burden of PTB in LMICs, additional insight into the causative and associated factors in these settings is required.

While a number of reviews and overviews of reviews of interventions to reduce the risk of PTB have been undertaken[7-10], none have explored how many of the primary studies included in these reviews were undertaken in LMIC contexts. It is clear that some interventions that are effective in HIC (high income country) contexts but may be harmful in LMIC settings, such as the use of antenatal corticosteroids[11] and cerclage.[12] It is also possible that treatments effective in HIC contexts may be even more beneficial or appropriate in LMIC contexts, such as nutritional supplements, interventions to increase birth spacing, or interventions to improve the accuracy of measuring gestational age.

We have undertaken a broad scoping review of systematic reviews on interventions to reduce the risk of PTB identifying primary studies undertaken in LMICs. This will allow us to identify potential areas for further synthesis of the evidence and also to identify gaps in the research in order to direct future primary research.

For peer review only

Review objectives

1. To identify systematic reviews that have sought to explore the effectiveness, safety and acceptability of interventions to prevent PTB.

2. To map research evidence to global settings to identify the geographical and economic contexts in which evidence is derived.

3. To identify where gaps in the research base exist (for real world, effectiveness, pragmatic studies) in LMIC contexts to inform future research and to generate research priorities.

4. To describe the methods used in meta-analysis to take into account geographical and regional differences in PTB.

METHODS

We used a scoping review methodology[13] to describe the existing evidence (systematic reviews) available across primary and secondary interventions to prevent PTB,published between 2009 and 2020. Systematic scoping draws upon methods described by Arksey & O'Malley (2005)[14] for scoping reviews: "[…a form of knowledge synthesis that addresses an exploratory research question aimed at scoping key concepts, types of evidence, and gaps in research related to a defined area or field by systematically searching, selecting, and synthesizing existing knowledge".[14] The approach enabled us to highlight the evidence gap and to assist with simultaneously undertaking a research prioritisation exercise and guideline development, as well as to inform a broader programme of research that aimed to develop effective postnatal interventions to mitigate PTB in LMIC settings. It also enabled us to generate a mega-map, an interactive table supported on our project website and designed as a visual tool to identify research gaps and facilitate ready access to relevant evidence. https://www.primeglobalhealth.co.uk/evidence-map-2-7-2020.html.

Identifying relevant studies

Relevant systematic reviews were identified by systematic searches in the following electronic databases: Ovid MEDLINE, Cochrane Database of Systematic Reviews (CDSR), PsycINFO via Ovid, EMBASE via Ovid and CINAHL via EBSCO. Each database was searched using the database thesaurus and the key word/free text method with terms relating to preterm birth combined with a systematic reviews filter. The search strategy, incorporated the following limitations: articles written in English, and Human studies only from April 2009 to July 2020. Relevant systematic reviews were identified by systematic searches in the following electronic databases: MEDLINE, The Cochrane Library, PsycINFO, EMBASE and CINAHL. Each database was searched using the database thesaurus and the key word/free text method. The search strategy, incorporated the following limitations: articles written in English, and Human studies only from April 2009 to July 2020. The date limit was selected due to the existence of a previous review for which the studies were conducted in April 2009.[15] Full search strategies have been described and published.[16]

We began with a framework of interventions identified by two existing reviews[7,8] as these were broad in their focus and encompassed a range of interventions. Any new intervention types identified during the screening process were then added to the map.

For peer review only

The process of study selection was based on inclusion and exclusion criteria as described in Table 1. After removal of duplicates and irrelevant studies, based on the titles and abstracts, all potentially relevant reviews were read in full. Citations were screen by two reviewers (FC and one of the following team members SS, SJ, EA, JB, BG, BN, KP) independently and differences were resolved by discussion.

Table 1 Inclusion/ exclusion criteria based on PICOS

Population

- Pregnant women at less than 37 completed weeks gestation without signs of threatened preterm labour or premature rupture of membranes (PPROM).
- Excluded reviews where the study population was defined by co-morbidities.

Intervention

- All interventions deliverable during pregnancy to prevent spontaneous preterm birth, (these included clinical, behavioural and nutritional interventions and health systems and policy interventions).
- All interventions assessed the risk of preterm birth.
- Excluded interventions given to pregnant women to improve neonatal outcomes.

Comparators

- We included any comparator, including placebo or alternative treatments.

Outcomes

- We included reviews which focused to PTB as an outcome.
- Where it is reported, we state how many of the primary studies measured PTB as an outcome and the resulting data used in the synthesis.

Study design

1. Systematic reviews published between April 2009-July 2020, of studies that have evaluated interventions to prevent PTB, or that measured PTB as a relevant outcome.

Outcomes

1. Preterm birth (<28, <34, <37 weeks gestation) .
2. We recorded neonatal outcomes and adverse outcomes if reported within the review.

For peer review only

Data extraction and coding

Data were extracted using an agreed and piloted template and coded in Excel by two reviewers working independently (FC and one of the following team members SS, SJ, EA, JB, BG, BN, KP) differences were resolved by discussion. The following data categories were extracted: number of included studies, review PICO, setting of primary studies, and any analysis that took into account study setting or population characteristics, PTB outcomes, assessment of adverse effects and recommendations for practice and research. Preterm birth rates in LICs, LMCs, UMCs and HICs settings were drawn from data published in a rigorous review of national civil registration and vital statistics to determine global, regional and national estimates of levels of preterm birth.[6]

Where reported information allowed, we used the World Bank categories to identify the categories of all country settings identified in the reviews.[17] .

The population, interventions, comparators, outcomes and reviewer conclusions for future research were tabulated and described narratively. The country or countries of the included primary studies were noted, and the methods used in the review for analyses of data from different settings was also recorded and described. We did not contact review authors for missing data.

Patient and Public Involvement

This review was undertaken as part of a larger program of research in preterm birth (NIHR Global Health under grant (17/63/26)). The program is informed by key stakeholders and a PPI advisory group comprising of representatives from Sheffield, Bangladesh, and South Africa. The design and questions for the review were informed by consultation with these groups.

Results

Our search identified 3,133 citations which were screened by two reviewers. A third reviewer was also involved where there was a lack of consensus or uncertainty regarding inclusion. Following screening, 424 full text papers were retrieved for data extraction. At data extraction a further 285 were excluded. The process of identifying the included reviews is summarised in Figure 1.

We included 139 reviews which addressed a range of primary and secondary interventions and measured the effectiveness of the intervention in reducing the risk of PTB. These are summarised in Table 2 There was a considerable variation in the number of included studies in the reviews for each intervention, reflecting differing research questions objectives (therefore different PICOs) and search strategies.

Figure 1: Flow of studies through review process

For peer review only

Table 2 Summary of included systematic reviews and settings of primary studies included in the review

Interventions	Number of reviews	Number of primary studies	country NR	country of primary study LI	LM	UM	HI	mixed	studies where setting NK
**Primary prevention interventions**:									
**Health Systems**									
Models of antenatal care delivery (group/specialised)[18-28]	11	68	2	0	2	2	64	0	0
Midwifery led care[29]	1	15	0	0	0	0	15	0	0
Improving ANC coverage[30]	1	34	0	10	15	5	0	0	0
**Health behaviours**									
Smoking cessation[31 32]	2	111	0	0	0	1	110	0	0
Weight management[33-38]	6	70	1	0	2	8	60	0	0
**Nutritional interventions**									
Macronutrient supplements[39 40]	2	34	0	3	9	10	8	4	0
Micronutrient supplements[24-55]	33	481	2	29	82	122	214	6	9
Vitamin D[41-46]	6	75							
Vitamin A[47 48]	2	24							
Vitamin E, C, E and C[49-51]	3	67							
Iron, folic acid, iron and folic acid[52-59]	8	182							
Fish oil[60-64]	5	38							

Zinc[65 66]	2	25							
Calcium[67 68]	2	27							
Iodine[69]	2	14							
Multiple micronutrients[70-72]	3	29							
**Screening and treatment of periodontal disease[73-84]**	12	46	0	0	3	7	36	0	0
**Screening and prevention/treatment of infection**	14	91	2	2	2	6	79	0	2
Asymptomatic bacteriuria[85-88]	4								
Screening and antibiotics for syphilis[89]	1								
Influenza vaccine[90 91]	2								
Lower genital tract infection[92]	1								
UTI[93 94]	2								
Vaginal candidiasis[95]	1								
Nonspecific infection[96 97]	2								
Malaria[98-100]	3	17	0	8	7	2	2	0	0
**Secondary prevention interventions**:									
Cerclage[101-118]	18	123	10	0	7	11	42		51
Bed rest[119-121]	3	40	1	4	0	0	36	0	0
Cervical pessary[122-127]	6	16	0	0	0	1	14	1	0
Progesterone[128-143]	16	59	5	1	7	8	28	4	11
Tocolytics[144-156]	11	167	3	1	0	13	68	0	84

ANC: antenatal care, NK: not known, NR: not reported, LI: low income, LM: low middle, UM: upper middle, HI: high income, UTI: urinary tract infection.

For peer review only

Context of primary studies

A total of 1372 primary studies were included across all of the 139 reviews Not all of these studies will have been measuring PTB as an outcome but were included within the review which may have been measuring a range of maternal outcomes including PTB. The largest number of primary studies were those evaluating micronutrient supplements (n=481) and tocolytics (n=167). A total of 113 of the reviews described the country in which the primary studies were undertaken and so this data was known for 1288 (93.9%) of the 1372 included primary studies. Of these, 390 (30.3%) were undertaken in LMICs, fifteen primary studies were multicentre and included data gathered from LMIC and HIC settings, though only three of these studies included LICs. Of the studies undertaken in LMICs, a majority (n=255;) examined the effects of nutritional supplements. Excluding nutritional intervention studies, the proportion of LMIC-based primary studies of interventions to reduce PTB accounts for only (n=135) 10.5% of the included studies where settings are known..

Of the total number of primary studies undertaken in LMIC contexts, those studies undertaken in LIC settings represented a very small proportion of included studies. Participants from LICs were represented in only 4.5% (n=58) of the total number of studies, and if the nutritional intervention studies are excluded, they account for only 2.5% (n=32) of the studies evaluating interventions. Of those primary studies that were undertaken in LMIC settings the numbers within each country category differed significantly. The proportion of the studies that are undertaken in LIC, LMC and UMC were 14.9% (n=58), 34.8% (n=136) and 50.2% (n=196) respectively. There are only single trials that have evaluated the impact of progesterone, tocolytics and interventions to increase calorie intake in LIC settings. There are no trials that have evaluated smoking cessation, preventing excessive weight gain, prevention and treatment of periodontal disease, flu vaccine and cervical pessaries. The number of trials in each of the country categories within each intervention type are shown in Table 2.

When this data is compared alongside data that shows the prevalence of PTB globally it is clear that there is an inverse pattern in the distribution of the data (Figure 2).

Figure 2: Rates of PTB and proportion of primary studies undertaken in each setting.

The effectiveness of interventions

The effectiveness of interventions in reducing the risk of PTB was variable with no intervention showing consistent effectiveness across the included reviews. Although interpretation of this data is limited by the lack of quality appraisal of the included reviews, and therefore should be viewed with caution. Overall, the scoping review demonstrates considerable inconsistency of results of interventions. Of the 139 reviews, 28 reported a reduction in PTB in intervention versus a control, 80% (n=111) of the reviews found that the intervention had no impact in reducing the risk of PTB. The summary result (relative risk and odds ratio are shown in Figure 3). The results show the reduction in PTB less than 37 weeks gestation. In three reviews the intervention was not statistically significant at 37 weeks but was reported as statistically significant at 34 weeks[109], 35 weeks[134] and 36 weeks[128]. Two reviews reported a positive effect of the intervention in reducing risk of preterm birth but reported the outcome on a continuous measure. These included the effectiveness of macronutrient supplements[34] (SMD -0.19 (95% CI -0.34 to -0.04)) and cerclage (mean difference 95% CI 33.98 days

For peer review only

(17.88 to 50.08))[106]. The interventions reporting binary outcomes which appear to have the greatest effect (RR = 0.2-0.4) in reducing PTB are: antibiotics for asymptomatic bacteriuria[86] (RR = 0.34 (95% CI 0.11 to 0.62), the screening and treatment of syphilis[89] (RR = 0.36 (95% CI 0.27-0.47), and treatment of vaginal candidiasis[95] (RR = 0.36, (95% CI 0.17 to 0.75). Interventions with moderate effects (RR = 0.4-0.6) included treating lower genital tract infection[92] and vitamin D supplements.[46] Four of the reviews (Figure 2) with a positive effect of the intervention considered that the strength of evidence supporting the finding could be considered high and the finding reliable. None of these reviews included studies conducted in LIC settings, and only one included one study in a LMIC.

Figure 3 Summary results of systematic reviews of interventions showing reduction in risk of PTB

ANC: antenatal care, RR: relative risk, OR: odds ratio, LGT: lower genital tract, L,M,IC: low, low middle, upper middle income countries.

For peer review only

Dealing with context and generalisability within evidence synthesis

The authors of the included reviews used different approaches to dealing with the contextual variation when pooling data from primary studies, which was either to ignore, document, explore or control for differences. Twenty-seven reviews (23.8%) did not describe the setting of the primary study, ignoring variation in outcomes that may arise as a result of these differences. This occurred most frequently in reviews of cervical cerclage (see Table 2). The majority of the included reviews 86 (76.1%) documented the country in which the primary study was carried out either within the text, tables of study characteristics or in accompanying appendices, but this was not considered further in terms of its implications for the findings, or application for future practice or research.

Eight reviews[30 39 40 44 46 65 99 157] sought to explore the impact of geographical and economic context by undertaking a subgroup analysis comparing trials conducted in low income settings with those in high income settings or regression analysis with geographical regions as covariates (Africa, Americas, Southeast Asia, Europe, Eastern Mediterranean, Western Pacific). In addition, one study[157] listed the country instead of the author name on the forest plot allowing ready visualisation of differences across settings. Nine reviews[31 42 47 50 51 55 67 68 71] undertook subgroup analysis based on features of the population that might vary across settings and influence the effectiveness of the intervention, such as baseline nutritional status of the mother. One review[72] exploring multiple micronutrient supplementation controlled for settings by limiting the review to include only those studies undertaken in LMIC contexts. Four reviews[72 102 128 134] undertook an IPD (individual patient data) analysis, allowing subgroup analyses about differences in effect more easily than with aggregate data. This approach allowed comparison between effects for women recruited and receiving the intervention in different settings, effect sizes in each country could also be shown in the analyses.

DISCUSSION

This scoping review has revealed an inverse pattern of research, with only 30.3% of published research included in systematic reviews of interventions reporting PTB outcomes carried out in LMIC settings, and only 4.5% was conducted in the poorest countries in the world where the burden of PTB is greatest. The distribution of types of intervention tested and evaluated in these settings is not even across interventions, but is largely focused on very context specific interventions (prevention of malarial infection) and nutritional supplementation. Similar patterns of a mismatch between research effort and health needs in non- high income regions have been identified across a broad range of diseases.[158 159] It has also been previously reported that primary research often fails to capture those with the greatest health care needs such as vulnerable populations.[160 161]

This review has also revealed a limited approach in evidence synthesis to explore the applicability of findings across geographical settings and to draw attention to these gaps with a resultant risk that interventions shown to be effective in HI settings may not translate to LIC settings and may indeed have adverse effects when applied to LIC settings. Likewise, the focus of research in HIC settings means that interventions that may have greater benefit in LIC settings – where the problem is greatest – remain untested or replicated with larger numbers of participants. Adolescent pregnancy and short inter pregnancy intervals, both of which are more common in LMICs, have been highlighted as important risk factors for PTB[162] yet there is a lack of data on interventions to address these and their effectiveness in reducing the risk of PTB.

For peer review only

The lack of robust evidence to inform both the primary and secondary prevention of PTB in LIC settings, where the prevalence of PTB is highest presents challenges for developing appropriate and contextually relevant clinical guidance. . The factors that mean findings cannot be generalised from high resource settings to low and middle resource settings are multiple and will differ across interventions. Ethnicity, poverty, gender dynamics, pollution, temperature, climate, diet, access to health care, educational status, employment conditions are all examples of factors that might play a role in these differences. Improved understanding of the etiopathogenesis of PTB is also necessary for defining an accurate model of risk prediction and would help in understanding what factors in local settings increase risk and facilitate the development of an accurate model of risk prediction. [163]

Two recent overviews of reviews[9 10] also found that few interventions are effective in PTB prevention. The following interventions were identified in these reviews as showing positive or possible benefit: lifestyle and behavioural changes (including diet and exercise); nutritional supplements (including calcium, zinc and vitamin D supplementation); nutritional education; and screening for lower genital tract infections. Positive effects of secondary interventions were found for low dose aspirin among women at risk of preeclampsia; clindamycin for treatment of bacterial vaginosis; treatment of vaginal candidiasis; progesterone in women with prior spontaneous PTB and in those with short mid-trimester cervical length; L-arginine in women at risk for preeclampsia; levothyroxine among women with thyroid disease; calcium supplementation in women at risk of hypertensive disorders; smoking cessation; cervical length screening in women with history of PTB with placement of cerclage in those with short cervix; cervical pessary in singleton gestations with short cervix; and treatment of periodontal disease. Our review findings were in concordance, although, in addition, we identified screening and antibiotic treatment for syphilis, and positive effects of fish oil supplements. In most instances the trials were small and authors recommended larger well-designed RCTs. The lack of consistency across review findings for interventions also merits more exploration. Compromised methodological rigour can inflate trial findings by 30% to 50%.[164 165] Some of the differences in our review findings reflect some differences in the included reviews.

The interventions identified in this review, and those of Matei et al (2019)[9] and Medley et al (2019)[10] informing guideline development, clinical practice and policy decision making have been little tested in LMIC settings. In those interventions where there is more consistency in review findings such as cervical cerclage, there are no studies that have been conducted in low income settings and over half of the reviews did not report or consider settings in their analyses.

This scoping review has shown that many authors of systematic reviews fail to use design and statistical approaches that adequately address contextual variations between the included source studies and imperfectly represent 'real world' conditions within the target context (Higgins et al 2019). While those reviews that sought to take into account LMIC contexts were unable to conduct the analyses due to a lack of data, they nonetheless were able to highlight the gaps in research, for example the lack of studies in vitamin D undertaken in Africa.[44]

The Preferred Reporting Items for Systematic reviews and Meta-analyses (PRISMA) reporting standards reference 'context' in terms of the circumstances requiring the review itself, rather than referencing the contexts of studies included in the review.[166] The PRISMA extension for Complex Interventions includes the elements of 'time' and 'setting'.[167] However, grouping LMIC data, or even LI data may still be too broad. Even within the categories of LIC there is considerable diversity that

For peer review only

may impact on how an intervention works and within countries there may also be considerable diversity between the wealthiest and poorest groups. For example, the time taken to reach comprehensive emergency obstetric care facilities in low resource settings is often underestimated and for most women is likely to be 120 minutes of travel time.[168] Context cannot be standardised, it will vary from review to review, as different interventions and different populations are considered. 'Context' and the factors that might influence the efficacy, uptake, acceptability, appropriateness, accessibility and availability of an intervention requires a good understanding of the aetiology and mechanisms by which risk factors interact with environmental, microbial, socio-political and health system variations across settings.[169]

It must be acknowledged that there are significant barriers to undertaking research in many settings across the globe. These include very practical challenges such as a lack of access to high quality data and the challenges of estimating gestational age.[170] Recent changes to global health funding arena include a very large proportion being spent on the pandemic as well as government reductions, e.g. in the UK [171]. These reductions in funding will undermine what has been a growth in research in LMIC settings and will impede efforts to address the imbalances highlighted in this scoping review.

A number of limitations exist in this scoping review. We have not sought to identify the setting of primary studies where this is not reported in the systematic review. We have also not limited our analysis to studies within the reviews that only contributed findings to the risk of PTB. Most reviews explored several maternal and infant outcomes. Therefore, in this scoping review, included primary studies may not have contained PTB outcome data. We limited our scoping review to exploring evidence within systematic reviews as these are key sources of evidence to inform guideline development and policy decision making. It is possible that further primary studies have been published but are not included in this analysis. Nevertheless, it gives an indication of the distribution of research being undertaken in the poorest regions of the world that address PTB.

CONCLUSION

Only 4.5% of primary research to examine the effectiveness of interventions to reduce the risk of PTB is carried out in settings where the burden is greatest. No interventions which reduce the risk of PTB, judged to be supported by strong evidence, include studies undertaken in low resource settings. In the synthesis of studies, current methods often fail to address the contextual variation and consider the applicability of findings in low resource, high burden settings. This has implications for supporting policy making, and development of contextually relevant clinical guidelines. While methods can be undertaken to improve approaches to evidence synthesis, they cannot compensate for the lack of primary research in low resource settings. This is critical if global health inequalities are to be addressed and millennium development goals[172] to reduce under-five mortality are to be achieved. Funding and supporting research in LMICs would have a three-fold benefit; firstly, if the prevalence of the disease is higher it is easier to reach statistical significance for efficacy or inefficacy of each tested intervention. Secondly, it would address the knowledge gap highlighted in this review and finally – and most importantly – the implementation of effective interventions would have the potential for greater public health impact where the risks are greater, more prevalent and outcomes more severe.

For peer review only

Figure Headings

Figure 1: Flow of studies through review process

Figure 2: Rates of PTB and proportion of primary studies undertaken in each setting.

Figure 3 Summary results of systematic reviews of interventions showing reduction in risk of PTB

References

1. WHO. WHO: recommended definitions, terminology and format for statistical tables related to the perinatal period and use of a new certificate for cause of perinatal deaths. Modifications recommended by FIGO as amended October 14, 1976. . *Acta Obstet Gynecol Scand* 1977;1977; 56: 247–53.
2. Liu L, Oza S, Hogan D, et al. Global, regional, and national causes of child mortality in 2000–13, with projections to inform post-2015 priorities: an updated systematic analysis. *The Lancet* 2015;385(9966):430-40.
3. Brydges CR, Landes JK, Reid CL, et al. Cognitive outcomes in children and adolescents born very preterm: a meta-analysis. *Developmental Medicine & Child Neurology* 2018;60(5):452-68.
4. Coathup V, Boyle E, Carson C, et al. Gestational age and hospital admissions during childhood: population based, record linkage study in England (TIGAR study). *bmj* 2020;371
5. Howson CP, Kinney MV, McDougall L, et al. Born too soon: preterm birth matters. *Reproductive health* 2013;10(1):1-9.
6. Chawanpaiboon S, Vogel JP, Moller A-B, et al. Global, regional, and national estimates of levels of preterm birth in 2014: a systematic review and modelling analysis. *The Lancet Global Health* 2019;7(1):e37-e46.
7. Barros FC, Bhutta ZA, Batra M, et al. Global report on preterm birth and stillbirth (3 of 7): evidence for effectiveness of interventions. *BMC pregnancy and childbirth* 2010;10(1):1-36.
8. Bhutta ZA, Das JK, Bahl R, et al. Can available interventions end preventable deaths in mothers, newborn babies, and stillbirths, and at what cost? *The Lancet* 2014;384(9940):347-70.
9. Matei A, Saccone G, Vogel JP, et al. Primary and secondary prevention of preterm birth: a review of systematic reviews and ongoing randomized controlled trials. *European Journal of Obstetrics & Gynecology and Reproductive Biology* 2019;236:224-39.
10. Medley N, Vogel JP, Care A, et al. Interventions during pregnancy to prevent preterm birth: an overview of Cochrane systematic reviews. *Cochrane Database of Systematic Reviews* 2018(11)
11. Opiyo N, Stones W. Corticosteroids for preterm deliveries: missing evidence. *Cochrane Database Syst Rev* 2017;5:ED000121.
12. Egwuatu V. Complications of cervical cerclage in Igbo women. *Journal of the National Medical Association* 1986;78(3):245.
13. White H, Albers B, Gaarder M, et al. Guidance for producing a Campbell evidence and gap map. *Campbell Systematic Reviews* 2020;16(4):e1125.
14. Arksey H, O'Malley L. Scoping studies: towards a methodological framework. *International journal of social research methodology* 2005;8(1):19-32.

For peer review only - http://bmjopen.bmj.com/site/about/guidelines.xhtml

15. Allen F, Gray R, Oakley L, et al. Technical guide to the infant mortality evidence map: systematic reviews of interventions targeting major potentially modifiable risk factors for infant mortality. 2009
16. Sutton A, Campbell F. The ScHARR LMIC filter: adapting a low-and middle-income countries geographic search filter to identify studies on preterm birth prevention and management. *Research Synthesis Methods* 2022
17. Bank TW. world Bank country and Lending Groups 2021 [Available from: https://datahelpdesk.worldbank.org/knowledgebase/articles/906519 accessed 18/03/2021.
18. Allen J, Gamble J, Stapleton H, et al. Does the way maternity care is provided affect maternal and neonatal outcomes for young women? A review of the research literature (Structured abstract). *Women and Birth* 2012;25(2):54-63.
19. Catling CJ, Medley N, Foureur M, et al. Group versus conventional antenatal care for women. *Cochrane Database of Systematic Reviews* 2015(2):CD007622.
20. Dodd JM, Dowswell T, Crowther CA. Specialised antenatal clinics for women with a multiple pregnancy for improving maternal and infant outcomes. *Cochrane Database of Systematic Reviews* 2015(11) doi: 10.1002/14651858.CD005300.pub4
21. Dowswell T, Carroli G, Duley L, et al. Alternative versus standard packages of antenatal care for low-risk pregnancy. *Cochrane Database of Systematic Reviews* 2015(7):CD000934.
22. Dowswell T, Middleton P, Weeks A. Antenatal day care units versus hospital admission for women with complicated pregnancy. *Cochrane Database of Systematic Reviews* 2009(4) doi: 10.1002/14651858.CD001803.pub2
23. Fernandez Turienzo C, Sandall J, Peacock JL. Models of antenatal care to reduce and prevent preterm birth: a systematic review and meta-analysis. *BMJ Open* 2016;6(1):e009044.
24. Lathrop B. A systematic review comparing group prenatal care to traditional prenatal care. *Nursing for Women's Health* 2013;17(2):118-30.
25. Malouf R, Redshaw M. Specialist antenatal clinics for women at high risk of preterm birth: a systematic review of qualitative and quantitative research. *BMC Pregnancy & Childbirth* 2017;17(1):51.
26. Ruiz-Mirazo E, Lopez-Yarto M, McDonald SD. Group prenatal care versus individual prenatal care: a systematic review and meta-analyses. *Journal of Obstetrics & Gynaecology Canada: JOGC* 2012;34(3):223-29.
27. Sheeder J, Weber Yorga K, Kabir-Greher K. A review of prenatal group care literature: the need for a structured theoretical framework and systematic evaluation. *Maternal & Child Health Journal* 2012;16(1):177-87.
28. Whitworth M, Quenby S, Cockerill RO, et al. Specialised antenatal clinics for women with a pregnancy at high risk of preterm birth (excluding multiple pregnancy) to improve maternal and infant outcomes. *Cochrane Database of Systematic Reviews* 2011(9):CD006760.
29. Sandall J, Soltani H, Gates S, et al. Midwife-led continuity models versus other models of care for childbearing women. *Cochrane Database of Systematic Reviews* 2013(8):CD004667.
30. Mbuagbaw L, Medley N, Darzi AJ, et al. Health system and community level interventions for improving antenatal care coverage and health outcomes. *Cochrane Database of Systematic Reviews* 2015(12) doi: 10.1002/14651858.CD010994.pub2

For peer review only

31. Chamberlain C, O'Mara-Eves A, Oliver S, et al. Psychosocial interventions for supporting women to stop smoking in pregnancy. *Cochrane Database of Systematic Reviews* 2013(10):CD001055.
32. Coleman T, Chamberlain C, Davey MA, et al. Pharmacological interventions for promoting smoking cessation during pregnancy. *Cochrane Database of Systematic Reviews* 2012(9):CD010078.
33. Dodd J, Grivell R, Crowther C, et al. Antenatal interventions for overweight or obese pregnant women: a systematic review of randomised trials (Structured abstract). *BJOG An International Journal of Obstetrics and Gynaecology* 2010;117(11):1316-26.
34. Gresham E, Bisquera A, Byles JE, et al. Effects of dietary interventions on pregnancy outcomes: a systematic review and meta-analysis. *Maternal & Child Nutrition* 2016;12(1):5-23.
35. Muktabhant B, Lawrie TA, Lumbiganon P, et al. Diet or exercise, or both, for preventing excessive weight gain in pregnancy. *Cochrane Database of Systematic Reviews* 2015(6):CD007145.
36. Shepherd E, Gomersall JC, Tieu J, et al. Combined diet and exercise interventions for preventing gestational diabetes mellitus. *Cochrane Database of Systematic Reviews* 2017(11) doi: 10.1002/14651858.CD010443.pub3
37. Tanentsapf I, Heitmann BL, Adegboye AR. Systematic review of clinical trials on dietary interventions to prevent excessive weight gain during pregnancy among normal weight, overweight and obese women. *BMC Pregnancy & Childbirth* 2011;11:81.
38. Thangaratinam S, Rogozinska E, Jolly K, et al. Interventions to reduce or prevent obesity in pregnant women: a systematic review. *Health Technology Assessment (Winchester, England)* 2012;16(31):iii-iv, 1-191.
39. Girard AW, Olude O. Nutrition education and counselling provided during pregnancy: effects on maternal, neonatal and child health outcomes. *Paediatric and perinatal epidemiology* 2012;26:191-204.
40. Ota E, Hori H, Mori R, et al. Antenatal dietary education and supplementation to increase energy and protein intake. *Cochrane Database of Systematic Reviews* 2015(6):CD000032.
41. Bi WG, Nuyt AM, Weiler H, et al. Association Between Vitamin D Supplementation During Pregnancy and Offspring Growth, Morbidity, and Mortality: A Systematic Review and Meta-analysis. *JAMA Pediatrics* 2018;172(7):635-45.
42. Palacios C, De-Regil LM, Lombardo LK, et al. Vitamin D supplementation during pregnancy: Updated meta-analysis on maternal outcomes. *Journal of Steroid Biochemistry & Molecular Biology* 2016;164:148-55.
43. Perez-Lopez FR, Pasupuleti V, Mezones-Holguin E, et al. Effect of vitamin D supplementation during pregnancy on maternal and neonatal outcomes: a systematic review and meta-analysis of randomized controlled trials. *Fertility & Sterility* 2015;103(5):1278-88.e74.
44. Roth DE, Leung M, Mesfin E, et al. Vitamin D supplementation during pregnancy: state of the evidence from a systematic review of randomised trials. *Bmj* 2017;359:j5237.
45. Thorne-Lyman A, Fawzi WW. Vitamin D during pregnancy and maternal, neonatal and infant health outcomes: a systematic review and meta-analysis. *Paediatric and Perinatal Epidemiology* 2012;26 Suppl 1:75-90.

For peer review only

46. Zhou SS, Tao YH, Huang K, et al. Vitamin D and risk of preterm birth: Up-to-date meta-analysis of randomized controlled trials and observational studies. *Journal of Obstetrics & Gynaecology Research* 2017;43(2):247-56.
47. McCauley ME, van den Broek N, Dou L, et al. Vitamin A supplementation during pregnancy for maternal and newborn outcomes. *Cochrane Database of Systematic Reviews* 2015(10):CD008666.
48. Thorne-Lyman AL, Fawzi WW. Vitamin A and carotenoids during pregnancy and maternal, neonatal and infant health outcomes: a systematic review and meta-analysis. *Paediatric and Perinatal Epidemiology* 2012;26 Suppl 1:36-54.
49. Rahimi R, Nikfar S, Rezaie A, et al. A meta-analysis on the efficacy and safety of combined vitamin C and E supplementation in preeclamptic women. *Hypertension in Pregnancy* 2009;28(4):417-34.
50. Rumbold A, Ota E, Hori H, et al. Vitamin E supplementation in pregnancy. *Cochrane Database of Systematic Reviews* 2015(9):CD004069.
51. Rumbold A, Ota E, Nagata C, et al. Vitamin C supplementation in pregnancy. *Cochrane Database of Systematic Reviews* 2015(9):CD004072.
52. Lassi ZS, Salam RA, Haider BA, et al. Folic acid supplementation during pregnancy for maternal health and pregnancy outcomes. *Cochrane Database of Systematic Reviews* 2013(3):CD006896.
53. Mantovani E, Filippini F, Bortolus R, et al. Folic acid supplementation and preterm birth: results from observational studies. *BioMed Research International* 2014;2014:481914.
54. Pena-Rosas JP, De-Regil LM, Dowswell T, et al. Intermittent oral iron supplementation during pregnancy. *Cochrane Database of Systematic Reviews* 2012(7):CD009997.
55. Peña-Rosas JP, De-Regil LM, Garcia-Casal MN, et al. Daily oral iron supplementation during pregnancy. *Cochrane Database of Systematic Reviews* 2015(7) doi: 10.1002/14651858.CD004736.pub5
56. Pena-Rosas JP, Viteri FE. Effects and safety of preventive oral iron or iron+folic acid supplementation for women during pregnancy. *Cochrane Database of Systematic Reviews* 2009(4):CD004736.
57. Saccone G, Berghella V. Folic acid supplementation in pregnancy to prevent preterm birth: a systematic review and meta-analysis of randomized controlled trials. *European Journal of Obstetrics, Gynecology, & Reproductive Biology* 2016;199:76-81.
58. Zhang Q, Wang Y, Xin X, et al. Effect of folic acid supplementation on preterm delivery and small for gestational age births: A systematic review and meta-analysis. *Reproductive Toxicology* 2017;67:35-41.
59. Imdad A, Bhutta Z. Routine iron/folate supplementation during pregnancy: effect on maternal anaemia and birth outcomes (Structured abstract). *Paediatric and Perinatal Epidemiology* 2012;26(Supplement 1):168-77.
60. Chen B, Ji X, Zhang L, et al. Fish oil supplementation improves pregnancy outcomes and size of the newborn: a meta-analysis of 21 randomized controlled trials. *Journal of Maternal-Fetal & Neonatal Medicine* 2016;29(12):2017-27.
61. Kar S, Wong M, Rogozinska E, et al. Effects of omega-3 fatty acids in prevention of early preterm delivery: a systematic review and meta-analysis of randomized studies. *European Journal of Obstetrics, Gynecology, & Reproductive Biology* 2016;198:40-46.

For peer review only

62. Saccone G, Berghella V. Omega-3 supplementation to prevent recurrent preterm birth: a systematic review and metaanalysis of randomized controlled trials. *American Journal of Obstetrics & Gynecology* 2015;213(2):135-40.
63. Salvig JD, Lamont RF. Evidence regarding an effect of marine n-3 fatty acids on preterm birth: a systematic review and meta-analysis. *Acta Obstetricia et Gynecologica Scandinavica* 2011;90(8):825-38.
64. Saccone G, Saccone I, Berghella V. Omega-3 long-chain polyunsaturated fatty acids and fish oil supplementation during pregnancy: which evidence? *Journal of Maternal-Fetal & Neonatal Medicine* 2016;29(15):2389-97.
65. Chaffee BW, King JC. Effect of zinc supplementation on pregnancy and infant outcomes: a systematic review. *Paediatric and Perinatal Epidemiology* 2012;26 Suppl 1:118-37.
66. Mori R, Ota E, Middleton P, et al. Zinc supplementation for improving pregnancy and infant outcome. *Cochrane Database of Systematic Reviews* 2012(7):CD000230.
67. Hofmeyr GJ, Lawrie TA, Atallah ÁN, et al. Calcium supplementation during pregnancy for preventing hypertensive disorders and related problems. *Cochrane database of systematic reviews* 2018(10)
68. Hofmeyr GJ, Manyame S, Medley N, et al. Calcium supplementation commencing before or early in pregnancy, for preventing hypertensive disorders of pregnancy. *Cochrane Database of Systematic Reviews* 2019(9)
69. Harding KB, Pena-Rosas JP, Webster AC, et al. Iodine supplementation for women during the preconception, pregnancy and postpartum period. *Cochrane Database of Systematic Reviews* 2017;3:CD011761.
70. Fall CH, Fisher DJ, Osmond C, et al. Multiple micronutrient supplementation during pregnancy in low-income countries: a meta-analysis of effects on birth size and length of gestation. *Food & Nutrition Bulletin* 2009;30(4 Suppl):S533-46.
71. Keats EC, Haider BA, Tam E, et al. Multiple-micronutrient supplementation for women during pregnancy. *Cochrane Database of Systematic Reviews* 2019(3)
72. Smith ER, Shankar AH, Wu LS, et al. Modifiers of the effect of maternal multiple micronutrient supplementation on stillbirth, birth outcomes, and infant mortality: a meta-analysis of individual patient data from 17 randomised trials in low-income and middle-income countries. *The Lancet Global Health* 2017;5(11):e1090-e100.
73. Corbella S, Del Fabbro M, Taschieri S, et al. Periodontal disease and adverse pregnancy outcomes: A systematic review. *Italian Oral Surgery* 2012;11(4):132-46.
74. Fogacci MF, Vettore MV, Leao AT. The effect of periodontal therapy on preterm low birth weight: a meta-analysis. *Obstetrics & Gynecology* 2011;117(1):153-65.
75. George A, Shamim S, Johnson M, et al. Periodontal treatment during pregnancy and birth outcomes: a meta-analysis of randomised trials. *International Journal of Evidence-Based Healthcare* 2011;9(2):122-47.
76. Pimentel Lopes De Oliveira GJ, Amaral Fontanari L, Chaves De Souza JA, et al. Effect of periodontal treatment on the incidence of preterm delivery: a systematic review. *Minerva Stomatologica* 2010;59(10):543-50.
77. Polyzos N, Polyzos I, Zavos A, et al. Obstetric outcomes after treatment of periodontal disease during pregnancy: systematic review and meta-analysis (Structured abstract). *Bmj* 2010;341(2):c7017.
78. Rosa MI, Pires PD, Medeiros LR, et al. Periodontal disease treatment and risk of preterm birth: a systematic review and meta-analysis. *Cadernos de Saude Publica* 2012;28(10):1823-33.

For peer review only

79. Shah M, Muley A, Muley P. Effect of nonsurgical periodontal therapy during gestation period on adverse pregnancy outcome: a systematic review. *Journal of Maternal-Fetal & Neonatal Medicine* 2013;26(17):1691-95.
80. Uppal A, Uppal S, Pinto A, et al. The effectiveness of periodontal disease treatment during pregnancy in reducing the risk of experiencing preterm birth and low birth weight: a meta-analysis. *Journal of the American Dental Association* 2010;141(12):1423-34.
81. Kim AJ, Lo AJ, Pullin DA, et al. Scaling and root planing treatment for periodontitis to reduce preterm birth and low birth weight: a systematic review and meta-analysis of randomized controlled trials. *Journal of Periodontology* 2012;83(12):1508-19.
82. da Silva HEC, Stefani CM, de Santos Melo N, et al. Effect of intra-pregnancy nonsurgical periodontal therapy on inflammatory biomarkers and adverse pregnancy outcomes: a systematic review with meta-analysis. *Systematic Reviews* 2017;6(1):197.
83. Iheozor-Ejiofor Z, Middleton P, Esposito M, et al. Treating periodontal disease for preventing adverse birth outcomes in pregnant women. *Cochrane Database of Systematic Reviews* 2017;6:CD005297.
84. Schwendicke F, Karimbux N, Allareddy V, et al. Periodontal treatment for preventing adverse pregnancy outcomes: a meta- and trial sequential analysis. *PLoS ONE [Electronic Resource]* 2015;10(6):e0129060.
85. Guinto VT, De GB, Festin MR, et al. Different antibiotic regimens for treating asymptomatic bacteriuria in pregnancy. *Cochrane Database of Systematic Reviews* 2010(9) doi: 10.1002/14651858.CD007855.pub2
86. Smaill FM, Vazquez JC. Antibiotics for asymptomatic bacteriuria in pregnancy. *Cochrane Database of Systematic Reviews* 2015(8):CD000490.
87. Widmer M, Lopez I, Gülmezoglu AM, et al. Duration of treatment for asymptomatic bacteriuria during pregnancy. *Cochrane Database of Systematic Reviews* 2015(11)
88. Angelescu K, Nussbaumer-Streit B, Sieben W, et al. Benefits and harms of screening for and treatment of asymptomatic bacteriuria in pregnancy: a systematic review. *BMC Pregnancy & Childbirth* 2016;16(1):336.
89. Blencowe H, Cousens S, Kamb M, et al. Lives Saved Tool supplement detection and treatment of syphilis in pregnancy to reduce syphilis related stillbirths and neonatal mortality. *BMC Public Health* 2011;11 Suppl 3:S9.
90. Fell DB, Platt RW, Lanes A, et al. Fetal death and preterm birth associated with maternal influenza vaccination: systematic review. *BJOG: An International Journal of Obstetrics & Gynaecology* 2015;122(1):17-26.
91. Zhang C, Wang X, Liu D, et al. A systematic review and meta-analysis of fetal outcomes following the administration of influenza A/H1N1 vaccination during pregnancy. *International Journal of Gynaecology & Obstetrics* 2018;141(2):141-50.
92. Sangkomkamhang US, Lumbiganon P, Prasertcharoensuk W, et al. Antenatal lower genital tract infection screening and treatment programs for preventing preterm delivery. *Cochrane Database of Systematic Reviews* 2015(2) doi: 10.1002/14651858.CD006178.pub3
93. Schneeberger C, Geerlings SE, Middleton P, et al. Interventions for preventing recurrent urinary tract infection during pregnancy. *Cochrane Database of Systematic Reviews* 2015(7) doi: 10.1002/14651858.CD009279.pub3
94. Vazquez JC, Abalos E. Treatments for symptomatic urinary tract infections during pregnancy. *Cochrane Database of Systematic Reviews* 2011(1):CD002256.

For peer review only

95. Roberts CL, Algert CS, Rickard KL, et al. Treatment of vaginal candidiasis for the prevention of preterm birth: a systematic review and meta-analysis. *Systematic Reviews* 2015;4:31.
96. Flenady V, Hawley G, Stock OM, et al. Prophylactic antibiotics for inhibiting preterm labour with intact membranes. *Cochrane Database of Systematic Reviews* 2013(12):CD000246.
97. Thinkhamrop J, Hofmeyr GJ, Adetoro O, et al. Antibiotic prophylaxis during the second and third trimester to reduce adverse pregnancy outcomes and morbidity. *Cochrane Database of Systematic Reviews* 2015(6):CD002250.
98. "C. K. Manyando K, Dalessandro U, Okafor HU, et al. A systematic review of the safety and efficacy of artemether-lumefantrine against uncomplicated Plasmodium falciparum malaria during pregnancy. *Malaria Journal* 2012;11 (no pagination)(141)
99. Radeva-Petrova D, Kayentao K, ter Kuile FO, et al. Drugs for preventing malaria in pregnant women in endemic areas: any drug regimen versus placebo or no treatment. *Cochrane Database of Systematic Reviews* 2014(10):CD000169.
100. Gamble C, Ekwaru PJ, Garner P, et al. Insecticide-treated nets for the prevention of malaria in pregnancy: a systematic review of randomised controlled trials. *PLoS Med* 2007;4(3):e107.
101. Alfirevic Z, Stampalija T, Medley N. Cervical stitch (cerclage) for preventing preterm birth in singleton pregnancy. *Cochrane Database of Systematic Reviews* 2017;6:CD008991.
102. Berghella V, Ciardulli A, Rust OA, et al. Cerclage for sonographic short cervix in singleton gestations without prior spontaneous preterm birth: systematic review and meta-analysis of randomized controlled trials using individual patient-level data. *Ultrasound in Obstetrics & Gynecology* 2017;50(5):569-77.
103. Berghella V, Keeler SM, To MS, et al. Effectiveness of cerclage according to severity of cervical length shortening: a meta-analysis. *Ultrasound in Obstetrics & Gynecology* 2010;35(4):468-73.
104. Berghella V, Rafael TJ, Szychowski JM, et al. Cerclage for short cervix on ultrasonography in women with singleton gestations and previous preterm birth: a meta-analysis. *Obstetrics & Gynecology* 2011;117(3):663-71.
105. DeFranco E, Valent A, Newman T, et al. Adjunctive therapies to cerclage for the prevention of preterm birth: a systematic review (Provisional abstract). *Obstetrics and Gynecology International* 2013;2013(2):528158.
106. Ehsanipoor RM, Seligman NS, Saccone G, et al. Physical Examination-Indicated Cerclage: A Systematic Review and Meta-analysis. *Obstetrics & Gynecology* 2015;126(1):125-35.
107. Moawad GN, Tyan P, Bracke T, et al. Systematic Review of Transabdominal Cerclage Placed via Laparoscopy for the Prevention of Preterm Birth. *Journal of Minimally Invasive Gynecology* 2018;25(2):277-86.
108. Namouz S, Porat S, Okun N, et al. Emergency cerclage: literature review (Provisional abstract). *Obstetrical and Gynecological Survey* 2013;68(5):379-88.
109. Pergialiotis V, Vlachos DG, Prodromidou A, et al. Double versus single cervical cerclage for the prevention of preterm births. *Journal of Maternal-Fetal & Neonatal Medicine* 2015;28(4):379-85.
110. Rafael TJ, Berghella V, Alfirevic Z. Cervical stitch (cerclage) for preventing preterm birth in multiple pregnancy. *Cochrane Database of Systematic Reviews* 2014(9):CD009166.

111. Saccone G, Rust O, Althuisius S, et al. Cerclage for short cervix in twin pregnancies: systematic review and meta-analysis of randomized trials using individual patient-level data. *Acta Obstetricia et Gynecologica Scandinavica* 2015;94(4):352-58.
112. Smith J, DeFranco EA. Tocolytics used as adjunctive therapy at the time of cerclage placement: a systematic review. *Journal of Perinatology* 2015;35(8):561-65.
113. Zeybek B, Hill A, Menderes G, et al. Robot-Assisted Abdominal Cerclage During Pregnancy. *Journal of the Society of Laparoendoscopic Surgeons* 2016;20(4):Oct-Dec.
114. Liu X, Luo X, Xiao X, et al. Cervical cerclage for preventing preterm birth in twin pregnancies. A systematic review and meta-analysis (Provisional abstract). *Database of Abstracts of Reviews of Effects* 2013(4):632-38.
115. Conde-Agudelo A, Romero R, Da Fonseca E, et al. Vaginal progesterone is as effective as cervical cerclage to prevent preterm birth in women with a singleton gestation, previous spontaneous preterm birth, and a short cervix: updated indirect comparison meta-analysis. *American Journal of Obstetrics & Gynecology* 2018;219(1):10-25.
116. Conde-Agudelo A, Romero R, Nicolaides K, et al. Vaginal progesterone vs. cervical cerclage for the prevention of preterm birth in women with a sonographic short cervix, previous preterm birth, and singleton gestation: a systematic review and indirect comparison metaanalysis. *American Journal of Obstetrics & Gynecology* 2013;208(1):42.e41-42.e18.
117. Jarde A, Lutsiv O, Park CK, et al. Preterm birth prevention in twin pregnancies with progesterone, pessary, or cerclage: a systematic review and meta-analysis. *BJOG: An International Journal of Obstetrics & Gynaecology* 2017;124(8):1163-73.
118. Jarde A, Lutsiv O, Park CK, et al. Effectiveness of progesterone, cerclage and pessary for preventing preterm birth in singleton pregnancies: a systematic review and network meta-analysis. *BJOG: An International Journal of Obstetrics & Gynaecology* 2017;124(8):1176-89.
119. Crowther CA, Han S. Hospitalisation and bed rest for multiple pregnancy. *Cochrane Database of Systematic Reviews* 2010(7):CD000110.
120. Maloni J. Antepartum bed rest for pregnancy complications: efficacy and safety for preventing preterm birth (Provisional abstract). *Biological Research for Nursing* 2010;12(2):106-24.
121. Sosa CG, Althabe F, Belizan JM, et al. Bed rest in singleton pregnancies for preventing preterm birth. *Cochrane Database of Systematic Reviews* 2015(3):CD003581.
122. Jin Z, Chen L, Qiao D, et al. Cervical pessary for preventing preterm birth: a meta-analysis. *Journal of Maternal-Fetal and Neonatal Medicine* 2017:1-7.
123. Liem SMS, van Pampus MG, Mol BWJ, et al. Cervical Pessaries for the Prevention of Preterm Birth: A Systematic Review. *Obstetrics and Gynecology International* 2013;2013:576723. doi: 10.1155/2013/576723
124. Saccone G, Ciardulli A, Xodo S, et al. Cervical pessary for preventing preterm birth in twin pregnancies with short cervical length: a systematic review and meta-analysis. *Journal of Maternal-Fetal & Neonatal Medicine* 2017;30(24):2918-25.
125. Saccone G, Ciardulli A, Xodo S, et al. Cervical Pessary for Preventing Preterm Birth in Singleton Pregnancies with Short Cervical Length: A Systematic Review and Meta-analysis. *Obstetrical and Gynecological Survey* 2018;73(1):13-14.

For peer review only

126. Thangatorai R, Lim FC, Nalliah S. Cervical pessary in the prevention of preterm births in multiple pregnancies with a short cervix: PRISMA compliant systematic review and meta-analysis. *Journal of Maternal-Fetal & Neonatal Medicine* 2018;31(12):1638-45.
127. Zheng L, Dong J, Dai Y, et al. Cervical pessaries for the prevention of preterm birth: a systematic review and meta-analysis. *Journal of Maternal-Fetal & Neonatal Medicine* 2017:1-10.
128. Romero R, Conde-Agudelo A, Da Fonseca E, et al. Vaginal progesterone for preventing preterm birth and adverse perinatal outcomes in singleton gestations with a short cervix: a meta-analysis of individual patient data. *American Journal of Obstetrics & Gynecology* 2018;218(2):161-80.
129. Romero R, Nicolaides KH, Conde-Agudelo A, et al. Vaginal progesterone decreases preterm birth<=34weeks of gestation in women with a singleton pregnancy and a short cervix: an updated meta-analysis including data from the OPPTIMUM study. *Ultrasound in Obstetrics & Gynecology* 2016;48(3):308-17.
130. Saccone G, Khalifeh A, Elimian A, et al. Vaginal progesterone vs intramuscular 17alpha-hydroxyprogesterone caproate for prevention of recurrent spontaneous preterm birth in singleton gestations: systematic review and meta-analysis of randomized controlled trials. *Ultrasound in Obstetrics & Gynecology* 2017;49(3):315-21.
131. Su LL, Samuel M, Chong YS. Progestational agents for treating threatened or established preterm labour. *Cochrane Database of Systematic Reviews* 2010(1):CD006770.
132. Suhag A, Saccone G, Berghella V. Vaginal progesterone for maintenance tocolysis: a systematic review and metaanalysis of randomized trials. *American Journal of Obstetrics & Gynecology* 2015;213(4):479-87.
133. Dodd JM, Grivell RM, Obrien CM, et al. Prenatal administration of progestogens for preventing spontaneous preterm birth in women with a multiple pregnancy. *Cochrane Database of Systematic Reviews* 2019(11) doi: 10.1002/14651858.CD012024.pub3
134. Romero R, Conde-Agudelo A, El-Refaie W, et al. Vaginal progesterone decreases preterm birth and neonatal morbidity and mortality in women with a twin gestation and a short cervix: an updated meta-analysis of individual patient data. *Ultrasound in Obstetrics & Gynecology* 2017;49(3):303-14.
135. Sotiriadis A, Papatheodorou S, Makrydimas G. Perinatal outcome in women treated with progesterone for the prevention of preterm birth: a meta-analysis. *Ultrasound in Obstetrics & Gynecology* 2012;40(3):257-66.
136. Lim CE, Ho KK, Cheng NC, et al. Combined oestrogen and progesterone for preventing miscarriage. *Cochrane Database of Systematic Reviews* 2013(9):CD009278.
137. Norman JE, Mackenzie F, Owen P, et al. Progesterone for the prevention of preterm birth in twin pregnancy (STOPPIT): A randomized, double-blind, placebo-controlled study and meta-analysis. *Obstetrical and Gynecological Survey* 2009;64(10):646-48.
138. Likis FE, Edwards DR, Andrews JC, et al. Progestogens for preterm birth prevention: a systematic review and meta-analysis. *Obstetrics & Gynecology* 2012;120(4):897-907.
139. Palacio M, Ronzoni S, Sanchez-Ramos L, et al. Progestogens as Maintenance Treatment in Arrested Preterm Labor: A Systematic Review and Meta-analysis. *Obstetrics & Gynecology* 2016;128(5):989-1000.
140. Prior M, Hibberd R, Asemota N, et al. Inadvertent P-hacking among trials and systematic reviews of the effect of progestogens in pregnancy? A systematic review

For peer review only

and meta-analysis. *BJOG: An International Journal of Obstetrics and Gynaecology* 2017;124(7):1008-15.
141. Rode L, Langhoff-Roos J, Andersson C, et al. Systematic review of progesterone for the prevention of preterm birth in singleton pregnancies. *Acta Obstetricia et Gynecologica Scandinavica* 2009;88(11):1180-89.
142. Schmouder VM, Prescott GM, Franco A, et al. The rebirth of progesterone in the prevention of preterm labor. *Annals of Pharmacotherapy* 2013;47(4):527-36.
143. Velez Edwards DR, Likis FE, Andrews JC, et al. Progestogens for preterm birth prevention: a systematic review and meta-analysis by drug route. *Archives of Gynecology & Obstetrics* 2013;287(6):1059-66.
144. Chawanpaiboon S, Laopaiboon M, Lumbiganon P, et al. Terbutaline pump maintenance therapy after threatened preterm labour for reducing adverse neonatal outcomes. *Cochrane Database of Systematic Reviews* 2014(3):CD010800.
145. Crowther CA, Brown J, McKinlay CJ, et al. Magnesium sulphate for preventing preterm birth in threatened preterm labour. *Cochrane Database of Systematic Reviews* 2014(8):CD001060.
146. Gaudet LM, Singh K, Weeks L, et al. Effectiveness of terbutaline pump for the prevention of preterm birth. A systematic review and meta-analysis. *PLoS ONE [Electronic Resource]* 2012;7(2):e31679.
147. Haas D, Caldwell D, Kirkpatrick P, et al. Tocolytic therapy for preterm delivery: systematic review and network meta-analysis (Structured abstract). *Bmj* 2012;344(2):e6226.
148. McNamara HC, Crowther CA, Brown J. Different treatment regimens of magnesium sulphate for tocolysis in women in preterm labour. *Cochrane Database of Systematic Reviews* 2015(12):CD011200.
149. Vogel JP, Nardin JM, Dowswell T, et al. Combination of tocolytic agents for inhibiting preterm labour. *Cochrane Database of Systematic Reviews* 2014(7):CD006169.
150. Dodd JM, Crowther CA, Middleton P. Oral betamimetics for maintenance therapy after threatened preterm labour. *Cochrane Database of Systematic Reviews* 2012;12:CD003927.
151. Flenady V, Reinebrant HE, Liley HG, et al. Oxytocin receptor antagonists for inhibiting preterm labour. *Cochrane Database of Systematic Reviews* 2014(6):CD004452.
152. Giorgino FL, Egan CG. Use of isoxsuprine hydrochloride as a tocolytic agent in the treatment of preterm labour: a systematic review of previous literature. *Arzneimittel-Forschung* 2010;60(7):415-20.
153. Mackeen AD, Seibel-Seamon J, Grimes-Dennis J, et al. Tocolytics for preterm premature rupture of membranes. *Cochrane Database of Systematic Reviews* 2011(10):CD007062.
154. Saccone G, Suhag A, Berghella V. 17-alpha-hydroxyprogesterone caproate for maintenance tocolysis: a systematic review and metaanalysis of randomized trials. *American Journal of Obstetrics & Gynecology* 2015;213(1):16-22.
155. van Vliet EOG, Dijkema GH, Schuit E, et al. Nifedipine maintenance tocolysis and perinatal outcome: an individual participant data meta-analysis. *BJOG: An International Journal of Obstetrics and Gynaecology* 2016;123(11):1753-60.
156. Yamasmit W, Chaithongwongwatthana S, Tolosa JE, et al. Prophylactic oral betamimetics for reducing preterm birth in women with a twin pregnancy. *Cochrane Database of Systematic Reviews* 2012(9):CD004733.

For peer review only

157. Ota E, Mori R, Middleton P, et al. Zinc supplementation for improving pregnancy and infant outcome. *Cochrane Database of Systematic Reviews* 2015(2)
158. Bellows BW, Conlon CM, Higgs ES, et al. A taxonomy and results from a comprehensive review of 28 maternal health voucher programmes. *Journal of health, population, and nutrition* 2013;31(4 Suppl 2):S106.
159. Atal I, Trinquart L, Ravaud P, et al. A mapping of 115,000 randomized trials revealed a mismatch between research effort and health needs in non–high-income regions. *Journal of clinical epidemiology* 2018;98:123-32.
160. Dab W. Commentary on SPHERE (Strengthening Public Health Research in Europe) literature reviews. *European journal of public health* 2007;17(suppl_1):8-9.
161. Shepherd V. Research involving adults lacking capacity to consent: the impact of research regulation on 'evidence biased'medicine. *BMC Medical Ethics* 2016;17(1):1-8.
162. Organization WH. Born too soon: the global action report on preterm birth. 2012
163. Della Rosa PA, Miglioli C, Caglioni M, et al. A hierarchical procedure to select intrauterine and extrauterine factors for methodological validation of preterm birth risk estimation. *BMC pregnancy and childbirth* 2021;21(1):1-17.
164. Linde K, Scholz M, Ramirez G, et al. Impact of study quality on outcome in placebo-controlled trials of homeopathy. *Journal of clinical epidemiology* 1999;52(7):631-36.
165. Schulz KF, Chalmers I, Hayes RJ, et al. Empirical evidence of bias: dimensions of methodological quality associated with estimates of treatment effects in controlled trials. *Jama* 1995;273(5):408-12.
166. Moher D, Liberati A, Tetzlaff J, et al. Preferred reporting items for systematic reviews and meta-analyses: the PRISMA statement. *PLoS medicine* 2009;6(7):e1000097.
167. Guise J-M, Butler ME, Chang C, et al. AHRQ series on complex intervention systematic reviews—paper 6: PRISMA-CI extension statement and checklist. *Journal of clinical epidemiology* 2017;90:43-50.
168. Banke-Thomas A, Wong KL, Ayomoh FI, et al. "In cities, it's not far, but it takes long": comparing estimated and replicated travel times to reach life-saving obstetric care in Lagos, Nigeria. *BMJ Global Health* 2021;6(1):e004318.
169. Rogers L, De Brún A, McAuliffe E. Defining and assessing context in healthcare implementation studies: a systematic review. *BMC Health Services Research* 2020;20(1):591. doi: 10.1186/s12913-020-05212-7
170. Vogel JP, Chawanpaiboon S, Moller A-B, et al. The global epidemiology of preterm birth. *Best Practice & Research Clinical Obstetrics & Gynaecology* 2018;52:3-12.
171. C S. UKRI Official Development Assistance letter 11 March 2021: UKRI; 2021 [Available from: https://www.ukri.org/our-work/ukri-oda-letter-11-march-2021/ accessed March 23 2021.
172. Organization WH. Millennium development goals. 2008

For peer review only

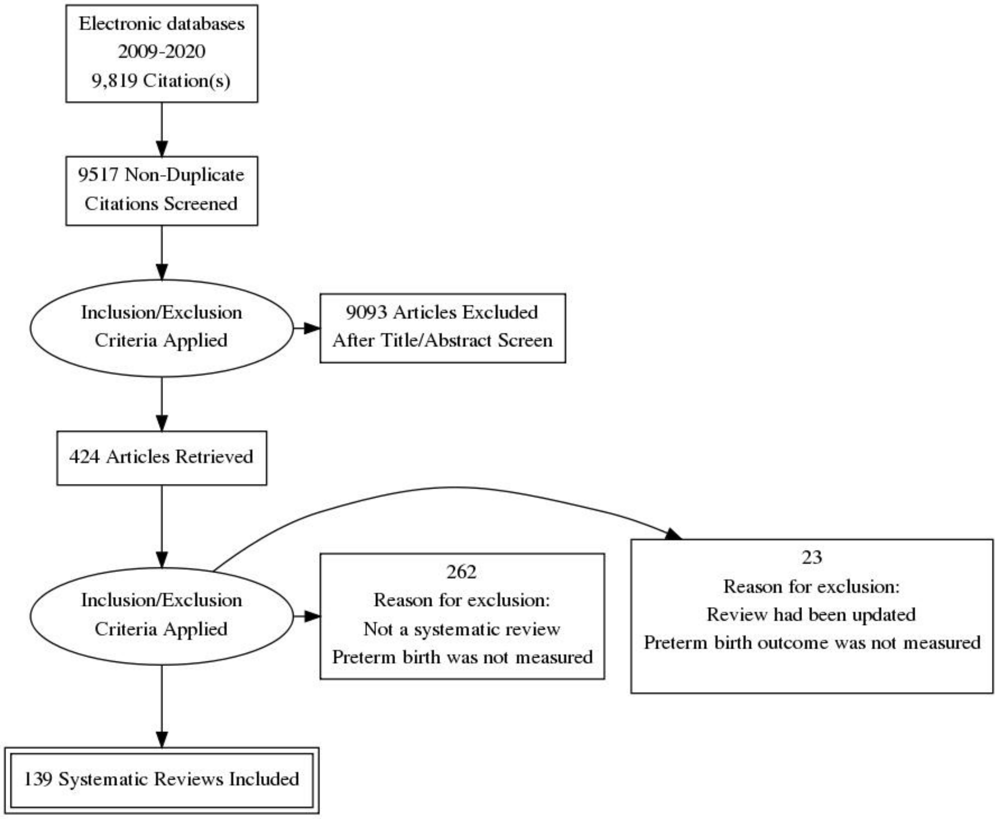

314x258mm (72 x 72 DPI)

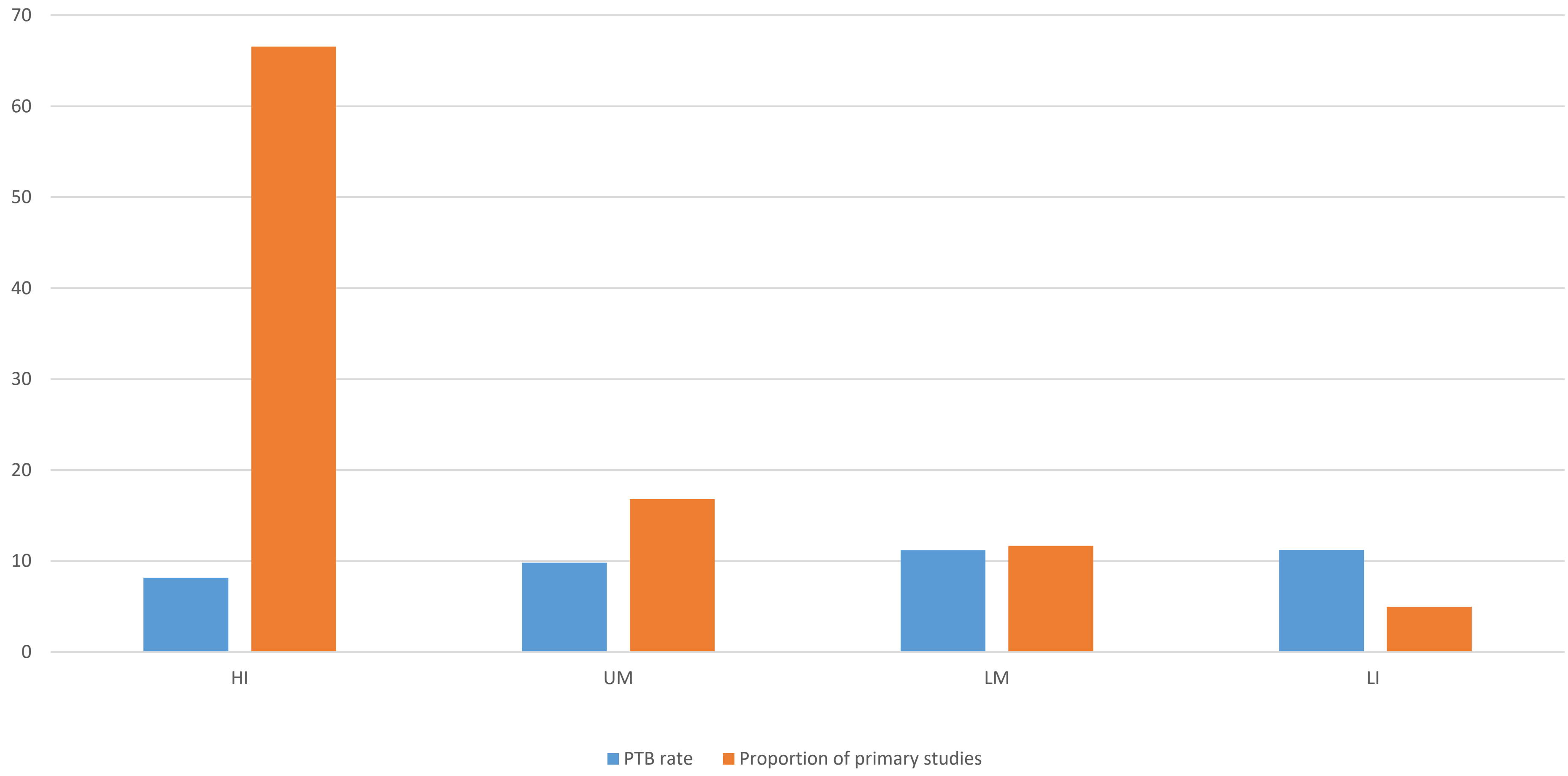
70
60
50
40
30
20
10
0
HI
UM
LM
LI
PTB rate
Proportion of primary studies

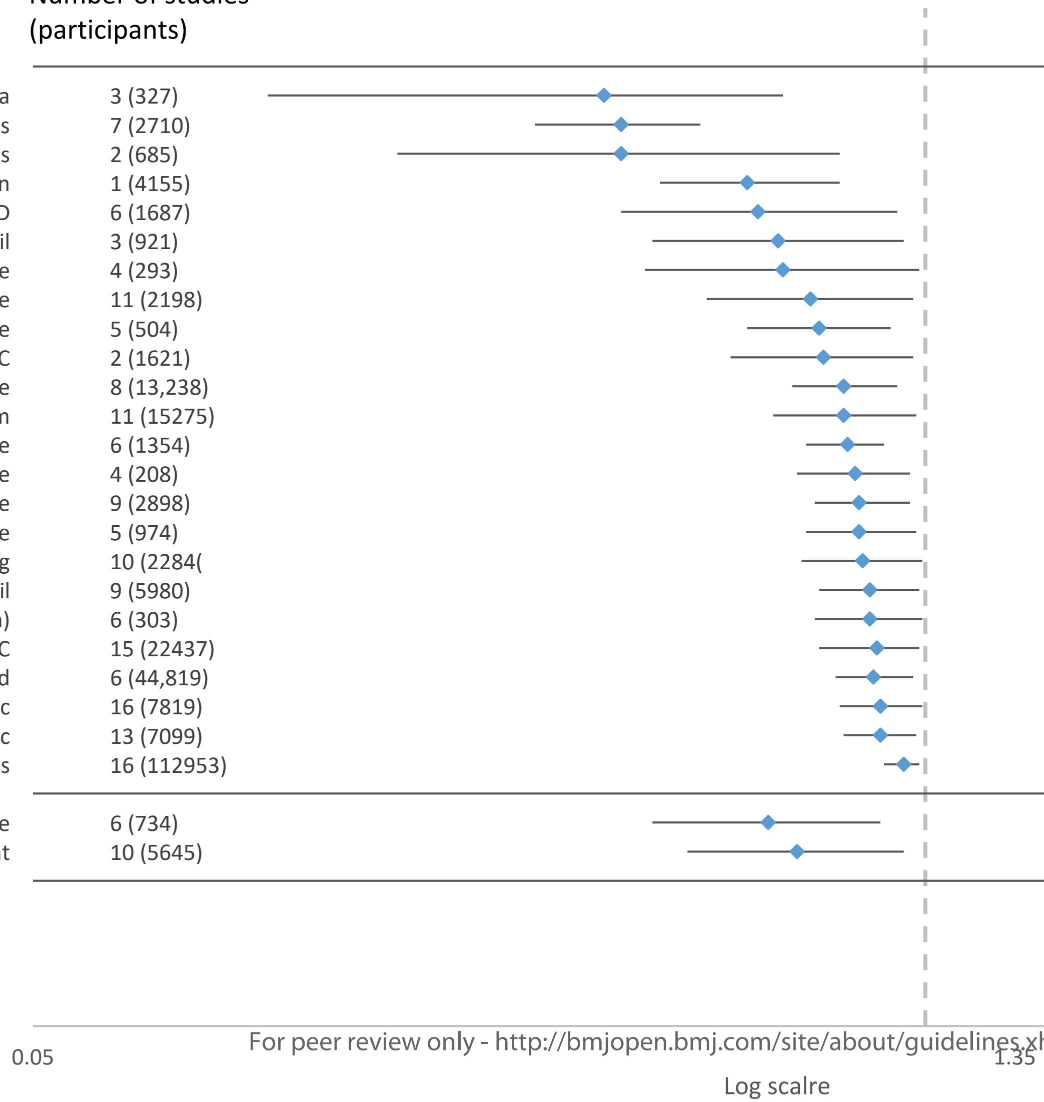

Relative Risk	Number of studies (participants)	Summary Data	L,U,M,IC	Grading
ABs for asymptomatic bacteriuria	3 (327)	0.34 [0.11, 0.62]	0,0,3	L
Screening and ABs for syphilis	7 (2710)	0.36 [0.27, 0.47]	1,1,5	L
Treatment of vaginal candidiasis	2 (685)	0.36 [0.17, 0.75]	0,0,0	H
Treating LGT infection	1 (4155)	0.55 [0.41, 0.75]	0,0,0	L
Vitamin D	6 (1687)	0.57 [0.36, 0.91]	0,0,0	L
Fish oil	3 (921)	0.61 [0.40, 0.93]	0,0,0	M
Progesterone	4 (293)	0.62 [0.39, 0.98]	0,0,1	L
Diet and exercise	11 (2198)	0.68 [0.48, 0.96]	0,0,0	Mx
Cerclage	5 (504)	0.70 [0.55, 0.89]	0,0,0	H
Alternative models of ANC	2 (1621)	0.71 [0.52, 0.96]	0,0,1	L
midwife-led models of care	8 (13,238)	0.76 [0.64, 0.91]	0,0,0	H
Calcium	11 (15275)	0.76 [0.60, 0.97]	0,2,3	L
Progesterone	6 (1354)	0.77 [0.67, 0.87]	0,0,1	U
Cerclage	4 (208)	0.79 [0.65, 0.95]	0,0,0	NR
Cerclage	9 (2898)	0.80 [0.69, 0.95]	0,1,0	H
Progesterone	5 (974)	0.80 [0.67, 0.97]	0,0,0	H
Macronutrients - counselling	10 (2284(	0.81 [0.66, 0.99]	0,0,1	L
fish oil	9 (5980)	0.83 [0.70, 0.98]	0,0,0	M
Progesterone (twin)	6 (303)	0.83 [0.69, 0.99]	0,1,2	M
Alternative models of ANC	15 (22437)	0.85 [0.70, 0.98]	0,0,0	M
Folic acid	6 (44,819)	0.84 [0.74, 0.96]	0,0,1	L
Zinc	16 (7819)	0.86 [0.75, 0.99]	2,5,2	L
zinc	13 (7099)	0.86 [0.76, 0.97]	1,4,3	M
Micronutrients	16 (112953)	0.93 [0.87, 0.98]	8,5,3	M
**Odds Ratio**				
Double vs single cerclage	6 (734)	0.59 [0.40, 0.86]	0,0,0	L
Periodontal disease treatment	10 (5645)	0.65 [0.45, 0.93]	0.1.3	M

Preferred Reporting Items for Systematic reviews and Meta-Analyses extension for Scoping Reviews (PRISMA-ScR) Checklist

SECTION	ITEM	PRISMA-ScR CHECKLIST ITEM	REPORTED ON PAGE #
**TITLE**			
Title	1	Identify the report as a scoping review.	Yes
**ABSTRACT**			
Structured summary	2	Provide a structured summary that includes (as applicable): background, objectives, eligibility criteria, sources of evidence, charting methods, results, and conclusions that relate to the review questions and objectives.	Yes
**INTRODUCTION**			
Rationale	3	Describe the rationale for the review in the context of what is already known. Explain why the review questions/objectives lend themselves to a scoping review approach.	Yes
Objectives	4	Provide an explicit statement of the questions and objectives being addressed with reference to their key elements (e.g., population or participants, concepts, and context) or other relevant key elements used to conceptualize the review questions and/or objectives.	Yes
**METHODS**			
Protocol and registration	5	Indicate whether a review protocol exists; state if and where it can be accessed (e.g., a Web address); and if available, provide registration information, including the registration number.	unpublished protocol which is available on request to the
Eligibility criteria	6	Specify characteristics of the sources of evidence used as eligibility criteria (e.g., years considered, language, and publication status), and provide a rationale.	Yes
Information sources*	7	Describe all information sources in the search (e.g., databases with dates of coverage and contact with authors to identify additional sources), as well as the date the most recent search was executed.	Yes
Search	8	Present the full electronic search strategy for at least 1 database, including any limits used, such that it could be repeated.	Yes
Selection of sources of evidence†	9	State the process for selecting sources of evidence (i.e., screening and eligibility) included in the scoping review.	Yes
Data charting process‡	10	Describe the methods of charting data from the included sources of evidence (e.g., calibrated forms or forms that have been tested by the team before their use, and whether data charting was done independently or in duplicate) and any processes for obtaining and confirming data from investigators.	Yes
Data items	11	List and define all variables for which data were sought and any assumptions and simplifications made.	Yes
Critical appraisal of individual sources of evidence§	12	If done, provide a rationale for conducting a critical appraisal of included sources of evidence; describe the methods used and how this information was used in any data synthesis (if appropriate).	We did not undertake quality appraisal
Synthesis of results	13	Describe the methods of handling and summarizing the data that were charted.	Yes

St. Michael's
Inspired Care.
Inspiring Science.

For peer review only - http://bmjopen.bmj.com/site/about/guidelines.xhtml

SECTION	ITEM	PRISMA-ScR CHECKLIST ITEM	REPORTED ON PAGE #
**RESULTS**			
Selection of sources of evidence	14	Give numbers of sources of evidence screened, assessed for eligibility, and included in the review, with reasons for exclusions at each stage, ideally using a flow diagram.	Yes
Characteristics of sources of evidence	15	For each source of evidence, present characteristics for which data were charted and provide the citations.	Yes
Critical appraisal within sources of evidence	16	If done, present data on critical appraisal of included sources of evidence (see item 12).	Critical appraisal was not undertaken
Results of individual sources of evidence	17	For each included source of evidence, present the relevant data that were charted that relate to the review questions and objectives.	Yes
Synthesis of results	18	Summarize and/or present the charting results as they relate to the review questions and objectives.	Yes
**DISCUSSION**			
Summary of evidence	19	Summarize the main results (including an overview of concepts, themes, and types of evidence available), link to the review questions and objectives, and consider the relevance to key groups.	Yes
Limitations	20	Discuss the limitations of the scoping review process.	Yes
Conclusions	21	Provide a general interpretation of the results with respect to the review questions and objectives, as well as potential implications and/or next steps.	Yes
**FUNDING**			
Funding	22	Describe sources of funding for the included sources of evidence, as well as sources of funding for the scoping review. Describe the role of the funders of the scoping review.	Yes

JBI = Joanna Briggs Institute; PRISMA-ScR = Preferred Reporting Items for Systematic reviews and Meta-Analyses extension for Scoping Reviews.

* Where *sources of evidence* (see second footnote) are compiled from, such as bibliographic databases, social media platforms, and Web sites.

† A more inclusive/heterogeneous term used to account for the different types of evidence or data sources (e.g., quantitative and/or qualitative research, expert opinion, and policy documents) that may be eligible in a scoping review as opposed to only studies. This is not to be confused with *information sources* (see first footnote).

‡ The frameworks by Arksey and O'Malley (6) and Levac and colleagues (7) and the JBI guidance (4, 5) refer to the process of data extraction in a scoping review as data charting.

§ The process of systematically examining research evidence to assess its validity, results, and relevance before using it to inform a decision. This term is used for items 12 and 19 instead of "risk of bias" (which is more applicable to systematic reviews of interventions) to include and acknowledge the various sources of evidence that may be used in a scoping review (e.g., quantitative and/or qualitative research, expert opinion, and policy document).

From: Tricco AC, Lillie E, Zarin W, O'Brien KK, Colquhoun H, Levac D, et al. PRISMA Extension for Scoping Reviews (PRISMAScR): Checklist and Explanation. Ann Intern Med. 2018;169:467–473. doi: 10.7326/M18-0850.

For peer review only - http://bmjopen.bmj.com/site/about/guidelines.xhtml
